# Development of Activity Rules and Chemical Fragment Design for In Silico Discovery of AChE and BACE1 Dual Inhibitors against Alzheimer’s Disease

**DOI:** 10.3390/molecules28083588

**Published:** 2023-04-20

**Authors:** Le-Quang Bao, Daniel Baecker, Do Thi Mai Dung, Nguyen Phuong Nhung, Nguyen Thi Thuan, Phuong Linh Nguyen, Phan Thi Phuong Dung, Tran Thi Lan Huong, Bakhtiyor Rasulev, Gerardo M. Casanola-Martin, Nguyen-Hai Nam, Hai Pham-The

**Affiliations:** 1 Department of Pharmaceutical Chemistry, Hanoi University of Pharmacy, 13-15 Le Thanh Tong, Hoan Kiem, Hanoi 10000, Vietnam; 2 Department of Pharmaceutical and Medicinal Chemistry, Institute of Pharmacy, University of Greifswald, Friedrich-Ludwig-Jahn-Straße 17, 17489 Greifswald, Germany; 3 College of Computing & Informatics, Drexel University, 3141 Chestnut St., Philadelphia, PA 19104, USA; 4 Department of Coatings and Polymeric Materials, North Dakota State University, Fargo, ND 58102, USA

**Keywords:** Alzheimer’s disease, QSAR, AChE, BACE1, dual-target inhibitor, fragment design

## Abstract

Multi-target drug development has become an attractive strategy in the discovery of drugs to treat of Alzheimer’s disease (AzD). In this study, for the first time, a rule-based machine learning (ML) approach with classification trees (CT) was applied for the rational design of novel dual-target acetylcholinesterase (AChE) and *β*-site amyloid-protein precursor cleaving enzyme 1 (BACE1) inhibitors. Updated data from 3524 compounds with AChE and BACE1 measurements were curated from the ChEMBL database. The best global accuracies of training/external validation for AChE and BACE1 were 0.85/0.80 and 0.83/0.81, respectively. The rules were then applied to screen dual inhibitors from the original databases. Based on the best rules obtained from each classification tree, a set of potential AChE and BACE1 inhibitors were identified, and active fragments were extracted using Murcko-type decomposition analysis. More than 250 novel inhibitors were designed in silico based on active fragments and predicted AChE and BACE1 inhibitory activity using consensus QSAR models and docking validations. The rule-based and ML approach applied in this study may be useful for the in silico design and screening of new AChE and BACE1 dual inhibitors against AzD.

## 1. Introduction

Alzheimer’s disease (AzD), the most common form of dementia, is characterized by memory problems and other cognitive impairments that seriously affect executive functions [1]. By 2023, an estimated 6.7 million Americans aged 65 and older have Alzheimer’s dementia, with most cases occurring in people who are 75 or older [2]. This number could increase to 13.8 million by 2060 if no medical breakthroughs are made to prevent, slow, or cure AzD. Due to higher average life expectancy, AzD is considered one of the greatest health threats contributing to the global burden of non-communicable diseases [3].

In AzD, certain neurons in the brain regions responsible for cognitive functions (e.g., thinking, learning, and memory) are gradually damaged or destroyed, leading to severe dementia symptoms [4]. There are two widely accepted hypotheses of AzD, namely the cholinergic hypothesis and the amyloid cascade [4]. The cholinergic hypothesis is supported by the observation that acetylcholine levels, a neurotransmitter that plays an important role in neuromodulation of learning, memory, and cognitive functions, are decreased in the cerebral cortex of AzD patients compared with those in healthy brains [5]. Therefore, one of the possible therapeutic approaches is to increase brain cholinergic levels by inhibiting acetylcholinesterase (AChE), the enzyme responsible for the hydrolytic degradation of acetylcholine. The amyloid hypothesis states that the increasing aggregation of *β*-amyloid (A*β*) is another element in the pathogenesis of AzD [6]. A*β* is a fragment of the amyloid precursor protein (APP) that results from proteolytic cleavage mediated by the enzyme beta-secretase 1 (BACE1). Consequently, dual inhibitors that block both AChE activity and BACE1-catalyzed A*β*-aggregation represent a promising therapeutic approach [7]. Dual-acting drugs have been approved for clinical use, such as donepezil-based and tacrine hybrids [8,9].

On the other hand, ligand-based rational drug design methods are widely used in drug discovery [10,11]; among them, quantitative structure–activity relationship (QSAR) is one of the methods with the greatest potential and opportunity for the development and screening of drug candidates for the treatment of AzD. Numerous studies have been developed using QSAR techniques for the design and screening of multi-targeting agents for the treatment of AzD. In 2014, Goyal and colleagues developed a robust and highly predictive group-based QSAR (GQSAR) model for combination library construction and identification of effective dual inhibition of BACE-1 and AChE [12]. The authors found some detailed interpretations of key fragments contributing to the activity. Recently, Dhamodharan and Mohan presented 2D QSAR models on multi-target ligands for predicting the dual inhibitory activity of *N*-benzyl piperidine derivatives [13]. Several machine learning (ML) techniques have been applied, including genetic function approximation (GFA) and nonlinear method, support vector machine (SVM), and artificial neural networks (ANN) [14,15]. However, the main drawbacks of these approaches are the poor interpretation of the applied techniques. In addition, the databases used for the development of QSAR models included only a small number of compounds and a low diversity of chemical structures, resulting in a small scope and a limited screening ability for the constructed models. Recently, we successfully developed powerful 0-3D QSAR models for predicting AChE inhibition using an expanded chemical library of 1975 bioactive compounds from the ChEMBL database and ML techniques [16]. Our newly designed compounds were also synthesized and showed good activities against the AChE enzyme [17,18].

Continuing our previous efforts to design and screen potential anti-AzD agents that inhibit both AChE and BACE1, this study was conducted to develop interpretable QSAR models for dual activity prediction using a large set of bioactivity data from the ChEMBL database and to utilize the chemical structure–activity relationships obtained from the classification trees for the design of novel dual targeting inhibitors. In this sense, we present a new design method based on a rule-of-thumb extracted from the established classification trees to develop dual inhibitors. We then discuss activity prediction and the future prospects of our new approaches.

## 2. Results and Discussion

### 2.1. Data Analysis and Activity Threshold Selection

**Figure 1:**
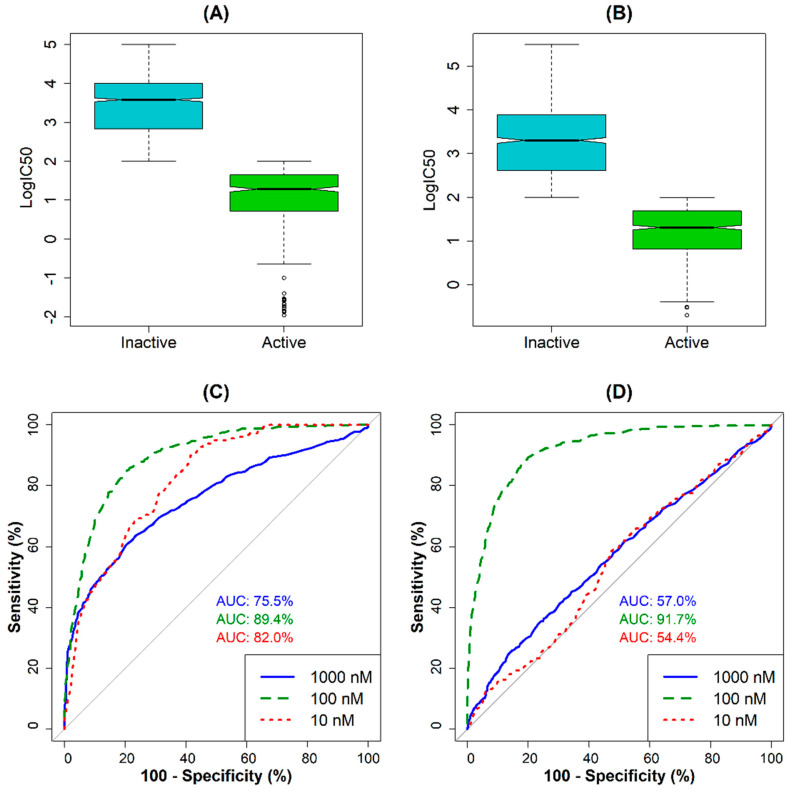
Distribution of the IC_50_ values for (**A**) active vs. inactive AChE inhibitors; (**B**) active vs. inactive BACE1 inhibitors; ROC−curve−based selection of cut-off values for (**C**) AChE inhibition and (**D**) BACE1 inhibition.

The random forest (RF) algorithm was used to determine the most appropriate cut-off value for classification. The results of the RF models for predicting AChE and BACE1 inhibition show that the area under the ROC curve (AUC) at 100 nM has the highest value for both models (Figure 1). According to these results, the compounds were classified as active if the IC_50_ value was <100 nM, otherwise they were inactive inhibitors. Following this procedure with a defined cut-off value, the data set for AChE was divided into 503 active and 1472 inactive compounds. In the case of the BACE1 data set, 617 active and 932 inactive compounds were identified (Tables S1 and S2). Figure 1A (for AChE inhibitors) and 1B (for BACE1 inhibitors) show the distribution of data for the compounds belonging to each class. Accordingly, the mean and median values of logIC_50_ calculated for AChE-active compounds were 1.04 and 1.28, respectively, whereas the corresponding values for the inactive compounds were 3.45 and 3.44, respectively. For the BACE1 inhibitors, mean and median IC_50_ values for the active compounds were 1.17 and 1.31, respectively, whereas the values for the inactive compounds were 3.28 and 3.30, respectively. After the cut-off values were selected, two data sets were divided into a training set and a test set. Using k-mean cluster analysis, the AChE data were grouped into seven clusters and the BACE-1 data into five clusters. By randomly selecting the most diverse structures from each cluster, we obtained the training set for AChE activity with 1385 data points (345 active and 1040 inactive compounds). Similarly, the training set of BACE1 activity includes 1085 (433 active and 652 inactive) compounds. It is noted that by using the chosen cut-off, an unbalanced distribution of the two classes is inevitable, which may affect the performance of the classifiers [19]. However, we found during the modeling process that this effect was not significant, so we did not apply a rebalancing strategy to the final data [19].

### 2.2. Machine Learning Models

Table 1 summarizes the performance parameters of all tree-based ML models in both data sets. The results of the training set were calculated based on the results of the 10-fold CV procedures. For the AChE data set, the overall accuracy of the best model matched the AChE-RF obtained using RF algorithm. The global accuracy (Q^2^) for training and testing validation of the best model were 0.87 and 0.86, respectively. It is not surprising that a multiclassification system such as AChE-RF was able to outperform the two individual models obtained using the general classification and regression tree (CART) and chi-square automatic interaction detection (CHAID) algorithms [20]. However, it should be noted that these two stand-alone models performed acceptably compared with AChE-RF. Moreover, the effect of unbalanced data on the performance of all three models can be observed. Whereas the accuracy and sensitivity of the models were generally quite high (>0.8), the precision values were still low, resulting in a low F1 performance (<0.77). This effect was not observed in the ROC analysis. As can be seen in Table 1, the AUC values for the test sets were almost identical to the global accuracy. Compared with the AChE data, the BACE1 data are less unbalanced with a prevalence ratio of 1.5 in favor of the inactive class. The difference between the values for precision and sensitivity was not very large. We can note that the global accuracy of the BACE1-RF model was also higher compared with the other classifiers. In general, all the models obtained here showed good performance, especially the predictability on the external test set, which is important for the screening of active compounds against each target.

The applicability domain (AD) for each ML model was determined using the distance-based method (see Table 2). If a new chemical is to be predicted, it must fall within the AD of the model. Accordingly, some compounds in the test set were identified as structural outliers.

### 2.3. Identification of AChE and BACE1 Inhibitory Activity Rules

The decision trees are combinations of rules describing the relationships between molecular descriptors [21]. Since the rules are the basis of activity predictions, we define the chemical space of active inhibitors following the rule paths that lead to the highest classification accuracy of the positive class. As the RF models are ensemble decisions trees, these activity rules were mainly based on CART and CHAID models. These activity rules are of great interest because the ligand-based design approaches implemented in this study are based on the structural fragments of the active compounds that accomplished the rules and the predictions of all the constructed ML models.

**Figure 2:**
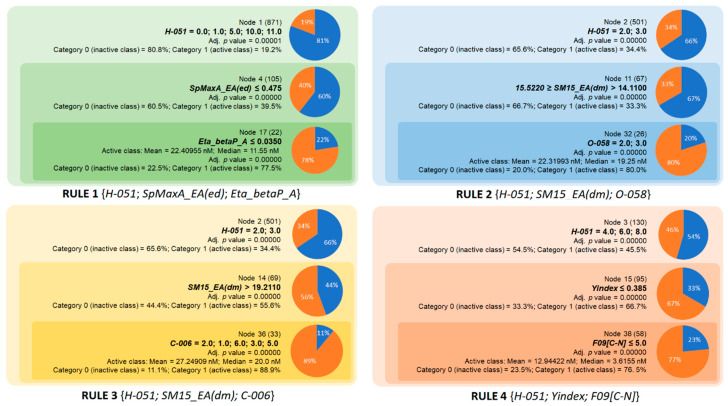
Overview of the four chemical rules elaborated in the current study and applied to predict AChE inhibition.

According to the rules for AChE inhibition, we identified four rules based on eight molecular descriptors (see Table 3). The first rule consists of three descriptors, including *H-051*, *SpMaxA_EA(ed)*, and *Eta_betaP_A* (Figure 2). The descriptor *H-051* represents the number of fragments consisting of H bound to alpha C and makes a positive contribution to the logP value and molar refractivity (MR) of active compounds according to the Ghose–Crippen method [22]. Note that alpha C can be defined as C bonded to any electronegative atom such as O, N, S, P, and halogens [23]. At the first stage, *H-051* values of 0, 1, 5, 10, and 11 identified 19.2% of the active compounds. This percentage increased significantly to 39.5% when an upper limit of *SpMaxA_EA(ed)* was applied. This is a topological index derived from the edge adjacency matrix and related to molar volume [24]. Applying another limit to the descriptor *Eta_betaP_A* (≤0.035), Rule 1 achieved 77.5% accuracy in classifying the active compounds. The descriptor *Eta_betaP_A* is derived from the valence electron mobile (VEM) environment theory [25] and represents the total contribution of pi bonds and lone pairs of all the atoms in the molecule. Based on the descriptors selected in Rule 1, a small chemical space for AChE inhibitors was determined. In terms of the chemical properties exhibited by these descriptors, aromatic rings and lipophilicity were identified as important factors contributing to activity. AChE Rule 2 was identified by applying the lower limit of the edge adjacency index *SM15_EA(dm)* in combination with the same number of two atom-centered fragments *C-006* and *O-058* [23]. The descriptor *O-058* is the number of =O fragments that make a negative contribution to the logP value. In addition, AChE Rule 2 showed better accuracy than Rule 1 (80.0%). Similar to Rule 2, AChE Rule 3 consisted of three descriptors, including *H-051*, *SM15_EA(dm)*, and *C-006*. This rule also showed the highest accuracy compared with the other AChE rules. In contrast to *H-051*, the fragment *C-006*, defined as CH_2_RX, has a negative contribution to the logP value and a positive contribution to MR parameters according to the Ghose–Crippen model. Since the logP value is mainly associated with lipophilicity and MR with the mean polarizability of a molecule, Rule 3 emphasizes the importance of a balance between the lipophilicity and polarizability in AChE inhibitors. Rule 4 is composed of *H-051*, *Yindex*, and *F09[C-N]* and has an accuracy of 76.5%. It is worth noting that *Yindex* and *F09[C-N]* are mainly related to the size of the molecules. The Balaban-like information *Yindex* is defined as the vertex distance degree of the topological distance matrix of molecules [26], and *F09[C-N]* simply represents the number of topological distance (order 9) between C-N atom pairs. As can be seen from Rule 4, AChE inhibitors in this chemical space can have 4-8 *H-051* fragments but the lipophilicity and molecular size should be limited, with *Yindex* and *F09[C-N]* not exceeding 0.385 and 5, respectively. Another interesting point is the activity space, which corresponds to the chemical space defined by all the rules. Comparing the mean and the median IC_50_ values of AChE inhibitors in four rules, the ranking is as follows: Rule 4 < Rule 1 < Rule 2 < Rule 3 (Figure 2). A total of 139 compounds falls under these rules, with the median and mean IC_50_ values for compounds in Rule 4 being 12.9 and 3.6 nM, respectively. Applying the four rules, 70 compounds with IC_50_ < 7.79 nM (median value) were identified, indicating that these rules are suitable for screening very potent AChE inhibitors whose structural features can be used to design new inhibitors of this enzyme.

**Figure 3:**
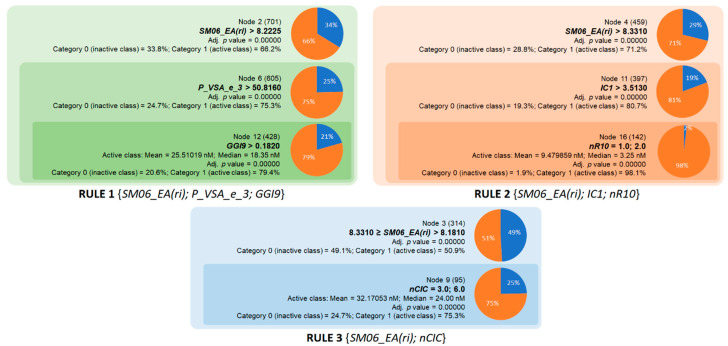
Overview of the three chemical rules elaborated in the current study and applied to predict BACE1 inhibition.

The second group comprises three rules for the inhibition of BACE1 (Figure 3). These rules were defined based on the six descriptors listed in Table 4. Rule 1 was the combination of three descriptors *SM06_EA(ri)*, *P_VSA_e_3*, and *GGI9*, with a classification accuracy of 79.4%. The descriptor *SM06_EA(ri)* is a theoretical feature and is calculated by the sum of the diagonal elements of the sixth power of the atomic adjacent matrix weighted by resonance integral, which in turn is a measure of the strength of intramolecular binding interaction [23]. *P_VSA_e_3* is calculated by summing the van der Waals surface area (VSA) over the atoms with a Sanderson electronegativity within a range of 3 [27]. This descriptor is likely related to the shape and size of the inhibitors. *GGI9* is based on the total charge transfer between atoms located at topological distance 9 [23]. In this context, docking simulations demonstrated the role of pH value and charge state of the ligand in the interaction with the aspartic dyad (Asp32/Asp228), the key residue in the active site of BACE1 [28]. Rule 2, which has a very high accuracy of 98.1%, was created by combining *SM06_EA(ri)* and two interpretable descriptors, *nR10* and *IC1* (Figure 3) [23]. It is worth mentioning that using a lower limit of 8.331 for *SM06_EA(ri)*, the one-property rule is able to identify >71% of BACE1 inhibitors from 459 compounds in the database. This rule should be considered a good starting point for further exploration of the structure–activity relationships of BACE1 inhibitors. By applying the lower limit for *IC1* as 3.513, the accuracy of this rule increased significantly to >80%. The descriptor *IC1* is simply defined as the probability of encountering an order 1 carbon atom over the total carbons of the molecules, represented as a multigraph filled with hydrogen [23]. Rule 2 was filled in by adding a cyclicity index, namely *nR10*. Accordingly, 98% of the compounds with *SM06_EA(ri)* > 8.331 and *IC1* > 3.513 together with one or two 10-membered rings could be classified as active inhibitors. Rule 3, which simply combined a range of *SM06_EA(ri)* [8.181; 8.331] together with the cyclomatic number *nCIC* equal to 3 or 6, achieved a good prediction accuracy of >75%. Moreover, BACE1 inhibitors identified by Rule 2 had mean and median IC_50_ values of 9.48 and 3.25 nM, respectively (Figure 3), showing greater potential compared with Rules 1 and 3. Overall, three rules for BACE1 inhibition covered a large chemical space encompassing the structures of 617 active compounds and had very good performance in screening potential inhibitors, especially Rule 2.

### 2.4. Reposition of Dual-Targeted Inhibitors

The developed rules and QSAR models could be considered as useful tools for screening potential dual inhibitors against AChE and BACE1 simultaneously. The original curated database, which included 3500 compounds, was mainly screened for inhibitory activity against a single experimental target such as AChE or BACE1. In this context, the AChE rules and models were applied to screen AChE inhibitors from the BACE1 database and vice versa. The activity predictions were then validated by molecular docking simulations.

First, three BACE1 rules were applied to characterize 503 active compounds from 1975 compounds in the AChE database. As a result, 30 compounds were accomplished with Rule 1, 3 compounds with Rule 2, and 19 compounds with Rule 3. By applying three QSAR models, 32 compounds were predicted to be active BACE1 inhibitors with experimental AChE inhibition. Of 630 active inhibitors with IC_50_ ≤ 100 nM against the BACE1 enzyme, 0 compounds matched Rule 1, 13 compounds matched Rule 2, 18 compounds matched Rule 3, and 1 compound matched Rule 4. Based on QSAR predictions, only 2 compounds were predicted to be dual inhibitors.

**Figure 4:**
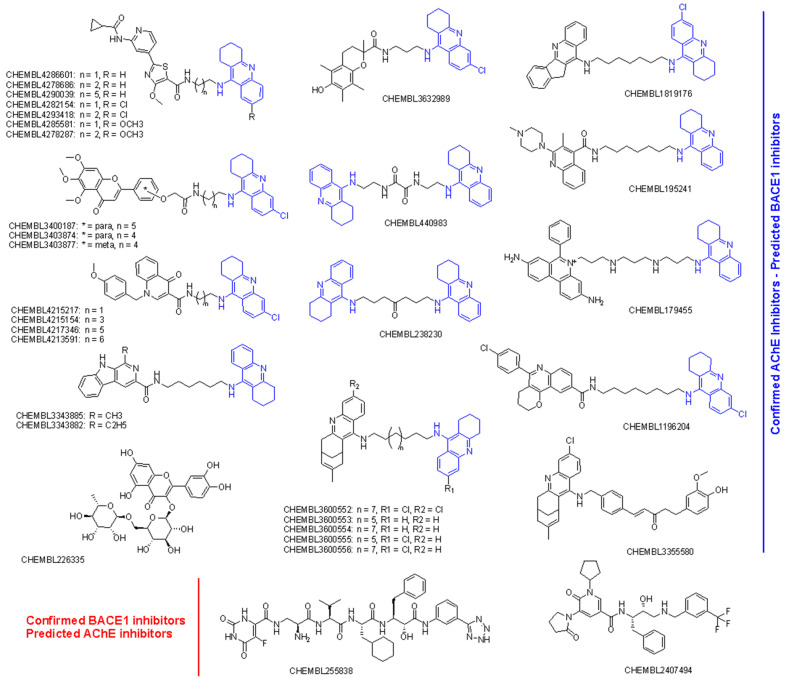
Chemical structures of AChE and BACE1 dual inhibitors screened by activity rules. The large number of tacrine derivatives are highlighted in blue.

A total of 34 compounds were predicted to be active dual inhibitors against AChE and BACE1. The prediction results and experimental measurements are summarized in Table 5, and the chemical structures of these 34 compounds are shown in Figure 4. Most of the confirmed AChE and predicted BACE1 inhibitors were tacrine derivatives. Only two compounds were not tacrine analogues, including a shogaol–huprine hybrid (CHEMBL3355580) and rutin (CHEMBL226335), a natural flavonoid glycoside from citrus. Whereas rutin has been shown to be able to impair BACE1 cleavage by acting as a *β*APP-selective BACE1 inhibitor [29], huprine-based hybrids have shown promise as AChE inhibitors with potential inhibitory effects against both *β*-amyloid peptide (*β*A) and tau aggregation [30]. In fact, five compounds with huprin–tacrin hybrid structures were predicted to be AChE and BACE1 dual inhibitors. Interestingly, these inhibitors were experimentally shown to act as multitargets, with inhibitory effects on AChE and AChE-induced A*β*1−40 and PrP106−126 aggregation, BChE, self-induced A*β*1−42 aggregation, and BACE1 [31].

**Figure 5:**
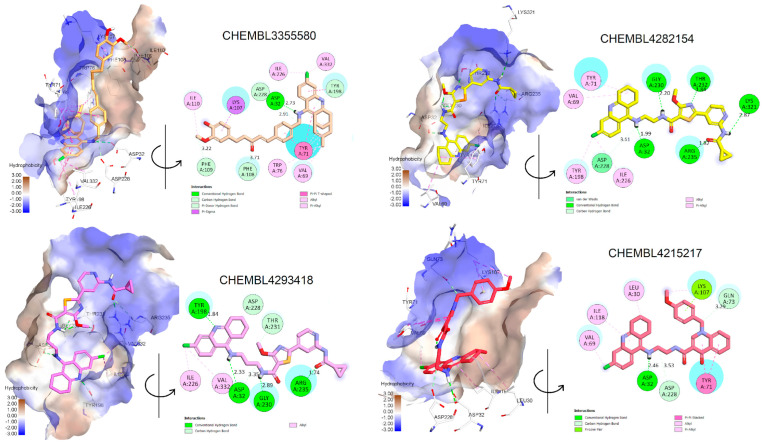
Two- and three-dimensional docking conformations of some exemplary dual inhibitors estimated by experimental and rule−based approaches in the active site of BACE1.

The docking results of these compounds showed their good binding interactions with BACE1 (PDB ID: 2WJO) [45]; however, most of the binding energies estimated by docking scores (dG) are lower than that of the co-crystal ligand, which had a value of −15.23 kCal/mol. This value was determined after the native ligand, namely QUD, a catalytic inhibitor (2-(2-amino-6-phenoxy-4*H*-quinazolin-3-yl)-2-cyclohexyl-ethyl)-amide, was redocked into the active site of BACE1 as a reference compound (r.m.s.d = 0.6678 Å). The docking score applied is based on GBSA/MM function, which combines numerous weighted energy terms, including van der Waals, H-bonding, and the hydrophobic, solvation, and desolvation energy changes [46]. Based on dG values, only four compounds exhibited better binding energy than QUD; these were CHEMBL4282154, CHEMBL4293418, CHEMBL3355580, and CHEMBL4215217 [30,39,42]. Notably, the first two compounds were designed by combining 6-Cl-tacrine with a pyridine carboxamide moiety, a promising fragment found in glycogen synthase kinase-3*β* (GSK-3*β*) inhibitors (Figure 4) [39]. According to the docking interactions (Figure 5), all the compounds were able to bind strictly to the active site of BACE1, with multiple H-bonds and stacking interactions from the 6-Cl-tacrine and huprin moieties to Val69, Tyr71, Ile226, Val332, and Tyr198. It is worth highlighting that all the compounds consistently participate in two H-bonds with Asp32 and Asp228 (Asp dyad), which are among the key interactions of the catalytic mechanism of BACE1 (Figure 5).

**Figure 6:**
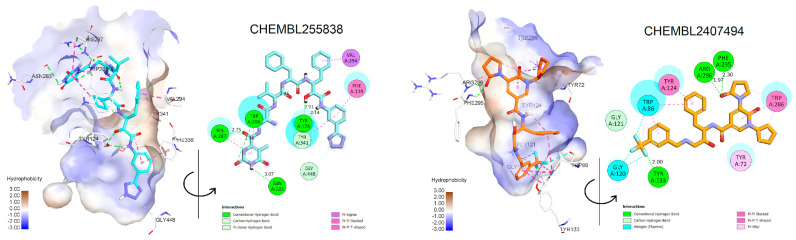
Two- and three-dimensional docking conformations of two exemplary dual inhibitors estimated by experimental and rule-based approaches in the active site of AChE.

In contrast to the screening of BACE1 inhibitors, the screening of AChE inhibitors using activity rules and QSAR models revealed only two active compounds, CHEMBL255838, a polypeptide with a 5-fluoroorotyl group [43], and CHEMBL2407494, a hydroxyethylamine-based inhibitor with a pyridone ring [44]. They were predicted to be AChE inhibitors and had IC_50_ values <100 nM against the enzyme BACE1 (Table 5). Docking simulations revealed that they were able to bind to the active site of the AChE enzyme with similar or even better binding energies than the reference drug donepezil (dG = −14.35 kCal/mol, r.m.s.d = 0.389 Å) [18]. Both CHEMBL255838 and CHEMBL2407494 were able to interact with residues in the peripheral anionic site (PAS) of AChE, such as Tyr72, Tyr341, and Trp286, and those in the choline binding site, such as Trp86 and Gly448 (Figure 6). Moreover, these compounds were able to form stacking interactions with Phe295 and Phe338 at the acyl binding pocket, which are important for selective inhibition of AChE. The active site of BACE1 (beta-secretase 1) is relatively large and deep [47], whereas the active site of AChE is shorter and narrower [48]. Although the active sites of AChE and BACE1 have different structures and residues, both enzymes are homologous in their catalytic mechanisms. The serine residue in AChE and the aspartic acid residue in BACE1 both act as nucleophiles that attack the substrate to form a tetrahedral intermediate that eventually collapses and releases the products. The role of the inhibitors is to interact with the active site and prevent the substrates from reaching the active site. The obtained results confirmed the inhibition of the AChE enzyme by CHEMBL255838 and CHEMBL2407494.

### 2.5. Design of Novel Dual-Targeted Inhibitors

In this study, we proposed a fragment-based drug design method that can be used for the development of novel chemical entities acting as dual-targeted inhibitors of AChE and BACE1 enzymes. This method consists of two steps: (i) identification of the most active fragments based on the chemical space defined by the chemical rules established for each activity and (ii) assembly of the most active fragments to obtain novel dual-targeted inhibitors.

**Figure 7:**
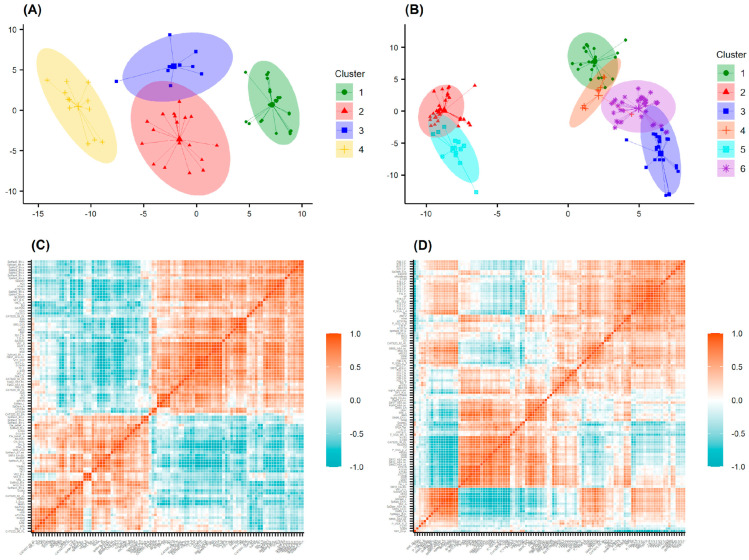
Results from the clustering using the *k*−MCA algorithm. (**A**) The 67 representatives of the AChE database; (**B**) the 114 representatives of the BACE1 database. Correlation matrix of clustering features computed for (**C**) 67 AChE inhibitors and (**D**) 114 BACE1 inhibitors.

First, the rules were applied to filter the data set from which we selected 67 active cases for the AChE and 144 cases for BACE1 data sets (Tables S5 and S6). To characterize the chemical profiles of these data sets, cluster analysis was performed using the *k*-MCA algorithm. As a result, the data from 67 AChE inhibitors were divided into four clusters using 100 variables (Figure 7A,C). The results also indicated that there are significant differences between the clusters in both the chemical profiles (*p* < 0.01) and inhibitory activities of the selected inhibitors. Among the four clusters, the mean and median values of cluster 2 were the lowest at 1.07 and 0.53 nM, respectively. This was followed by cluster 1, with mean/median values of 8.59/3.46 nM, cluster 4 (19.35/5.01 nM), and cluster 3 (22.53/6.96 nM).

According to the 144 BACE1 inhibitors, the data were divided into six clusters based on 110 variables selected by analysis of variance (Figure 7B,D). The ranking along with the mean/median values calculated for all the clusters were as follows: cluster 3 (1.54/0.58 nM), cluster 1 (3.78/0.4 nM), cluster 2 (9.55/2.0 nM), cluster 6 (9.33/4.0 nM), cluster 5 (11.95/6.7 nM), and cluster 4 (21.21/9.5 nM). The compounds from each cluster were then predicted by the AChE or BACE1 models, and only those that were actively predicted by all three models for each activity were selected for the further stages of drug design.

**Figure 8:**
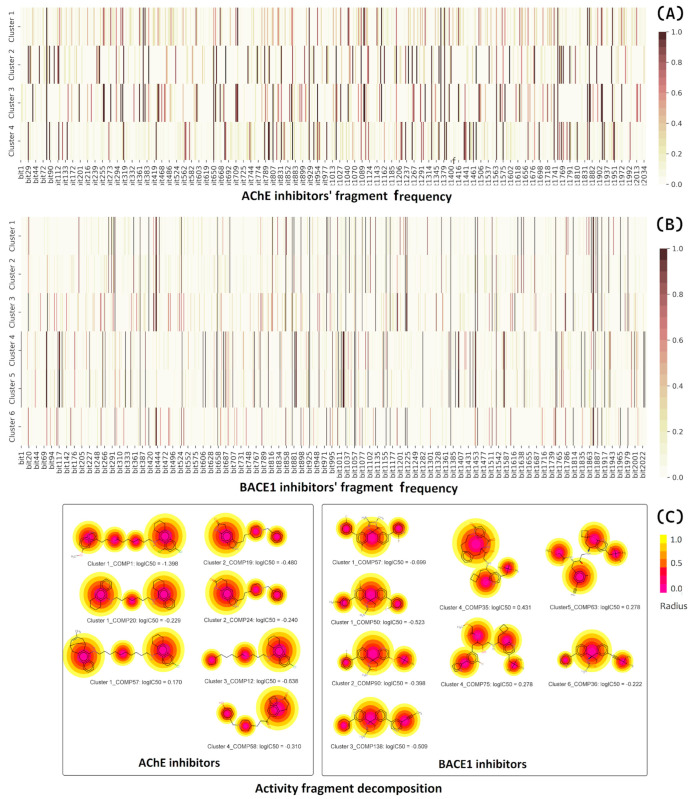
Fingerprint frequency among (**A**) AChE and (**B**) BACE inhibitors in each cluster; (**C**) fragment visualization of the most active inhibitors by Murcko−type decomposition algorithm [50].

In the next step, the chemical space of compounds in each cluster was represented by the more than 4000 bitsized ECFP4 fingerprint of Murcko scaffolds. The active fragments were defined as those whose abundance in each cluster was >0.5. For the AChE data 58, 67, 89, and 70 fragments were determined for clusters 1, 2, 3, and 4, respectively, (Figure 8A). The correlations of fragment frequencies of all four clusters were very low, indicating the chemical diversity of each cluster. It is noteworthy that the Murcko fingerprints are defined based on the graph framework centered on the ring systems and linker atoms connecting the ring systems. The decomposition radius should not be very low because the basic graph path might not represent the active fragments, as the inhibitory activity seems to be mainly determined by the larger scaffolds such as tacrine, benzisoxazole, *N*-benzylpiperidine, and pyrrolidinone as well as other complex heterocyclic systems [49]. We then selected those Murcko fingerprints with radii greater than 0.5 for designing new inhibitors. Figure 8C shows some of the most active AChE inhibitors of each cluster, along with the major scaffolds determined by Murcko-type decomposition algorithm implemented in RDKit (https://www.rdkit.org/). A total of 192 active fragments of AChE inhibitors were selected for assembly.

Following the same steps described above, we identified the most frequently counted scaffolds for six clusters (1–6) of the BACE1 data set, comprising 69, 59, 74, 86, 108, and 61 bits, respectively. After selecting the highest reassembly radius, the final number of fragments to be used in the next design tasks was 240.

**Figure 9:**
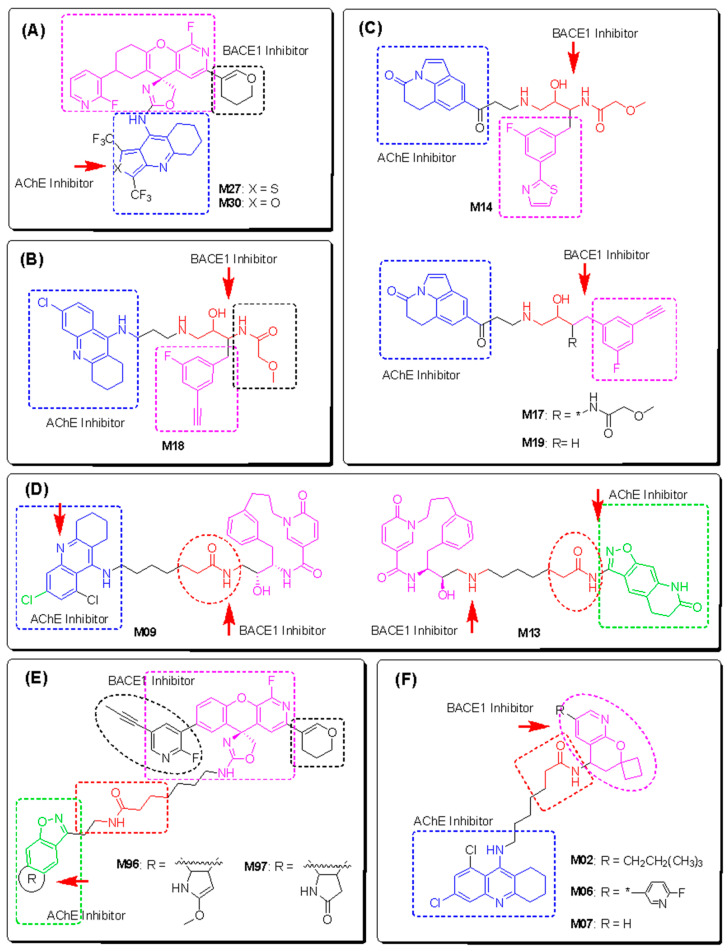
Structural design of novel AChE and BACE1 dual inhibitors. 13 designed compounds are sorted in 6 groups (**A**–**F**) according to their structural similarity. The active fragments are highlighted and colored according to their targets. The * indicates in each structure where that substituent group attaches to the designed molecule.

The final key fragments of each property were used to develop novel dual inhibitors. These compounds were created by assembling fragments thought to be active on each target, which is shown by dashed lines (Figure 9). The newly designed compounds were then predicted by all six QSAR models, including three for AChE and three for BACE1 inhibition. Only those estimated to be active inhibitors of both AChE and BACE1 enzymes were selected for further analysis.

**Figure 10:**
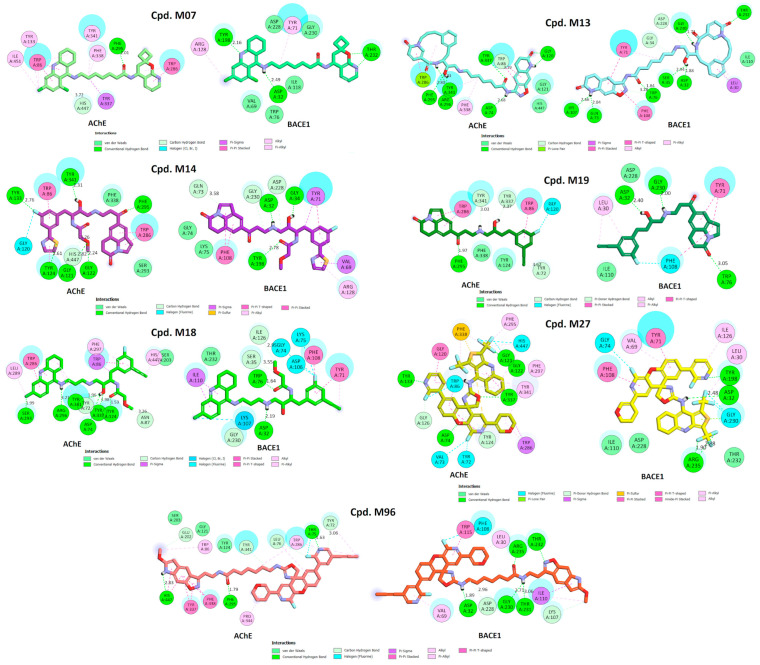
Two-dimensional interactions of designed compounds with residues of the active sites of AChE and BACE1.

To expand the structural diversity of novel dual-target AChE/BACE1 inhibitors, we added and/or replaced various substituents to/from the main scaffold or replaced the entire core with equivalent surrogates based on the theory of bioisosterism. For example, we replaced the benzene ring with a thiophene or a furan ring. The chlorine substituent was exchanged for an electron-donating group such as a methoxy substituent (-OCH_3_) or an even stronger electron-withdrawing group such as a trifluoromethyl group (-CF_3_) among others. As a result, 250 new compounds were manually designed and then predicted using the developed ML models to estimate their activity. Before the predictions, all compounds were evaluated by the applicability domain of all the models. Only 73 compounds were identified that met the AD of the six models. Of these, 13 compounds were predicted as active inhibitors by both the AChE and BACE1 consensus models. They were then validated by docking simulations. The results are shown in Table 6 and Figure 10.

As shown in Figure 9, the first group (A) includes two hybrids of aminooxazoline xanthene and cyclic ether containing quinoline (M27 and M30). M27 was predicted to bind more strictly to both targets compared with M30. A recent study has shown that nitrogen-, oxygen-, and sulfur-containing heterocyclic scaffolds significantly enhance the inhibitory effects of AChE and BChE [51]. In this context, the aromatic system of the quinoline moiety penetrates deeply into the AChE pocket and contributes to the stacking interactions with the acyl and choline binding site, whereas the secondary amine and halogen groups interact well with the Asp dyad of the BACE1 enzyme. The aminooxazoline 3-aza-xanthene (3-aza-AOX) core plays an important role in the interaction with the PAS region in AChE and the mobile flap region in BACE1, which is important to keep the molecule stable inside the pocket [18,52].

The tacrine derivative M18 and the tricyclic isatins M14, M17, and M19 of group (B) showed good activities according to rule-based QSAR models and docking interactions. The cores of tacrine and 5,6-dihydro-4*H*-pyrrolo[3,2,1-*ij*]quinoline-1,2-dione were able to form multiple stacking interactions with acyl pocket and PAS regions in AChE and the flap domain from Pro70 to Trp76 in BACE1. Importantly, the hydroxyethylamine-based linker is responsible of two H-bonds towards Asp32 and Asp228 of the Asp dyad of BACE1 [44]. With the incorporating 2-(3-fluorophenyl)thiazole on the other side, M14 showed a higher H-bonding interaction ability with residues in the catalytic triad and oxyanion hole of AChE, resulting in better binding energy toward the target. In addition, the α-methoxypropanamide moiety in M14 and M18 might play a role in the interactions with the PAS region in AchE, as well as hydrophobic and amphipathic residues at a subsite closed to the C-terminal lobe of BACE1, such as Ser35, Val69, Tyr71, Ile126, and Tyr198.

The 6,8-dichlorotacrine derivatives were further explored by incorporating other active BACE1-inhibitory scaffolds such as 2-pyridyl-substituted pyridonyl dienes in M02, M06, and M07 (Figure 9F). Compound M07 proved to be the most active dual-enzyme inhibitor among these derivatives. The halogen group in M07 was able to interact with several residues at the bottom of AChE pocket, including those of the catalytic triad and the choline binding site. Its binding energy was similar to that of donepezil and was −14.84 kCal/mol. The pyridonyl dienes interacted with Trp286 of PAS in AChE but showed no interactions with the catalytic centers of BACE1. In contrast, the secondary amine in the linker interacted well with Asp32 and Asp228 of the Asp dyad and with Tyr71 in the flap domain of BACE1.

Macrocyclization has been shown to improve the potency of BACE1 inhibitors [44,53]. In this study, this core structure was combined with the AChE inhibitor fragments in group (D) to generate dual inhibitors. Among them, compounds M09 and M13 were predicted to be active compounds based on the QSAR models and docking simulation. As shown in Figure 10, the alkyl-linked 15-membered macrocycle is responsible for the interactions with the different domains in AChE and the catalytic dyad of BACE1. The incorporation of a pyridone moiety into the macrocycle significantly improved the stacking linkage to Trp286 (AChE PAS) and H-bonding to Thr232 (BACE1). One the other hand, the dichlorotacrine- or isoxazole-containing tricycles are important scaffolds for interaction with the choline binding site of AChE and the flap domain of BACE1 [54,55]. The long aliphatic chain also plays a role while the inhibitors insert into the hydrophobic cavity of the respective enzymes.

Considering the importance of isoxazole-containing tricycles in AChE binding, this moiety was combined with a cyclic ether containing a quinoline of group (A) to obtain several novel inhibitors of group (E). Among them, M96 and M97 showed the strongest inhibition of the two enzymes. The long and highly flexible structures of the compounds of this group allow them to interact with all the catalytic cavities of AChE and BACE1. Compound M96 displayed better binding affinity, with the lowest docking scores compared with the other derivatives and the two reference docking compounds donepezil and QUD. The results once again confirmed the importance of a long aliphatic chain linking two active scaffolds with inhibition of two targets, especially for interaction with residues in the PAS region of AChE and catalytic Asp dyad of BACE1 (Figure 10) [56].

### 2.6. Comparison with Previous Studies

As is well known, rational multi-target drug design has emerged in recent years as an attractive paradigm for drug discovery, offering potential therapeutic solutions for AzD [49]. Computational tools such as QSAR models, activity rules, and molecular docking have long been successfully used in the early stages of drug design and virtual screening of bioactive compounds [21]. Classically, most of the published QSAR work focused on only one targeting strategy, e.g., AChE, butyrylcholinesterase (BuChE), monoamine oxidase (MAO), or BACE1. In our literature search, we found 18 studies applying QSAR approaches to AChE inhibitor development and another 11 papers on BACE1 inhibitors (Tables S7 and S8).

Specifically, from 2007 to 2021, dozens of models were developed to predict the inhibitory effects of AChE based on a variety of chemical structures, including pyrimidines, porphins, pentenones, and flavonoid derivatives among others. Both 2D and 3D descriptors were used, and various ML techniques were employed to develop QSAR models. However, most published models were mainly based on homogeneous and small databases (mostly <100 compounds), so their scope seemed to be limited. Moreover, training and validation results were variable, especially for regression models, with coefficients of determination (R^2^) ranging from 0.6 to 0.9. This was similar to QSAR models for BACE1 inhibitors, although the number of publications in this area was somewhat smaller.

To allow direct comparison with the same studies already reported in the context of multi-targeting anti-AzD drug development, we filtered out only 2D-QSAR methods, including regression and classification models applied to dual AChE and BACE1 inhibitors. As a result, four studies were found, and their main findings are summarized in Table 7. Of these, three were published after 2020, highlighting the current trend toward multi-target drug design to combat AzD. In general, all published models showed reasonable performance.

Using regression models, Goyal et al. developed a global model, namely GQSAR, which included only three variables (delta epsilon A, nitrogen count, and K3 alpha) and showed good performance in predicting BACE1 activity [12]. AChE activity was then estimated using the same model in combination with docking simulations. However, the training group included only 20 dihydropyridine derivatives, which might limit the predictive ability of the developed models.

On the other hand, the study by Tran et al. employed various methods, including 2D-QSAR, pharmacophore, and molecular docking, for the rational design of several curcumin and flavonoid derivatives with dual AChE and BABE1 inhibitory activity [57]. Although the amount of data was still limited, the authors demonstrated their predictive ability by synthesizing two inhibitors (F9 and F24) that clearly showed good activity against two targets, indicating the general validity of QSAR tools.

By using various ML techniques, Dhamodharan and Mohan were able to develop several regression models that showed good performance [13]. According to the classification models, Stern et al. developed numerous models based on a large chemical collection from the ChEMBL database [14]. Their models showed regular performance, and screening tests revealed six active compounds.

Compared with the previous studies, the models developed in this work showed higher performance in an updated and larger data set. By integrating different approaches into the design protocol, we also rescreened the ChEMBL database to identify dual AChE and BACE1 inhibitors. In addition, no rule-based approaches have been explored to date. Based on the developed activity rules, we identified the active fragments and assembled them into novel structures that were most likely to act as dual inhibitors. Several of them showed very good binding affinity to AChE and BACE1 targets in docking simulations. The comparison revealed that the rule-based and classification models developed in the current work for the design of novel AChE and BACE1 inhibitors can overcome the main drawbacks of previously published models, which are related to the limited training data set and the low interpretability of ML algorithms.

Despite the mentioned advantages, the current computational approaches still have their own limitations. Since the activity rules only focused on the chemical space in which the QSAR models showed the highest accuracy, the screening spaces were likely to be narrowed. Consequently, the newly screened or designed structures are not “really new”. As a solution, future studies should adopt more activity rules by reducing the accuracy cut-off to an acceptably lower level, e.g., >0.65. We have previously discussed the potential application of a multiclassification system for improving decision making by QSAR models [15]. Several approaches can be used, such as voting, support function fusion, bagging, boosting, and stacking. In addition, the main drawback of docking simulations is the low correlation between the docking scores and binding energies [52]. Meanwhile, the docking assays were only used in this study for simulating the binding modes of newly designed inhibitors. To estimate the free binding energies of these compounds, it is desirable to perform more sophisticated techniques such as QM/MM-GBSA, MM-PBSA, or MM-GBSA [58]. Lastly, experimental evaluations using AChE and BACE1 enzyme assays, even though they were out of the scope of this study, would be useful for additional confirmation of the computational predictions.

In this sense, in silico methods are widely used in pharmaceutical fields, and in many cases, computerized assessments in both preclinical and clinical phases are adopted by the respective regulatory authorities [59]. To this end, the current modeling approaches were developed according to the general validation principles for QSAR models established by the OECD (Organization for Economic Co-operation and Development) [60]. Therefore, the models developed in this study are suitable to support wet-lab experiments in future work.

Nevertheless, there are also limitations in the present study such as the risk of ADMET issues (absorption, distribution, metabolism, excretion, and toxicity). When using predictive tools to estimate these ADMET parameters of compounds, it is important to consider the structure and molecular weight of the compound in question. Compounds with a large and complex structure and high molecular weight may not lend themselves to accurate predictions using these tools. Therefore, it is necessary to continue research after proposing the structure of the compound by conducting further studies to optimize it for the best possible ADMET profile. This may mean modifying the structure or properties of the compound by various methods such as synthesis or formulation, followed by testing the ADMET profile by various assays and experiments to ensure the success of the optimization. Therefore, the first step is to propose the best possible structure of the compound, as was performed in the present study. This will be followed by further studies to improve and obtain the best possible ADMET profile to pave the way for future in vivo studies.

In addition, it must be taken into account that, in general, small molecules often have an effect on multiple targets depending on their binding affinity. These targets may or may not be related to AChE or BACE1 and may have unintended effects beyond anti-Alzheimer’s activity. Various methods, such as proteomics-based research using protein interaction networks and similarity studies with known agents, could help identify such targets.

## 3. Materials and Methods

### 3.1. Data Curation and Labeling

A large bioactivity library of 28,304 AChE inhibitors and 14,457 BACE1 inhibitors was extracted from the ChEMBL database (https://www.ebi.ac.uk/chembl/ accessed on 10 April 2022). In this way, compounds were selected whose test organism category was *Homo sapiens* and whose target was a single protein. The half-maximal inhibitory concentration (IC_50_) values were converted to nanomolar (nM) units. In the next step, the data were cleaned by removing duplicate structures, compounds with unclear inhibitory activity (inconclusive IC_50_ values), and salts. In the ChEMBL data validity section, compounds that were “outside typical range” or “Potential transcription error” were removed. After data curation, we obtained a homogenized library of 1975 AChE inhibitors with different chemical functional groups, including indoles, acridines, catechins, quinolines, and others. The same procedure was performed for the extracted BACE1 data set. A total of 1549 compounds with different structural cores were found (Tables S1 and S2).

In order to assign discriminatory labels to AChE or BACE1 activities, such as active and inactive inhibitors, an appropriate threshold was chosen for the response variable. In this regard, the selected cut-off value should meet the following criteria: (i) it must be small enough to distinguish active from inactive inhibitors and (ii) it must be at a balance-dividing point because the problem of biased data could negatively affect the model performance. Then, three levels of IC_50_ values (10, 100, and 1000 nM) were considered. The susceptibility of each cut-off value was estimated based on the machine learning RF approach, comparing the model performance of 200 trees with ~100 molecular features. The main criteria used for comparison included the receiver operating characteristic (ROC) curves, data variance, and area under the ROC curve (AUC).

### 3.2. Descriptor Calculation and Training Set Selection

Dragon software (academic version 6.0) was used to calculate molecular descriptors for the two data sets using the simplified molecular-input line-entry system (SMILES) codes [23]. Excluding missing values and those with zero variance, a total number of 1100 and 1151 0-2D descriptors were calculated for the AChE and BACE1 data sets, respectively.

In the next step, the data were rationally divided into training and test sets based on *k*-means cluster analysis (*k*-MCA). The number of variables included in the k-MCA and the number of clusters were optimized considering Fisher’s ratio and significance level (*p* < 0.05). The AChE data set was divided into seven clusters with 1385 cases in the training set (approximately 70%) and the remaining 590 cases in the test set. For the BACE1 data set, five clusters were identified and then the data set was divided into training and test sets with 1085 and 464 compounds, respectively.

### 3.3. Development and Validation of Machine Learning Models

To capture the non-linear structure–activity relationships and to improve the interpretability of the QSAR models, several decision trees learning algorithms were employed [61], including general classification and regression tree (CART), chi-square automatic interaction detection (CHAID), and random forest (RF) [21]. The configurations of each model are described below:

- CART tree was constructed using the impurity Gini-based binary classification tree algorithm implemented in IBM SPSS Statistics v.22.0 [61]. For the AChE-CART model, a maximum tree depth of 9 and a minimum number of 150 cases in the parent node and 40 in the child node were configured. In the case of BACE1-CART, the model was built with the following parameters: maximum tree depth of 5 and a minimum of 100 cases in the parent node and 20 cases in the child node. For both models, the tree pruning technique was used to avoid overfitting and the prior probability in all categories was reset to the same value before running the models.
- The CHAID model was created by splitting data into mutually exclusive and exhaustive subsets with the best description of the dependent variable. A major difference between the CART and CHAID algorithm is that CART produces binary splits that imply two possible outcomes, whereas CHAID can generate multiple branches of a single root/parent node [61]. The parameters of AChE-CHAID models were set as follows: maximum tree depth: 3, minimum number of cases in parent node: 60, minimum number of cases in child node: 18. For the BACE1-CHAID model, the following parameters were set as follows: maximum tree depth: 3, minimum number of cases in parent node: 100, minimum number of cases in child node: 45.
- RF is a multiple classifiers system (MCS) consisting of a collection of tree-structured classifiers. Significant improvements in classification accuracy have been achieved by growing an ensemble of trees and having them vote for the most popular class [62]. In this work, the RF models were built based on variables selected from the CART and CHAID algorithms. The RF algorithm implemented in Statistica 11.0 was used. The importance of selected variables was evaluated by a normalized importance scale. As a result, 172 and 117 variables were selected for the AChE-RF and BACE1-RF models, respectively. The hyper-parameters set for the AChE-RF model included a size of 200 trees, a number of predictors of 8, a subsample proportion of 0.5, a maximum number of levels of 5, a minimum number of cases of 50, a maximum number of nodes of 100, and a minimum number of subordinate nodes of 10. The BACE1-RF model had the following parameters: 200 trees, the number of predictors is 7, the proportion of subsample is 0.45, the maximum number of levels is 5, the minimum number of cases is 50, the maximum number of nodes is 100, and the minimum number in child nodes is 10.

After the classifiers were developed, the three best models were combined based on a majority voting mechanism. The best models for each target were selected based on their performance in 10-fold cross-validation (10-fold CV). They were then externally validated based on the test sets. The performance of the models was evaluated using the statistical parameters calculated from the confusion matrix, including accuracy, precision, sensitivity, F1-score, and MCC [63]. In addition, the area under the ROC curve was computed for each model. The confusion matrix used to calculate the statistical parameters is shown in Table 8.

The performance parameters of each ML model were calculated using the following equations:

(1) $$Accuracy=\frac{Tp+Tn}{TPP+TNP}$$

(2) $$Precision=\frac{Tp}{TPP}$$

(3) $$Sensitivity=\frac{Tp}{TPE}$$

(4) $$F1-score=\frac{2Tp}{TPP+TPE}$$

(5) $$MCC=\frac{\left(Tp\times Tn)-(Fn\times Fp\right)}{\sqrt{TPE\times TPP\times TNP\times TNE}}$$

where accuracy is the rate of correct prediction of the model; sensitivity is the rate of compounds correctly classified as active inhibitor relative to the total number of actual inhibitors; precision is the rate of compounds correctly classified as inhibitors relative to the total number of predictive inhibitors; the *F*1-score is the harmonic mean of precision and sensitivity; and the Matthews correlation coefficient (*MCC*) is another robust measure of classification performance. A coefficient of +1 represents perfect prediction, 0 represents average random prediction, and −1 represents worst possible prediction.

In order to apply the QSAR models, the applicability domain (AD) must be defined according to the organization for economic cooperation and development (OECD) principles [64]. There are several approaches to generate AD, including range-based methods, geometric methods, distance-based methods, and probability-density-distribution-based methods [65]. In this study, we used distance-based methods, which compute the distance of the query compounds from a defined point in the descriptor space of the training data. Here, we employed the 3-nearest neighbors (3-NN) algorithm using Euclidean distance to define the AD [65]. A new compound is predicted by the model to be within the AD if and only if:

(6) $$D_{i}\leq D_{k}+0.5\times S_{k}$$

where *D_i_* is the average of the distances between compound *i* and its three nearest neighbors in the training set; *D_k_* is the average Euclidean distance between each compound of the training set and its three nearest neighbors in the descriptor space; and *S_k_* is the standard deviation of the distances between each compound of the training set and its three nearest neighbors in the descriptor space.

### 3.4. Rule-Based Query Selection and Virtual Design of Dual-Target Inhibitors

For the virtual design of novel dual-target inhibitors, a rule-based query was used for the first time. In this approach, representative compounds are selected to define key fragments. To this end, query molecules are used as templates for the virtual design, taking into account three main aspects of the selected molecules: (i) they must have strong activity, (ii) they must be representative of the group of virtually designed structures, and (iii) they must be positively predicted in all three ML models (CART, CHAID, and RF). To select the query that satisfies the above criteria and increases the probability of successful design, this rule-based approach was used. The procedure is as follows:

First, the CART and CHAID models are used to filter and the compounds in the active and test sets with the highest prediction accuracy are selected. In the second step, the previously filtered-out cases are checked in the remaining RF models. In the next step, all cases predicted to be active according to this step-by-step rule are used to perform a *k*-means cluster analysis (*k*-MCA). The best clusters are chosen based on the following criteria: (i) the activity of the compounds in the selected cluster is slightly equal, with few outliers; (ii) the clusters with the best activity (the lowest IC_50_ average); and (iii) the number of compounds in the selected cluster is greater than 9 (not included). After this selection, the compounds within the cluster are ranked based on their IC_50_ values and distance measurements calculated using the *k*-MCA. In this sense, some representative compounds (2 or 3) with the highest activity against each target are selected, which should be located in the center of the cluster, i.e., far from the edge of the cluster and with a distance different from the maximum and minimum distance of the cluster. Then, these compounds are fragmented based on the structure–activity relationships extracted from the best rules of the current work and previously published research. At this stage, the standard Murcko-type decomposition algorithm implemented in RDKit was applied to extract the molecular framework and side chains of all the molecules [50]. Accordingly, the framework focuses on the ring systems and the linker atoms connecting the ring systems. The side chains include the remaining part of the molecule (non-linker, non-ring atoms). In the RDKit package, the Murcko scaffolds are defined based on the graph framework, which only considers the connectivity but not atom type, hybridization, and bond order [66]. Therefore, we had to verify the fragment structures from the most frequent Murcko scaffolds of the decomposition matrix. Since the frameworks are the most important substructures affecting activity, they are considered for the design of new inhibitors. We therefore assembled the most frequent frameworks among Murcko scaffolds in the clusters of both AChE and BACE1 inhibitors and then generated their derivatives by adding the side chains to the designed compounds. The activity of these new derivatives is finally predicted by all QSAR models. Only compounds that were actively predicted by all three models were considered as dual-targeted inhibitors.

### 3.5. Docking Simulations

Molecular docking simulations were performed for all the compounds against the enzymes AChE and BACE1. For this purpose, human BACE1 (beta secretase) in complex with cyclohexanecarboxylic acid (PDB ID: 4O2B) and recombinant human AChE in complex with donepezil (PDB ID: 4EY7) were retrieved from the Protein Data Bank [45,67]. They were prepared for docking assays according to the standard protocol implemented in the ICM Pro (x64) software [46]. During protein preparation, all the water molecules were removed, all hydrogens were optimized, HisProAsnGlnCys geometry was optimized, the MMFF forcefield was adjusted, and the binding site of colchicine was predicted using ICMpocketfinder [18,28]. The 2D structures of the designed compounds were created using ChemDraw 20.1.1, then imported into the ICM Pro software and converted to 3D conformations for docking. Before docking the synthesized compounds, the co-crystallized ligands were removed and redocked into the binding sites of AChE and BACE1. The conformational sampling is based on the biased probability Monte Carlo (BPMC) procedure. The ICM scoring function is of GBSA/MM type [46] and is weighted according to the following parameters: (i) internal force-field energy of the ligand, (ii) entropy loss of the ligand between bound and unbound states, (iii) ligand–receptor hydrogen bond interactions, (iv) polar and non-polar solvation energy differences between bound and unbound states, (v) electrostatic energy, (vi) hydrophobic energy, and (vii) hydrogen bond donor or acceptor desolvation. For each ligand, 50 conformations were generated, and the conformations with best binding scores and key interactions similar to those of the native ligands were selected for further studies. Docking results were then visualized using BIOVIA Discovery Studio Visualizer 2021.

## 4. Conclusions

AzD has a complex pathophysiology involving aggregation of multiple proteins, including those involved in neurotransmission, the oxidative stress response, and neuroinflammation. Multi-target drug design may be a promising approach to identify potential drug candidates against AzD. In this context, a rational drug design method was proposed in this study with the aim of discovering potential dual AChE and BACE1 inhibitors against AzD. To develop the activity rules of each target, classification trees were constructed based on large databases of bioactive compounds from the ChEMBL chemical library. The best rules for predicting inhibitory activity against each target were identified and can be effectively used for chemical characterization and virtual screening of novel inhibitors. In total, we obtained four AChE inhibition rules based on eight structural indices with a prediction accuracy of 76.5–88.9%. Similarly, three BACE1 inhibition rules were obtained with six chemical features, which had a very good accuracy of 75.3–98.1%. Then, a virtual assay was performed integrating all the activity rules and QSAR models. In this way, we were able to identify dual-targeted inhibitors from the original data set of >3500 bioactive compounds with experimental data from the ChEMBL database. Some structures were determined to have activity against the AChE and BACE1 enzymes for the first time. Docking simulations on the dual inhibitors identified in this assay clearly demonstrated their ability to bind with the catalytic domains of both enzymes. These results confirmed the applicability of the elaborated rule-based and QSAR models for screening active dual AChE and BACE1 inhibitors.

In the subsequent application, we focused on two small chemical spaces determined by 67 AChE and 144 BACE1 inhibitors using the activity rules. Using a fragment drug design approach based on a Murcko-type decomposition algorithm implemented in RDKit, 192 active fragments of AChE inhibitors and 240 fragments of BACE1 inhibitors were extracted and ranked according to their abundance in the active inhibitors against each target. New dual inhibitors were designed by assembling the most abundant fragments. These compounds were then filtered through all the QSAR models, taking into account the applicability domain of each model. Finally, by integrating the results from QSAR and docking simulations, the most active dual AChE and BACE1 inhibitors were identified. In conclusion, the computational method developed in this study could be used for the development of novel dual- and multi-target agents to treat AzD. The effectiveness of the design would then be confirmed after synthesis in the next step by biological investigation of the new compounds with cell- and animal-based models.

## Figures and Tables

**Table 1:** Overview of the performance parameters of the six machine learning models.

Model	Accuracy		Precision		Sensitivity		F1-Score		MCC ^1^		AUC
	Training	Test	Training	Test	Training	Test	Training	Test	Training	Test	Test
AChE-CART	0.85	0.83	0.66	0.64	0.80	0.77	0.72	0.70	0.62	0.59	0.83 ± 0.024
AChE-CHAID	0.86	0.85	0.71	0.68	0.78	0.76	0.74	0.72	0.65	0.61	0.85 ± 0.024
AChE-RF	0.87	0.86	0.71	0.69	0.84	0.81	0.77	0.75	0.68	0.66	0.89 ± 0.013
BACE1-CART	0.83	0.82	0.74	0.72	0.89	0.87	0.81	0.79	0.67	0.64	0.83 ± 0.024
BACE1-CHAID	0.82	0.80	0.74	0.76	0.77	0.73	0.77	0.75	0.62	0.59	0.83 ± 0.020
BACE1-RF	0.85	0.83	0.79	0.75	0.83	0.85	0.81	0.80	0.68	0.66	0.88 ± 0.016

^1^ Matthews correlation coefficient.

**Table 2:** Applicability domains of all six machine learning models.

Models	Applicability Domain (AD)	Outliers of Test Set
AChE-CART	*D_i_* ≤ *D_k_* + 0.5 × *S_k_* = 0.3311 + 0.5 × 0.3447 = 0.5035	121/590
AChE-CHAID	*D_i_* ≤ *D_k_* + 0.5 × *S_k_* = 1.1011 + 0.5 × 0.7766 = 1.4894	130/590
AChE-RF	*D_i_* ≤ *D_k_* + 0.5 × *S_k_* = 5.5469 + 0.5 × 3.2381 = 7.1660	121/590
BACE1-CART	*D_i_* ≤ *D_k_* + 0.5 × *S_k_* = 0.3826 + 0.5 × 0.4915 = 0.6284	43/464
BACE1-CHAID	*D_i_* ≤ *D_k_* + 0.5 × *S_k_* = 0.5055 + 0.5 × 0.5208 = 0.7659	66/464
BACE1-RF	*D_i_* ≤ *D_k_* + 0.5 × *S_k_* = 3.9731 + 0.5 × 2.3496 = 5.1479	95/464

**Table 3:** Molecular descriptors included in the four AChE rules.

Descriptors ID	Description	Descriptor Family
*C-006*	CH_2_RX	Atom-centered fragments
*H-051*	H attached to alpha C	Atom-centered fragments
*O-058*	=O	Atom-centered fragments
*F09[C-N]*	Frequency of C-N at topological distance 9 (order 9)	2D atom pairs
*Yindex*	Balaban Y index	Information indices
*SpMaxA_EA(ed)*	Normalized leading eigenvalue from edge adjacency mat. weighed by edge degree	Edge adjacency indices
*SM15_EA(dm)*	Spectral moment of order 15 from edge adjacency matrix weighted by dipole moment	Edge adjacency indices
*Eta_betaP_A*	Eta pi and lone pair average VEM count	ETA indices

**Table 4:** Molecular descriptors included in the three BACE1 rules.

Descriptors ID	Description	Descriptor Family
*nR10*	Number of 10-membered rings	Ring descriptors
*nCIC*	Number of rings (cyclomatic number)	Ring descriptors
*IC1*	Information content index (neighborhood symmetry of 1 order)	Information indices
*GGI9*	Topological charge index of order 9	2D autocorrelations
*SM06_EA(ri)*	Spectral moment of order 6 from edge adjacency matrix weighted by resonance integral	Edge adjacency indices
*P_VSA_e_3*	P_VSA-like on Sanderson electronegativity, bin 3	P_VSA-like descriptors

**Table 5:** AChE and BACE1 dual inhibitors screened by experimental and rule-based approaches.

Molecule ChEMBL ID	Activity Rules	QSAR Prediction	Docking Scores (kCal/mol)	AChE IC_50_ (nM)	BACE1 IC_50_ (nM)	Reference ^1^
CHEMBL3355580	BACE1 Rule 3	2/3	−16.12	18.3	-	[30]
CHEMBL3600552	BACE1 Rule 1	2/3	−11.78	3.46	-	[31]
CHEMBL3600553	BACE1 Rule 1	2/3	−7.93	6.46	-	[32]
CHEMBL3600554	BACE1 Rule 1	2/3	−12.14	10.1	-	[32]
CHEMBL3600555	BACE1 Rule 1	2/3	−10.12	1.48	-	[30]
CHEMBL3600556	BACE1 Rule 1	2/3	−7.24	3.53	-	[30]
ChEMBL3403874	BACE1 Rule 1	2/3	−6.92	6.9	-	[33]
CHEMBL3632989	BACE1 Rule 1	3/3	−12.63	80.0	-	[34]
CHEMBL440983	BACE1 Rule 3	2/3	−10.77	6.65	-	[35]
CHEMBL238230	BACE1 Rule 3	2/3	−8.27	1.83	-	[35]
CHEMBL226335	BACE1 Rule 2	2/3	−6.16	12.0	-	[29]
CHEMBL195241	BACE1 Rule 3	2/3	−11.67	4.1	-	[36]
CHEMBL179455	BACE1 Rule 1	2/3	−10.54	1.55	-	[37]
CHEMBL3343885	BACE1 Rule 3	2/3	−7.97	92.6	-	[38]
CHEMBL4286601	BACE1 Rule 3	3/3	−10.72	6.3	-	[39]
CHEMBL3403878	BACE1 Rule 1	2/3	−9.11	32.5	-	[33]
CHEMBL3403877	BACE1 Rule 1	2/3	−7.75	17.3	-	[33]
CHEMBL1819176	BACE1 Rule 1	2/3	−11.98	1.05		[40]
CHEMBL1196204	BACE1 Rule 1	2/3	−8.32	19.3	-	[41]
CHEMBL3343882	BACE1 Rule 3	2/3	−10.96	98.2	-	[41]
CHEMBL4278287	BACE1 Rule 3	3/3	−11.23	38.0	-	[39]
CHEMBL3400187	BACE1 Rule 1	2/3	−9.54	21.6	-	[33]
CHEMBL4210729	BACE1 Rule 3	2/3	−9.97	41.9	-	[42]
CHEMBL4213591	BACE1 Rule 3	2/3	−12.31	51.7	-	[42]
CHEMBL4293418	BACE1 Rule 1	3/3	−15.31	3.6	-	[39]
CHEMBL4282154	BACE1 Rule 3	3/3	−19.64	2.1	-	[39]
CHEMBL4215154	BACE1 Rule 1	2/3	−12.65	89.6	-	[42]
CHEMBL4217346	BACE1 Rule 3	2/3	−11.96	94.1	-	[42]
CHEMBL4290039	BACE1 Rule 3	3/3	−10.27	22.0	-	[39]
CHEMBL4215217	BACE1 Rule 1	2/3	−17.45	74.5	-	[42]
CHEMBL4278686	BACE1 Rule 3	3/3	−14.42	6.4	-	[39]
CHEMBL4285581	BACE1 Rule 1	3/3	−14.23	23.0	-	[39]
CHEMBL255838	AChE Rule 1	2/3	−17.70	-	5.6	[43]
CHEMBL2407494	AChE Rule 3	2/3	−14.39	-	76.0	[44]

^1^ References of experimental data.

**Table 6:** Predictions of the designed inhibitors targeting AChE and BACE1 enzymes.

Cpd. ID	AChE Rule	AChE QSAR Prediction	AChE Docking Scores (kCal/mol)	BACE1 Rule	AChE QSAR Prediction	BACE1 Docking Scores (kCal/mol)
M02	-	2/3	−14.84	Rule 1	2/3	−10.75
M06	-	2/3	−12.31	Rule 1	3/3	−10.92
M07	Rule 4	2/3	−15.54	Rule 1	2/3	−14.89
M09	-	3/3	−14.70	Rule 2	2/3	−11.80
M13	-	3/3	−18.30	Rule 3	3/3	−15.82
M14	Rule 4	3/3	−19.30	-	2/3	−16.38
M17	Rule 4	2/3	−21.20	-	3/3	−12.33
M18	Rule 4	3/3	−18.71	Rule 1	3/3	−16.14
M19	Rule 4	3/3	−16.90	-	2/3	−15.32
M27	Rule 3	3/3	−19.43	Rule 1	3/3	−16.11
M30	Rule 3	3/3	−17.42	Rule 1	3/3	−14.75
M96	-	3/3	−18.80	Rule 2	3/3	−17.03
M97	-	3/3	−18.50	Rule 2	3/3	−16.52

**Table 7:** Comparison with previous QSAR studies dealing with AChE and BACE1 dual inhibitors.

Year	Methods	Molecular Descriptors	Database	QSAR Model Performance	References
2014	- Fragment-based QSAR using partial least square (PLS) regression - Molecular docking	705 2D descriptors by vLifeMDS software	20 1,4-dihydropyridine (DHP) derivatives	Best models: - Training set: R^2^ = 0.85 - Cross validation: Q^2^ = 0.68	Goyal et al. [12]
2020	- 2D-QSAR model using PLS regression - 3D pharmacophore and molecular docking for virtual screening	2D molecular descriptors by MOE 2008.10	72 AChE and 215 BACE1 inhibitors (varied structures)	AChE models: - R^2^ (training) = 0.70; R^2^ (LOO) = 0.57; Q^2^ (external validation) = 0.78 BACE1 models - R^2^ (training) = 0.80; R^2^ (LOO) = 0.77; Q^2^ (external validation) = 0.83	Tran et al. [57]
2022	- Classification algorithm: iterative stochastic elimination (ISE) - Molecular docking for virtual screening	Tanimoto index (TI) calculated by OpenBabel using FP2 fingerprints	- 3 AChE active molecule lists included 195–428 compounds - 4 BACE1 active molecule lists included 194–1317 compounds	8 AChE models: - MCC = 0.56–0.79; average AUC = 0.77–0.95 5 BACE1 models: - MCC = 0.65–0.82; average AUC = 0.78–0.97	Stern et al. [14]
2022	Regression algorithms: - Genetic function approximation (GFA) and nonlinear method, - Support vector machine (SVM) and artificial neural network (ANN)	2D descriptors: spatial, structural, thermodynamics, electro-topological and E-state indices	57 AChE and 53 BACE1 inhibitors (varied structures)	AChE models: - R^2^ (training) = 0.87, Q^2^ (external validation) = 0.86 BACE1 models: - R^2^ (training) = 0.82, Q^2^ (external validation) = 0.78	Dhamodharan and Mohan [13]
2023	- Rule-of-thumb - Classification algorithms: CART, CHAID, and RF	1100 and 1151 0-2D descriptors calculated using Dragon 6.0	ChEMBL databases including 1975 AChE inhibitors and 1549 BACE1 inhibitors	AChE models: - 4 rules with accuracies of 0.77–0.89 - 3 models: R^2^ (training) = 0.85–0.87; Q^2^ (test) = 0.83–0.86 BACE1 models: - 3 rules with accuracies of 0.75–0.98 - 3 models: R^2^ (training) = 0.82–0.85; Q^2^ (test) = 0.80–0.83	Current study

**Table 8:** Confusion matrix for the calculation of statistical parameters ^1^.

	Active Inhibitor (Predicted)	Inactive Inhibitor (Predicted)	Total (Experimental)
Active inhibitor (Experimental)	*Tp*	*Fn*	*Tp + Fn (TPE)*
Inactive inhibitor (Experimental)	*Fp*	*Tn*	*Fp + Tn (TNE)*
Total (Predicted)	*Tp + Fp (TPP)*	*Fn + Tn (TNP)*	*TPE + TNE = TPP + TNP*

^1^ *Tp*, true positive; *Fn*, false negative; *Fp*, false positive; *Tn*, true negative; *TPP*, total positive predicted; TNP, total negative predicted; *TPE*, total positive experimental; *TNE*, total negative experimental.

## Data Availability

Data is free of charge according to MDPI Research Data Policies.

## Supplementary Materials

The following supporting information can be downloaded at: https://www.mdpi.com/article/10.3390/molecules28083588/s1, Table S1. Molecule ChEMBL ID (https://www.ebi.ac.uk/chembl/) and labels of 1975 compounds in AChE database. Table S2. Molecule ChEMBL ID (https://www.ebi.ac.uk/chembl/) and labels of 1549 compounds in BACE1 database. Table S3. Molecular descriptors of CART and CHAID models for predicting AChE inhibitory activity. Table S4. Molecular descriptors of CART and CHAID models for predicting BACE1 inhibitory activity. Figure S1. Variable importance according to RF models for predicting (A) AChE and (B) BACE1 inhibitory activity. Figure S2. Distribution of experimental IC_50_ values of (A) 67 AChE inhibitors and (B) 144 BACE1 inhibitors among clusters. Table S5. Structures and bioactivities of 67 AChE inhibitors. Table S6. Structures and bioactivities of 144 BACE1 inhibitors. Table S7. Comparison of this study with previous published works on AchE. Table S8. Comparison of this study with previous published works on BACE1. Citation: [16,68,69,70,71,72,73,74,75,76,77,78,79,80,81,82,83,84,85,86,87,88,89,90,91,92,93,94,95].

## Sample Availability

Samples of the compounds are available from the authors.
