# Supplementary material for: Development of Activity Rules and Chemical Fragment Design for In Silico Discovery of AChE and BACE1 Dual Inhibitors against Alzheimer’s Disease
Source: Molecules. 2023 Apr 20;28(8):3588. doi: 10.3390/molecules28083588 (PMC10142303; doi:10.3390/molecules28083588)
Supplement: Supplementary file 1 [file molecules-28-03588-s001.zip › molecules-2319416-supplementary.pdf]

Development of activity rules and chemical fragment design for in silico discovery of AChE and BACE1 dual inhibitors against Alzheimer's disease

Le-Quang Bao[1], Daniel Baecker[2], Do Thi Mai Dung[1], Nguyen Phuong Nhung[1], Nguyen Thi Thuan[1], Phuong Linh Nguyen[3], Phan Thi Phuong Dung [1], Tran Thi Lan Huong[1], Bakhtiyor Rasulev[4], Gerardo M. Casanola-Martin[4], Nguyen-Hai Nam[1,*] and Hai Pham-The[1,*]

1 Hanoi University of Pharmacy, 13-15 Le Thanh Tong, Hoan Kiem, Hanoi 10000, Vietnam
2 Department of Pharmaceutical and Medicinal Chemistry, Institute of Pharmacy, University of Greifswald, Friedrich-Ludwig-Jahn-Straße 17, 17489 Greifswald, Germany
3 Drexel University, 3141 Chestnut St. Philadelphia, PA 19104, USA
4 Department of Coatings and Polymeric Materials, North Dakota State University, Fargo, ND 58102, USA
* Correspondence: haipham@hup.edu.vn (H. P-T.); namnh@hup.edu.vn (N-H. N.)

Supplementary materials:

Table S1. Molecule ChEMBL ID (https://www.ebi.ac.uk/chembl/) and labels of 1975 compounds in AChE database

Molecule ChEMBL ID	logIC$_{50}$ (nM)	Label	Molecule ChEMBL ID	logIC$_{50}$ (nM)	Label	Molecule ChEMBL ID	logIC$_{50}$ (nM)	Label
CHEMBL371787	3.272	Inactive	CHEMBL3752926	1.645	Active	CHEMBL3758282	3.851	Inactive
CHEMBL372202	-1.398	Active	CHEMBL4088659	4.093	Inactive	CHEMBL3338388	2.041	Inactive
CHEMBL1256415	4.176	Inactive	CHEMBL3133432	3.053	Inactive	CHEMBL3322142	1.974	Active
CHEMBL433041	4.342	Inactive	CHEMBL3597000	3.470	Inactive	CHEMBL3322159	2.442	Inactive
CHEMBL95	1.886	Active	CHEMBL3597002	3.758	Inactive	CHEMBL3338387	3.336	Inactive
CHEMBL186264	0.845	Active	CHEMBL3597010	3.663	Inactive	CHEMBL3597005	3.394	Inactive
CHEMBL188714	1.732	Active	CHEMBL278020	3.380	Inactive	CHEMBL25629	4.544	Inactive
CHEMBL594229	3.212	Inactive	CHEMBL3338998	4.703	Inactive	CHEMBL1782707	5.000	Inactive
CHEMBL218939	2.079	Inactive	CHEMBL3339002	4.470	Inactive	CHEMBL1783531	3.478	Inactive
CHEMBL187870	1.307	Active	CHEMBL659	2.740	Inactive	CHEMBL3764305	2.785	Inactive
CHEMBL130458	1.602	Active	CHEMBL194823	-0.597	Active	CHEMBL4086248	3.857	Inactive
CHEMBL636	3.618	Inactive	CHEMBL2160222	5.000	Inactive	CHEMBL4072914	3.825	Inactive
CHEMBL1243203	2.519	Inactive	CHEMBL219264	0.924	Active	CHEMBL363557	2.594	Inactive
CHEMBL450990	1.838	Active	CHEMBL502	1.360	Active	CHEMBL206093	1.556	Active
CHEMBL450506	3.738	Inactive	CHEMBL3356532	0.286	Active	CHEMBL426674	1.301	Active
CHEMBL2380671	3.706	Inactive	CHEMBL2234518	4.250	Inactive	CHEMBL2332536	4.072	Inactive
CHEMBL449941	1.860	Active	CHEMBL3356528	3.403	Inactive	CHEMBL156016	4.247	Inactive
CHEMBL452339	0.149	Active	CHEMBL252379	3.110	Inactive	CHEMBL73593	2.220	Inactive
CHEMBL1940612	0.988	Active	CHEMBL399040	3.080	Inactive	CHEMBL1651138	2.540	Inactive
CHEMBL92460	1.740	Active	CHEMBL3356950	2.908	Inactive	CHEMBL93241	2.950	Inactive
CHEMBL131219	0.602	Active	CHEMBL3356951	0.507	Active	CHEMBL3343711	2.479	Inactive
CHEMBL2234845	3.540	Inactive	CHEMBL3343925	2.173	Inactive	CHEMBL3355580	1.262	Active
CHEMBL1087194	2.892	Inactive	CHEMBL2234536	2.340	Inactive	CHEMBL3355579	0.826	Active
CHEMBL1819170	1.648	Active	CHEMBL2234535	2.360	Inactive	CHEMBL3133439	3.265	Inactive
CHEMBL1819171	1.217	Active	CHEMBL376186	3.338	Inactive	CHEMBL3087680	3.461	Inactive
CHEMBL1819176	0.021	Active	CHEMBL390737	3.461	Inactive	CHEMBL3417001	0.929	Active
CHEMBL365136	3.694	Inactive	CHEMBL2413560	3.013	Inactive	CHEMBL3417299	1.387	Active
CHEMBL1783515	0.954	Active	CHEMBL202661	-0.060	Active	CHEMBL3786666	4.130	Inactive
CHEMBL188325	2.716	Inactive	CHEMBL3289929	4.743	Inactive	CHEMBL1940614	1.462	Active
CHEMBL363588	3.992	Inactive	CHEMBL3289943	4.596	Inactive	CHEMBL3785269	1.081	Active
CHEMBL3884690	0.792	Active	CHEMBL489354	3.265	Inactive	CHEMBL54727	1.380	Active
CHEMBL92736	0.447	Active	CHEMBL19224	4.386	Inactive	CHEMBL3919289	4.000	Inactive
CHEMBL3819036	3.809	Inactive	CHEMBL375077	4.072	Inactive	CHEMBL3957099	3.806	Inactive

Table S1. (*Cont.*)

Molecule ChEMBL ID	logIC$_{50}$ (nM)	Label	Molecule ChEMBL ID	logIC$_{50}$ (nM)	Label	Molecule ChEMBL ID	logIC$_{50}$ (nM)	Label
CHEMBL3623548	2.083	Inactive	CHEMBL3116290	4.959	Inactive	CHEMBL3936989	3.941	Inactive
CHEMBL3623560	3.990	Inactive	CHEMBL3945872	3.777	Inactive	CHEMBL1819178	2.491	Inactive
CHEMBL3623551	0.356	Active	CHEMBL3892044	3.049	Inactive	CHEMBL219569	0.752	Active
CHEMBL1196495	1.480	Active	CHEMBL3410955	2.839	Inactive	CHEMBL3133442	3.387	Inactive
CHEMBL1651136	1.810	Active	CHEMBL3931517	4.633	Inactive	CHEMBL3597057	2.591	Inactive
CHEMBL1651137	2.100	Inactive	CHEMBL3600552	0.539	Active	CHEMBL1085505	4.294	Inactive
CHEMBL92629	2.510	Inactive	CHEMBL3600556	0.563	Active	CHEMBL3770978	3.708	Inactive
CHEMBL1083661	1.810	Active	CHEMBL4066365	3.903	Inactive	CHEMBL3770569	3.732	Inactive
CHEMBL172118	3.040	Inactive	CHEMBL499825	3.619	Inactive	CHEMBL3786937	2.121	Inactive
CHEMBL92958	2.079	Inactive	CHEMBL3582223	2.509	Inactive	CHEMBL3086276	1.987	Active
CHEMBL95020	0.556	Active	CHEMBL3582224	2.695	Inactive	CHEMBL3087676	4.188	Inactive
CHEMBL361869	1.633	Active	CHEMBL3582225	2.653	Inactive	CHEMBL92663	-0.097	Active
CHEMBL2237995	2.949	Inactive	CHEMBL2396913	4.949	Inactive	CHEMBL3322140	2.367	Inactive
CHEMBL362350	2.531	Inactive	CHEMBL468001	2.568	Inactive	CHEMBL2381401	3.246	Inactive
CHEMBL398628	2.939	Inactive	CHEMBL3582206	2.705	Inactive	CHEMBL3343929	2.307	Inactive
CHEMBL2332981	4.314	Inactive	CHEMBL3582211	4.124	Inactive	CHEMBL2380670	4.162	Inactive
CHEMBL2332540	4.374	Inactive	CHEMBL3582230	3.084	Inactive	CHEMBL3623559	2.387	Inactive
CHEMBL2332530	4.292	Inactive	CHEMBL2179375	3.477	Inactive	CHEMBL1783523	1.633	Active
CHEMBL2332532	4.425	Inactive	CHEMBL167911	3.364	Inactive	CHEMBL1783519	1.845	Active
CHEMBL2332542	4.057	Inactive	CHEMBL189122	0.797	Active	CHEMBL575813	2.267	Inactive
CHEMBL31574	1.079	Active	CHEMBL187818	0.415	Active	CHEMBL3948367	3.906	Inactive
CHEMBL3133434	2.940	Inactive	CHEMBL187330	3.410	Inactive	CHEMBL406645	4.220	Inactive
CHEMBL3133444	3.963	Inactive	CHEMBL3623563	4.228	Inactive	CHEMBL3112614	3.712	Inactive
CHEMBL239046	0.855	Active	CHEMBL223256	3.857	Inactive	CHEMBL3115024	4.022	Inactive
CHEMBL3922423	1.519	Active	CHEMBL490359	1.556	Active	CHEMBL3115021	3.158	Inactive
CHEMBL3597009	3.352	Inactive	CHEMBL329531	2.530	Inactive	CHEMBL3115042	3.709	Inactive
CHEMBL2380677	4.349	Inactive	CHEMBL1819180	2.658	Inactive	CHEMBL3115041	3.613	Inactive
CHEMBL2380674	3.522	Inactive	CHEMBL3751941	1.672	Active	CHEMBL3263733	3.322	Inactive
CHEMBL2380669	4.049	Inactive	CHEMBL2238002	3.886	Inactive	CHEMBL3263736	3.580	Inactive
CHEMBL1912059	-0.638	Active	CHEMBL4075825	4.274	Inactive	CHEMBL3353566	4.799	Inactive
CHEMBL2375480	3.250	Inactive	CHEMBL448799	4.176	Inactive	CHEMBL2413744	3.988	Inactive
CHEMBL129837	2.875	Inactive	CHEMBL127857	1.467	Active	CHEMBL3087677	4.013	Inactive
CHEMBL2234533	2.510	Inactive	CHEMBL3290190	3.444	Inactive	CHEMBL231160	0.176	Active
CHEMBL2234525	1.900	Active	CHEMBL1783514	0.477	Active	CHEMBL3322141	1.420	Active
CHEMBL2234524	4.610	Inactive	CHEMBL3623558	1.459	Active	CHEMBL3323051	4.752	Inactive
CHEMBL2234519	4.080	Inactive	CHEMBL540178	3.970	Inactive	CHEMBL491561	2.354	Inactive
CHEMBL2234511	3.280	Inactive	CHEMBL470867	2.732	Inactive	CHEMBL582883	1.954	Active
CHEMBL2234838	3.660	Inactive	CHEMBL3754146	2.619	Inactive	CHEMBL599644	1.716	Active
CHEMBL2234832	3.930	Inactive	CHEMBL368196	1.625	Active	CHEMBL604096	2.255	Inactive
CHEMBL2234546	2.940	Inactive	CHEMBL3116278	3.851	Inactive	CHEMBL474836	3.322	Inactive
CHEMBL398249	4.080	Inactive	CHEMBL3086277	2.364	Inactive	CHEMBL2380672	4.470	Inactive
CHEMBL252781	3.080	Inactive	CHEMBL3769833	3.792	Inactive	CHEMBL382260	-0.187	Active
CHEMBL1766150	4.299	Inactive	CHEMBL2011402	4.504	Inactive	CHEMBL381499	-1.000	Active
CHEMBL4069096	4.649	Inactive	CHEMBL197696	1.146	Active	CHEMBL374184	2.041	Inactive
CHEMBL3787223	1.230	Active	CHEMBL3823538	4.130	Inactive	CHEMBL597795	3.731	Inactive
CHEMBL3785207	2.014	Inactive	CHEMBL3770356	3.763	Inactive	CHEMBL592857	3.699	Inactive
CHEMBL2047228	4.111	Inactive	CHEMBL3770803	3.813	Inactive	CHEMBL591470	4.845	Inactive
CHEMBL3819024	4.487	Inactive	CHEMBL1834065	4.706	Inactive	CHEMBL1255901	1.079	Active
CHEMBL4065259	4.072	Inactive	CHEMBL3323059	2.352	Inactive	CHEMBL555300	4.041	Inactive
CHEMBL4085482	4.779	Inactive	CHEMBL3323046	2.728	Inactive	CHEMBL219172	2.279	Inactive

Table S1. (*Cont.*)

Molecule ChEMBL ID	$logIC_{50}$ (nM)	Label	Molecule ChEMBL ID	$logIC_{50}$ (nM)	Label	Molecule ChEMBL ID	$logIC_{50}$ (nM)	Label
CHEMBL1940619	4.387	Inactive	CHEMBL224981	3.365	Inactive	CHEMBL244762	2.097	Inactive
CHEMBL1243269	3.995	Inactive	CHEMBL194372	2.004	Inactive	CHEMBL390247	1.781	Active
CHEMBL519475	2.097	Inactive	CHEMBL374130	4.961	Inactive	CHEMBL276444	2.158	Inactive
CHEMBL362711	2.041	Inactive	CHEMBL24686	0.903	Active	CHEMBL243056	0.484	Active
CHEMBL3770071	3.806	Inactive	CHEMBL32823	-0.229	Active	CHEMBL373651	3.255	Inactive
CHEMBL3770206	3.663	Inactive	CHEMBL3338999	4.810	Inactive	CHEMBL1922540	4.813	Inactive
CHEMBL3883432	0.114	Active	CHEMBL3263735	3.732	Inactive	CHEMBL186144	0.903	Active
CHEMBL2160225	4.591	Inactive	CHEMBL3356534	1.393	Active	CHEMBL473866	0.188	Active
CHEMBL3289935	3.188	Inactive	CHEMBL3356536	-0.143	Active	CHEMBL1940617	4.810	Inactive
CHEMBL178792	3.633	Inactive	CHEMBL497755	4.513	Inactive	CHEMBL592624	3.845	Inactive
CHEMBL3263726	3.785	Inactive	CHEMBL1651132	0.110	Active	CHEMBL254016	0.519	Active
CHEMBL338755	-0.523	Active	CHEMBL420625	1.300	Active	CHEMBL238063	3.640	Inactive
CHEMBL242989	2.130	Inactive	CHEMBL434378	1.260	Active	CHEMBL393212	2.423	Inactive
CHEMBL195418	0.477	Active	CHEMBL1290152	3.861	Inactive	CHEMBL115835	2.429	Inactive
CHEMBL468002	3.447	Inactive	CHEMBL390083	3.531	Inactive	CHEMBL24519	3.898	Inactive
CHEMBL1766019	4.398	Inactive	CHEMBL502877	1.410	Active	CHEMBL93619	0.851	Active
CHEMBL363391	3.037	Inactive	CHEMBL245055	3.356	Inactive	CHEMBL243055	0.713	Active
CHEMBL1912062	2.408	Inactive	CHEMBL242905	2.566	Inactive	CHEMBL391918	3.719	Inactive
CHEMBL2088781	2.380	Inactive	CHEMBL426441	-0.319	Active	CHEMBL3323049	3.623	Inactive
CHEMBL575620	2.566	Inactive	CHEMBL94	1.544	Active	CHEMBL3323043	2.825	Inactive
CHEMBL1651140	0.940	Active	CHEMBL277665	4.996	Inactive	CHEMBL1651244	1.200	Active
CHEMBL359570	-0.480	Active	CHEMBL219316	1.771	Active	CHEMBL1161714	1.460	Active
CHEMBL3343931	1.984	Active	CHEMBL355740	1.926	Active	CHEMBL372491	1.447	Active
CHEMBL576005	1.875	Active	CHEMBL491527	4.435	Inactive	CHEMBL186090	2.875	Inactive
CHEMBL220905	3.623	Inactive	CHEMBL597794	2.041	Inactive	CHEMBL186042	0.663	Active
CHEMBL374729	2.878	Inactive	CHEMBL329231	-0.240	Active	CHEMBL3393675	4.663	Inactive
CHEMBL365678	3.320	Inactive	CHEMBL595115	1.491	Active	CHEMBL216159	0.719	Active
CHEMBL417915	2.881	Inactive	CHEMBL593933	1.924	Active	CHEMBL1618217	2.000	Inactive
CHEMBL3323042	2.334	Inactive	CHEMBL430403	2.260	Inactive	CHEMBL1914495	4.950	Inactive
CHEMBL3323065	2.914	Inactive	CHEMBL511468	3.149	Inactive	CHEMBL140476	-0.490	Active
CHEMBL3339000	4.391	Inactive	CHEMBL590031	3.477	Inactive	CHEMBL244838	3.090	Inactive
CHEMBL403260	1.560	Active	CHEMBL340427	-0.523	Active	CHEMBL245047	2.854	Inactive
CHEMBL238062	3.492	Inactive	CHEMBL2047229	3.973	Inactive	CHEMBL395280	2.778	Inactive
CHEMBL242661	1.708	Active	CHEMBL2047230	3.996	Inactive	CHEMBL3335048	2.954	Inactive
CHEMBL243270	1.789	Active	CHEMBL2234839	4.110	Inactive	CHEMBL3343708	2.729	Inactive
CHEMBL230434	1.628	Active	CHEMBL2234831	3.230	Inactive	CHEMBL3335025	3.447	Inactive
CHEMBL2380668	4.196	Inactive	CHEMBL2234542	4.230	Inactive	CHEMBL2413741	3.566	Inactive
CHEMBL2332539	3.941	Inactive	CHEMBL340625	0.756	Active	CHEMBL128452	1.845	Active
CHEMBL384563	1.991	Active	CHEMBL1766139	4.185	Inactive	CHEMBL2234516	4.690	Inactive
CHEMBL386541	1.607	Active	CHEMBL1288823	3.994	Inactive	CHEMBL2234841	4.520	Inactive
CHEMBL2236394	-0.086	Active	CHEMBL3310697	4.815	Inactive	CHEMBL375862	2.993	Inactive
CHEMBL62085	3.910	Inactive	CHEMBL3323039	2.303	Inactive	CHEMBL1770549	2.009	Inactive
CHEMBL1161715	2.210	Inactive	CHEMBL2419681	3.975	Inactive	CHEMBL1766017	4.274	Inactive
CHEMBL300143	1.000	Active	CHEMBL3322158	3.464	Inactive	CHEMBL241830	4.906	Inactive
CHEMBL188917	2.591	Inactive	CHEMBL20339	3.778	Inactive	CHEMBL244415	3.354	Inactive
CHEMBL211520	2.101	Inactive	CHEMBL330004	1.699	Active	CHEMBL389825	1.793	Active
CHEMBL212111	3.031	Inactive	CHEMBL370807	-0.347	Active	CHEMBL245046	2.491	Inactive
CHEMBL431844	1.500	Active	CHEMBL92142	2.342	Inactive	CHEMBL243130	2.563	Inactive
CHEMBL63173	4.322	Inactive	CHEMBL51934	2.215	Inactive	CHEMBL3582232	2.425	Inactive
CHEMBL225610	3.428	Inactive	CHEMBL241974	2.124	Inactive	CHEMBL376434	3.342	Inactive

Table S1. (*Cont.*)

Molecule ChEMBL ID	logIC$_{50}$ (nM)	Label	Molecule ChEMBL ID	logIC$_{50}$ (nM)	Label	Molecule ChEMBL ID	logIC$_{50}$ (nM)	Label
CHEMBL385778	3.114	Inactive	CHEMBL828	3.863	Inactive	CHEMBL243044	2.090	Inactive
CHEMBL1086014	4.365	Inactive	CHEMBL388714	3.201	Inactive	CHEMBL271710	2.515	Inactive
CHEMBL1084211	4.167	Inactive	CHEMBL396247	1.693	Active	CHEMBL1161720	3.180	Inactive
CHEMBL1084210	3.740	Inactive	CHEMBL513161	2.826	Inactive	CHEMBL132030	1.900	Active
CHEMBL1770550	4.338	Inactive	CHEMBL220294	2.281	Inactive	CHEMBL2071424	3.418	Inactive
CHEMBL1770553	4.342	Inactive	CHEMBL491358	1.653	Active	CHEMBL2064465	0.696	Active
CHEMBL2047528	1.874	Active	CHEMBL470715	2.577	Inactive	CHEMBL238230	0.262	Active
CHEMBL369661	1.838	Active	CHEMBL3323062	3.176	Inactive	CHEMBL3632858	0.833	Active
CHEMBL2375483	2.537	Inactive	CHEMBL192682	1.987	Active	CHEMBL2413746	4.029	Inactive
CHEMBL1082081	2.978	Inactive	CHEMBL278963	1.479	Active	CHEMBL3823464	3.666	Inactive
CHEMBL1076260	2.643	Inactive	CHEMBL1025	2.079	Inactive	CHEMBL3822670	3.694	Inactive
CHEMBL188753	2.447	Inactive	CHEMBL3403874	0.839	Active	CHEMBL3770577	3.799	Inactive
CHEMBL432376	2.079	Inactive	CHEMBL187591	4.477	Inactive	CHEMBL2425409	4.894	Inactive
CHEMBL93123	0.973	Active	CHEMBL189774	1.477	Active	CHEMBL222037	3.030	Inactive
CHEMBL188338	3.000	Inactive	CHEMBL363455	2.380	Inactive	CHEMBL3093180	2.022	Inactive
CHEMBL189517	2.041	Inactive	CHEMBL364585	1.898	Active	CHEMBL3093173	1.991	Active
CHEMBL292314	0.255	Active	CHEMBL15056	0.415	Active	CHEMBL1766143	4.407	Inactive
CHEMBL1082435	0.630	Active	CHEMBL109018	3.530	Inactive	CHEMBL3115030	3.675	Inactive
CHEMBL1651129	0.560	Active	CHEMBL3289936	2.944	Inactive	CHEMBL3115047	3.924	Inactive
CHEMBL1651247	-0.100	Active	CHEMBL1082082	2.398	Inactive	CHEMBL1819175	1.143	Active
CHEMBL1651250	1.630	Active	CHEMBL3115198	3.975	Inactive	CHEMBL189797	3.207	Inactive
CHEMBL1783527	1.881	Active	CHEMBL3335062	3.929	Inactive	CHEMBL219911	2.562	Inactive
CHEMBL1651135	2.180	Inactive	CHEMBL3335028	4.571	Inactive	CHEMBL3322160	1.188	Active
CHEMBL1651127	1.400	Active	CHEMBL74257	3.176	Inactive	CHEMBL3122167	1.813	Active
CHEMBL1651131	0.400	Active	CHEMBL1834066	4.243	Inactive	CHEMBL341658	0.320	Active
CHEMBL94059	3.410	Inactive	CHEMBL380830	2.785	Inactive	CHEMBL3415559	3.323	Inactive
CHEMBL1243297	4.614	Inactive	CHEMBL206237	3.826	Inactive	CHEMBL3415564	2.974	Inactive
CHEMBL1243012	1.602	Active	CHEMBL1084212	4.107	Inactive	CHEMBL3415568	2.428	Inactive
CHEMBL573102	2.502	Inactive	CHEMBL1082980	4.310	Inactive	CHEMBL3415574	3.203	Inactive
CHEMBL574487	3.061	Inactive	CHEMBL1766149	4.190	Inactive	CHEMBL3415576	2.762	Inactive
CHEMBL188011	3.000	Inactive	CHEMBL1766146	4.093	Inactive	CHEMBL3415581	2.836	Inactive
CHEMBL3582205	1.041	Active	CHEMBL1085780	3.940	Inactive	CHEMBL425739	1.584	Active
CHEMBL3582209	2.949	Inactive	CHEMBL575619	2.593	Inactive	CHEMBL195067	1.515	Active
CHEMBL369554	2.556	Inactive	CHEMBL3582200	3.679	Inactive	CHEMBL3233990	4.623	Inactive
CHEMBL1243388	3.520	Inactive	CHEMBL3582216	2.413	Inactive	CHEMBL433379	0.892	Active
CHEMBL395815	3.364	Inactive	CHEMBL594187	1.398	Active	CHEMBL143812	-0.490	Active
CHEMBL243272	1.134	Active	CHEMBL1076243	2.000	Inactive	CHEMBL173309	1.207	Active
CHEMBL241828	4.258	Inactive	CHEMBL1080631	2.724	Inactive	CHEMBL3085881	2.146	Inactive
CHEMBL242906	4.391	Inactive	CHEMBL62084	2.740	Inactive	CHEMBL499722	3.932	Inactive
CHEMBL265416	2.994	Inactive	CHEMBL65356	2.968	Inactive	CHEMBL131825	2.455	Inactive
CHEMBL413793	2.950	Inactive	CHEMBL325502	2.381	Inactive	CHEMBL241960	2.283	Inactive
CHEMBL3237628	4.854	Inactive	CHEMBL2064466	1.847	Active	CHEMBL243273	0.892	Active
CHEMBL3237633	4.233	Inactive	CHEMBL374981	0.706	Active	CHEMBL3417002	1.653	Active
CHEMBL1766151	4.412	Inactive	CHEMBL352500	0.653	Active	CHEMBL492098	3.937	Inactive
CHEMBL1084213	4.243	Inactive	CHEMBL2088687	3.944	Inactive	CHEMBL574847	2.279	Inactive
CHEMBL1082977	4.265	Inactive	CHEMBL94186	0.857	Active	CHEMBL205895	1.114	Active
CHEMBL1085781	4.146	Inactive	CHEMBL219262	2.587	Inactive	CHEMBL207777	1.987	Active
CHEMBL209392	1.867	Active	CHEMBL1243329	0.909	Active	CHEMBL3122169	4.199	Inactive
CHEMBL3769862	3.799	Inactive	CHEMBL474268	0.410	Active	CHEMBL3122170	3.618	Inactive
CHEMBL3770516	3.875	Inactive	CHEMBL1080813	2.699	Inactive	CHEMBL572453	2.204	Inactive

Table S1. (*Cont.*)

Molecule ChEMBL ID	logIC50 (nM)	Label	Molecule ChEMBL ID	logIC50 (nM)	Label	Molecule ChEMBL ID	logIC50 (nM)	Label
CHEMBL2088786	1.845	Active	CHEMBL3289930	4.884	Inactive	CHEMBL1241457	2.588	Inactive
CHEMBL244416	1.262	Active	CHEMBL3290189	3.979	Inactive	CHEMBL1243076	2.206	Inactive
CHEMBL3415566	2.736	Inactive	CHEMBL3356540	3.137	Inactive	CHEMBL243062	0.425	Active
CHEMBL1773482	1.398	Active	CHEMBL2047227	3.886	Inactive	CHEMBL3769811	3.806	Inactive
CHEMBL1773486	2.158	Inactive	CHEMBL3597001	3.814	Inactive	CHEMBL32329	2.255	Inactive
CHEMBL514966	3.260	Inactive	CHEMBL3884618	0.987	Active	CHEMBL3759090	3.982	Inactive
CHEMBL431519	1.602	Active	CHEMBL188823	0.763	Active	CHEMBL3763417	3.775	Inactive
CHEMBL356385	3.486	Inactive	CHEMBL93936	-0.097	Active	CHEMBL3764714	3.887	Inactive
CHEMBL243057	2.773	Inactive	CHEMBL3754341	3.316	Inactive	CHEMBL3763232	3.029	Inactive
CHEMBL242191	1.891	Active	CHEMBL2323354	4.798	Inactive	CHEMBL3763609	3.517	Inactive
CHEMBL242190	4.064	Inactive	CHEMBL189906	1.477	Active	CHEMBL3763820	3.188	Inactive
CHEMBL394348	1.238	Active	CHEMBL2375481	3.250	Inactive	CHEMBL3764679	3.587	Inactive
CHEMBL243269	0.978	Active	CHEMBL191729	3.427	Inactive	CHEMBL393621	2.476	Inactive
CHEMBL235014	1.294	Active	CHEMBL2171309	1.066	Active	CHEMBL189907	1.238	Active
CHEMBL364125	1.724	Active	CHEMBL3892148	2.270	Inactive	CHEMBL190322	0.909	Active
CHEMBL244619	2.170	Inactive	CHEMBL3910025	2.307	Inactive	CHEMBL225451	4.602	Inactive
CHEMBL413794	2.481	Inactive	CHEMBL4084714	3.952	Inactive	CHEMBL224469	4.013	Inactive
CHEMBL245054	3.305	Inactive	CHEMBL4094761	3.423	Inactive	CHEMBL244624	2.449	Inactive
CHEMBL427865	2.086	Inactive	CHEMBL4075021	4.658	Inactive	CHEMBL243310	2.305	Inactive
CHEMBL477772	2.968	Inactive	CHEMBL4101703	4.546	Inactive	CHEMBL362049	1.114	Active
CHEMBL3623549	0.946	Active	CHEMBL4062593	3.703	Inactive	CHEMBL362704	2.919	Inactive
CHEMBL3623566	3.398	Inactive	CHEMBL3234589	2.212	Inactive	CHEMBL2409142	2.689	Inactive
CHEMBL3623553	2.831	Inactive	CHEMBL3289933	4.886	Inactive	CHEMBL3585775	-1.571	Active
CHEMBL3623554	0.939	Active	CHEMBL3115048	3.892	Inactive	CHEMBL3632994	1.903	Active
CHEMBL3764488	3.827	Inactive	CHEMBL3115046	3.874	Inactive	CHEMBL3753360	2.068	Inactive
CHEMBL51085	3.792	Inactive	CHEMBL3754739	2.997	Inactive	CHEMBL507903	0.886	Active
CHEMBL3769576	3.663	Inactive	CHEMBL3234588	3.146	Inactive	CHEMBL597821	2.949	Inactive
CHEMBL3759013	4.173	Inactive	CHEMBL3910830	2.991	Inactive	CHEMBL2323356	4.725	Inactive
CHEMBL3818089	2.591	Inactive	CHEMBL3921785	3.004	Inactive	CHEMBL3585375	2.839	Inactive
CHEMBL332051	1.916	Active	CHEMBL4100760	1.569	Active	CHEMBL3632846	3.639	Inactive
CHEMBL415675	2.457	Inactive	CHEMBL3769834	3.748	Inactive	CHEMBL3632989	1.903	Active
CHEMBL241011	2.365	Inactive	CHEMBL1801815	3.996	Inactive	CHEMBL3356530	1.155	Active
CHEMBL240792	2.132	Inactive	CHEMBL1080274	2.477	Inactive	CHEMBL3356953	1.983	Active
CHEMBL3976003	3.502	Inactive	CHEMBL1243298	2.170	Inactive	CHEMBL3393673	4.778	Inactive
CHEMBL3928840	3.201	Inactive	CHEMBL4082739	4.131	Inactive	CHEMBL1288826	4.155	Inactive
CHEMBL3984034	3.508	Inactive	CHEMBL4087300	4.174	Inactive	CHEMBL445846	1.610	Active
CHEMBL2237999	3.906	Inactive	CHEMBL3752227	3.752	Inactive	CHEMBL248922	3.442	Inactive
CHEMBL488590	1.728	Active	CHEMBL3754694	3.875	Inactive	CHEMBL1651248	0.660	Active
CHEMBL606034	4.544	Inactive	CHEMBL3754448	3.839	Inactive	CHEMBL328468	-0.020	Active
CHEMBL450553	-0.432	Active	CHEMBL25149	4.903	Inactive	CHEMBL3586582	2.531	Inactive
CHEMBL74926	1.344	Active	CHEMBL4088091	3.322	Inactive	CHEMBL2237991	3.851	Inactive
CHEMBL427249	3.796	Inactive	CHEMBL4093532	4.088	Inactive	CHEMBL440983	0.823	Active
CHEMBL224433	3.450	Inactive	CHEMBL1161674	2.560	Inactive	CHEMBL2238004	3.512	Inactive
CHEMBL2413743	2.114	Inactive	CHEMBL1076259	2.431	Inactive	CHEMBL1834877	3.653	Inactive
CHEMBL604478	1.204	Active	CHEMBL1161718	2.700	Inactive	CHEMBL1834072	4.628	Inactive
CHEMBL605303	1.398	Active	CHEMBL132365	2.790	Inactive	CHEMBL59977	3.480	Inactive
CHEMBL252378	3.130	Inactive	CHEMBL397891	2.584	Inactive	CHEMBL61533	1.940	Active
CHEMBL2234541	4.150	Inactive	CHEMBL398406	1.916	Active	CHEMBL599057	2.279	Inactive
CHEMBL400675	2.970	Inactive	CHEMBL4126674	4.268	Inactive	CHEMBL599253	3.185	Inactive
CHEMBL3289928	4.440	Inactive	CHEMBL1243360	0.845	Active	CHEMBL3597556	3.894	Inactive

Table S1. (*Cont.*)

Molecule ChEMBL ID	$logIC_{50}$ (nM)	Label	Molecule ChEMBL ID	$logIC_{50}$ (nM)	Label	Molecule ChEMBL ID	$logIC_{50}$ (nM)	Label
CHEMBL3787116	3.207	Inactive	CHEMBL3410954	2.556	Inactive	CHEMBL382351	3.995	Inactive
CHEMBL3417008	3.435	Inactive	CHEMBL3753957	2.415	Inactive	CHEMBL3356537	0.966	Active
CHEMBL191461	0.255	Active	CHEMBL3355581	1.324	Active	CHEMBL71994	4.477	Inactive
CHEMBL3905695	2.690	Inactive	CHEMBL2011404	4.966	Inactive	CHEMBL416	2.881	Inactive
CHEMBL2011403	4.193	Inactive	CHEMBL208132	3.841	Inactive	CHEMBL388560	3.279	Inactive
CHEMBL64011	1.778	Active	CHEMBL377283	2.687	Inactive	CHEMBL2332543	4.439	Inactive
CHEMBL3786516	1.214	Active	CHEMBL4083601	3.857	Inactive	CHEMBL2332974	4.076	Inactive
CHEMBL1773488	2.410	Inactive	CHEMBL4091385	3.934	Inactive	CHEMBL3263725	3.623	Inactive
CHEMBL3764100	2.973	Inactive	CHEMBL3335060	2.954	Inactive	CHEMBL3263724	3.279	Inactive
CHEMBL2425412	4.899	Inactive	CHEMBL3335057	4.265	Inactive	CHEMBL3263727	3.613	Inactive
CHEMBL598824	2.708	Inactive	CHEMBL3335056	3.826	Inactive	CHEMBL3263730	3.415	Inactive
CHEMBL597001	3.207	Inactive	CHEMBL3335031	3.322	Inactive	CHEMBL3263737	3.833	Inactive
CHEMBL604715	3.462	Inactive	CHEMBL3786442	1.445	Active	CHEMBL1289274	3.568	Inactive
CHEMBL610779	2.799	Inactive	CHEMBL2206896	2.568	Inactive	CHEMBL1289615	4.121	Inactive
CHEMBL610219	4.458	Inactive	CHEMBL3809337	3.996	Inactive	CHEMBL4090559	4.845	Inactive
CHEMBL2088785	0.973	Active	CHEMBL191314	4.898	Inactive	CHEMBL1917826	3.663	Inactive
CHEMBL1288822	3.716	Inactive	CHEMBL195179	0.843	Active	CHEMBL4066805	4.046	Inactive
CHEMBL1288828	3.568	Inactive	CHEMBL191758	1.332	Active	CHEMBL1651130	0.990	Active
CHEMBL1243268	3.633	Inactive	CHEMBL185976	2.079	Inactive	CHEMBL3952144	2.430	Inactive
CHEMBL1243234	3.551	Inactive	CHEMBL366114	2.954	Inactive	CHEMBL4099340	3.427	Inactive
CHEMBL1243111	2.390	Inactive	CHEMBL362752	0.778	Active	CHEMBL218940	1.763	Active
CHEMBL2234529	2.780	Inactive	CHEMBL328715	2.908	Inactive	CHEMBL436302	2.369	Inactive
CHEMBL2234528	2.040	Inactive	CHEMBL65667	3.580	Inactive	CHEMBL597822	2.255	Inactive
CHEMBL2234520	4.970	Inactive	CHEMBL4285749	3.969	Inactive	CHEMBL598645	2.949	Inactive
CHEMBL2234512	3.590	Inactive	CHEMBL4294446	3.393	Inactive	CHEMBL3401159	1.544	Active
CHEMBL2234837	3.890	Inactive	CHEMBL4284235	2.987	Inactive	CHEMBL603622	1.602	Active
CHEMBL253603	3.950	Inactive	CHEMBL257540	2.979	Inactive	CHEMBL272700	2.741	Inactive
CHEMBL398250	4.140	Inactive	CHEMBL235548	3.583	Inactive	CHEMBL205340	1.973	Active
CHEMBL252578	3.730	Inactive	CHEMBL396190	2.924	Inactive	CHEMBL61486	3.580	Inactive
CHEMBL252580	3.810	Inactive	CHEMBL241536	2.545	Inactive	CHEMBL1289159	3.623	Inactive
CHEMBL253782	3.910	Inactive	CHEMBL1082978	3.991	Inactive	CHEMBL1289942	3.709	Inactive
CHEMBL253386	2.910	Inactive	CHEMBL1766142	4.305	Inactive	CHEMBL61930	2.670	Inactive
CHEMBL3235503	0.702	Active	CHEMBL1766140	4.422	Inactive	CHEMBL597405	3.693	Inactive
CHEMBL2234531	2.200	Inactive	CHEMBL1766148	4.384	Inactive	CHEMBL599058	2.580	Inactive
CHEMBL2234544	3.820	Inactive	CHEMBL1766141	4.427	Inactive	CHEMBL572480	2.000	Inactive
CHEMBL2234540	4.280	Inactive	CHEMBL62664	1.602	Active	CHEMBL507885	0.684	Active
CHEMBL1834064	4.140	Inactive	CHEMBL367795	1.914	Active	CHEMBL179320	3.423	Inactive
CHEMBL1912058	-0.327	Active	CHEMBL178770	1.362	Active	CHEMBL11805	2.230	Inactive
CHEMBL2234530	1.960	Active	CHEMBL1783522	2.474	Inactive	CHEMBL219406	2.228	Inactive
CHEMBL2234514	3.270	Inactive	CHEMBL4095032	3.763	Inactive	CHEMBL219400	2.529	Inactive
CHEMBL2234830	4.390	Inactive	CHEMBL4073501	3.556	Inactive	CHEMBL392324	1.845	Active
CHEMBL2234537	2.780	Inactive	CHEMBL595114	1.380	Active	CHEMBL243311	3.360	Inactive
CHEMBL2234523	4.330	Inactive	CHEMBL623	3.016	Inactive	CHEMBL1243140	2.919	Inactive
CHEMBL3133427	2.968	Inactive	CHEMBL4214932	2.364	Inactive	CHEMBL340391	1.000	Active
CHEMBL2064471	0.652	Active	CHEMBL3586200	3.228	Inactive	CHEMBL11833	1.477	Active
CHEMBL2064470	1.928	Active	CHEMBL3356954	1.580	Active	CHEMBL335167	2.839	Inactive
CHEMBL556581	0.605	Active	CHEMBL1834811	3.785	Inactive	CHEMBL61715	1.450	Active
CHEMBL3632852	3.398	Inactive	CHEMBL3752555	2.736	Inactive	CHEMBL302842	2.540	Inactive
CHEMBL3410953	2.792	Inactive	CHEMBL205335	2.152	Inactive	CHEMBL597188	1.748	Active
CHEMBL3632851	3.542	Inactive	CHEMBL381926	1.914	Active	CHEMBL86031	2.000	Inactive

Table S1. (*Cont.*)

Molecule ChEMBL ID	logIC$_{50}$ (nM)	Label	Molecule ChEMBL ID	logIC$_{50}$ (nM)	Label	Molecule ChEMBL ID	logIC$_{50}$ (nM)	Label
CHEMBL243271	0.352	Active	CHEMBL415799	2.352	Inactive	CHEMBL3343885	1.967	Active
CHEMBL244837	4.481	Inactive	CHEMBL222044	3.420	Inactive	CHEMBL3343930	2.064	Inactive
CHEMBL396050	2.090	Inactive	CHEMBL3632848	4.124	Inactive	CHEMBL1783535	2.791	Inactive
CHEMBL205940	1.748	Active	CHEMBL3764900	3.819	Inactive	CHEMBL1783524	2.201	Inactive
CHEMBL381782	3.741	Inactive	CHEMBL230702	0.373	Active	CHEMBL1783528	0.778	Active
CHEMBL195241	0.613	Active	CHEMBL158946	1.778	Active	CHEMBL2234534	2.730	Inactive
CHEMBL364545	3.441	Inactive	CHEMBL179455	0.190	Active	CHEMBL191416	3.476	Inactive
CHEMBL218443	1.865	Active	CHEMBL270248	3.626	Inactive	CHEMBL313231	1.618	Active
CHEMBL209924	3.006	Inactive	CHEMBL1773492	2.114	Inactive	CHEMBL418955	0.919	Active
CHEMBL1288821	3.785	Inactive	CHEMBL60119	2.000	Inactive	CHEMBL1651125	1.520	Active
CHEMBL1289825	3.836	Inactive	CHEMBL328102	1.996	Active	CHEMBL1651128	1.600	Active
CHEMBL1288829	3.623	Inactive	CHEMBL237870	2.713	Inactive	CHEMBL1651133	0.710	Active
CHEMBL92463	1.410	Active	CHEMBL243532	2.212	Inactive	CHEMBL1651243	0.460	Active
CHEMBL225198	3.954	Inactive	CHEMBL2332544	4.293	Inactive	CHEMBL1651245	1.720	Active
CHEMBL384886	1.858	Active	CHEMBL2332975	4.536	Inactive	CHEMBL1084790	1.080	Active
CHEMBL220141	2.130	Inactive	CHEMBL152675	4.343	Inactive	CHEMBL75013	2.290	Inactive
CHEMBL385269	1.713	Active	CHEMBL2332537	3.989	Inactive	CHEMBL513236	3.569	Inactive
CHEMBL178294	1.748	Active	CHEMBL2332535	4.213	Inactive	CHEMBL604255	3.778	Inactive
CHEMBL225567	3.301	Inactive	CHEMBL454594	4.257	Inactive	CHEMBL3235502	4.362	Inactive
CHEMBL134240	4.000	Inactive	CHEMBL2314726	1.633	Active	CHEMBL205321	3.535	Inactive
CHEMBL130373	4.620	Inactive	CHEMBL490360	3.630	Inactive	CHEMBL190528	2.716	Inactive
CHEMBL134057	0.970	Active	CHEMBL134342	0.000	Active	CHEMBL1770554	4.497	Inactive
CHEMBL1770548	2.297	Inactive	CHEMBL199670	-0.301	Active	CHEMBL2332538	4.240	Inactive
CHEMBL92775	2.004	Inactive	CHEMBL92955	1.146	Active	CHEMBL3115025	2.653	Inactive
CHEMBL87823	1.699	Active	CHEMBL2332541	3.899	Inactive	CHEMBL1940613	1.446	Active
CHEMBL187603	2.279	Inactive	CHEMBL2332531	3.843	Inactive	CHEMBL1940615	1.815	Active
CHEMBL130270	1.176	Active	CHEMBL3115037	3.371	Inactive	CHEMBL1940618	4.236	Inactive
CHEMBL606538	1.491	Active	CHEMBL491528	4.732	Inactive	CHEMBL1766144	4.334	Inactive
CHEMBL606348	1.462	Active	CHEMBL3310699	4.877	Inactive	CHEMBL3335067	4.255	Inactive
CHEMBL596236	1.342	Active	CHEMBL242345	3.110	Inactive	CHEMBL3335065	4.267	Inactive
CHEMBL240793	2.363	Inactive	CHEMBL394865	1.140	Active	CHEMBL3335054	3.806	Inactive
CHEMBL507304	1.146	Active	CHEMBL511948	4.241	Inactive	CHEMBL3335051	3.230	Inactive
CHEMBL1161716	3.230	Inactive	CHEMBL242836	1.111	Active	CHEMBL24416	4.845	Inactive
CHEMBL575621	2.661	Inactive	CHEMBL3318018	1.740	Active	CHEMBL227075	3.000	Inactive
CHEMBL512096	2.851	Inactive	CHEMBL3339007	4.886	Inactive	CHEMBL3122166	4.358	Inactive
CHEMBL303574	3.881	Inactive	CHEMBL597406	4.068	Inactive	CHEMBL3122150	2.974	Inactive
CHEMBL278342	1.415	Active	CHEMBL75121	1.648	Active	CHEMBL3632993	1.903	Active
CHEMBL2238000	2.544	Inactive	CHEMBL26125	2.258	Inactive	CHEMBL3415570	2.640	Inactive
CHEMBL2236393	-0.495	Active	CHEMBL3343932	2.185	Inactive	CHEMBL511575	4.610	Inactive
CHEMBL2234521	4.710	Inactive	CHEMBL2380676	3.526	Inactive	CHEMBL455790	4.021	Inactive
CHEMBL2234829	3.850	Inactive	CHEMBL1243170	3.398	Inactive	CHEMBL594186	1.708	Active
CHEMBL252380	2.850	Inactive	CHEMBL2160226	2.531	Inactive	CHEMBL606372	1.602	Active
CHEMBL404407	3.710	Inactive	CHEMBL2160223	4.569	Inactive	CHEMBL3322155	2.763	Inactive
CHEMBL1834061	4.223	Inactive	CHEMBL2160224	4.892	Inactive	CHEMBL3323036	2.155	Inactive
CHEMBL1834063	3.903	Inactive	CHEMBL2234522	3.850	Inactive	CHEMBL3323056	3.079	Inactive
CHEMBL1834074	4.000	Inactive	CHEMBL2234513	3.590	Inactive	CHEMBL1651126	1.360	Active
CHEMBL1834079	4.593	Inactive	CHEMBL2234844	3.290	Inactive	CHEMBL1084275	0.830	Active
CHEMBL1766018	4.360	Inactive	CHEMBL2234842	3.190	Inactive	CHEMBL3237630	3.959	Inactive
CHEMBL1766016	4.220	Inactive	CHEMBL2234840	4.320	Inactive	CHEMBL3263731	3.708	Inactive
CHEMBL3632992	2.000	Inactive	CHEMBL225611	3.796	Inactive	CHEMBL241827	3.491	Inactive

Table S1. (*Cont.*)

Molecule ChEMBL ID	logIC$_{50}$ (nM)	Label	Molecule ChEMBL ID	logIC$_{50}$ (nM)	Label	Molecule ChEMBL ID	logIC$_{50}$ (nM)	Label
CHEMBL1084808	4.396	Inactive	CHEMBL189283	2.580	Inactive	CHEMBL574474	2.283	Inactive
CHEMBL594870	1.491	Active	CHEMBL360832	1.954	Active	CHEMBL225366	3.894	Inactive
CHEMBL205967	3.384	Inactive	CHEMBL219063	2.382	Inactive	CHEMBL283509	0.000	Active
CHEMBL592433	4.477	Inactive	CHEMBL3754778	3.015	Inactive	CHEMBL2238003	3.972	Inactive
CHEMBL2234527	2.480	Inactive	CHEMBL441868	3.279	Inactive	CHEMBL3115044	3.728	Inactive
CHEMBL2234835	4.380	Inactive	CHEMBL1289160	3.494	Inactive	CHEMBL212162	3.128	Inactive
CHEMBL2234834	4.160	Inactive	CHEMBL1289273	3.633	Inactive	CHEMBL187812	4.292	Inactive
CHEMBL252579	3.110	Inactive	CHEMBL1289506	3.820	Inactive	CHEMBL1161717	2.200	Inactive
CHEMBL424833	1.547	Active	CHEMBL1161719	3.570	Inactive	CHEMBL2206900	3.705	Inactive
CHEMBL3263734	3.672	Inactive	CHEMBL382207	3.587	Inactive	CHEMBL126939	2.320	Inactive
CHEMBL1618106	2.431	Inactive	CHEMBL205231	1.431	Active	CHEMBL91291	0.760	Active
CHEMBL2206898	3.759	Inactive	CHEMBL378404	1.580	Active	CHEMBL478667	0.217	Active
CHEMBL2206895	2.519	Inactive	CHEMBL4065799	4.530	Inactive	CHEMBL2047529	2.076	Inactive
CHEMBL2206894	3.656	Inactive	CHEMBL491360	2.348	Inactive	CHEMBL2047224	3.886	Inactive
CHEMBL2206892	2.602	Inactive	CHEMBL491359	2.199	Inactive	CHEMBL3764609	3.228	Inactive
CHEMBL1243359	1.238	Active	CHEMBL3417300	1.207	Active	CHEMBL3582218	2.336	Inactive
CHEMBL1191355	4.763	Inactive	CHEMBL1080814	2.255	Inactive	CHEMBL4210020	0.612	Active
CHEMBL2425414	4.997	Inactive	CHEMBL2297280	1.968	Active	CHEMBL4212275	1.667	Active
CHEMBL1082979	4.422	Inactive	CHEMBL1770555	2.484	Inactive	CHEMBL4212713	1.823	Active
CHEMBL3116289	3.987	Inactive	CHEMBL1783516	1.041	Active	CHEMBL4175175	2.672	Inactive
CHEMBL1973869	3.863	Inactive	CHEMBL3632849	3.820	Inactive	CHEMBL2332546	4.197	Inactive
CHEMBL1834070	4.508	Inactive	CHEMBL3115020	3.634	Inactive	CHEMBL3262251	3.806	Inactive
CHEMBL1834075	4.462	Inactive	CHEMBL3116279	3.462	Inactive	CHEMBL1834068	4.068	Inactive
CHEMBL1834078	4.334	Inactive	CHEMBL1651139	0.040	Active	CHEMBL1834069	4.137	Inactive
CHEMBL3361270	2.204	Inactive	CHEMBL1651141	1.950	Active	CHEMBL4075061	1.954	Active
CHEMBL3597548	4.792	Inactive	CHEMBL1179697	-0.320	Active	CHEMBL4210316	-1.841	Active
CHEMBL3597055	4.180	Inactive	CHEMBL1651134	0.690	Active	CHEMBL3582213	2.272	Inactive
CHEMBL3623555	2.904	Inactive	CHEMBL65585	1.845	Active	CHEMBL4218191	-1.582	Active
CHEMBL3582228	2.693	Inactive	CHEMBL383242	3.740	Inactive	CHEMBL4209518	-1.532	Active
CHEMBL3582229	2.890	Inactive	CHEMBL207931	4.031	Inactive	CHEMBL4208641	0.210	Active
CHEMBL28	1.079	Active	CHEMBL4079853	4.844	Inactive	CHEMBL1783517	0.477	Active
CHEMBL1766145	4.459	Inactive	CHEMBL1819179	2.412	Inactive	CHEMBL1082981	3.944	Inactive
CHEMBL2419689	3.990	Inactive	CHEMBL1289045	3.326	Inactive	CHEMBL2413734	4.707	Inactive
CHEMBL128780	2.000	Inactive	CHEMBL1288825	4.086	Inactive	CHEMBL2413736	2.591	Inactive
CHEMBL128202	-0.252	Active	CHEMBL523311	4.566	Inactive	CHEMBL1834809	3.491	Inactive
CHEMBL2380667	3.764	Inactive	CHEMBL471581	3.217	Inactive	CHEMBL4288850	2.477	Inactive
CHEMBL3115029	4.039	Inactive	CHEMBL219550	1.230	Active	CHEMBL61929	3.720	Inactive
CHEMBL3115043	3.838	Inactive	CHEMBL266045	1.950	Active	CHEMBL1819169	1.838	Active
CHEMBL87014	1.342	Active	CHEMBL132285	2.892	Inactive	CHEMBL1819172	1.531	Active
CHEMBL3353040	1.860	Active	CHEMBL1241427	1.754	Active	CHEMBL3233993	4.708	Inactive
CHEMBL133441	3.800	Inactive	CHEMBL1171854	3.398	Inactive	CHEMBL3289934	4.656	Inactive
CHEMBL2425407	4.870	Inactive	CHEMBL362444	1.431	Active	CHEMBL3289937	1.756	Active
CHEMBL132260	2.420	Inactive	CHEMBL178132	3.587	Inactive	CHEMBL3360758	3.799	Inactive
CHEMBL2064464	1.173	Active	CHEMBL361409	1.973	Active	CHEMBL3115033	3.393	Inactive
CHEMBL66309	2.580	Inactive	CHEMBL313582	2.143	Inactive	CHEMBL4282558	3.405	Inactive
CHEMBL65018	1.903	Active	CHEMBL19557	4.954	Inactive	CHEMBL4167973	3.299	Inactive
CHEMBL4063045	3.924	Inactive	CHEMBL491562	4.292	Inactive	CHEMBL4169394	4.025	Inactive
CHEMBL4090583	4.386	Inactive	CHEMBL3900968	1.982	Active	CHEMBL521589	4.477	Inactive
CHEMBL187385	2.623	Inactive	CHEMBL3937964	2.348	Inactive	CHEMBL255230	4.642	Inactive
CHEMBL185799	2.398	Inactive	CHEMBL4095755	1.342	Active	CHEMBL271709	3.660	Inactive

Table S1. (*Cont.*)

Molecule ChEMBL ID	logIC$_{50}$ (nM)	Label	Molecule ChEMBL ID	logIC$_{50}$ (nM)	Label	Molecule ChEMBL ID	logIC$_{50}$ (nM)	Label
CHEMBL4294028	3.885	Inactive	CHEMBL3353567	4.724	Inactive	CHEMBL2234515	3.330	Inactive
CHEMBL3337471	3.883	Inactive	CHEMBL3310705	4.079	Inactive	CHEMBL2234543	4.230	Inactive
CHEMBL1819173	1.453	Active	CHEMBL535	3.769	Inactive	CHEMBL2234539	4.340	Inactive
CHEMBL2206893	3.894	Inactive	CHEMBL3786651	1.299	Active	CHEMBL253783	3.150	Inactive
CHEMBL524100	3.288	Inactive	CHEMBL1783534	1.398	Active	CHEMBL3623557	2.199	Inactive
CHEMBL1929043	4.190	Inactive	CHEMBL1783521	1.881	Active	CHEMBL2386558	3.212	Inactive
CHEMBL4286601	0.799	Active	CHEMBL3343714	2.980	Inactive	CHEMBL2088780	2.591	Inactive
CHEMBL4074566	3.724	Inactive	CHEMBL3335066	2.903	Inactive	CHEMBL2088783	3.491	Inactive
CHEMBL4127071	3.960	Inactive	CHEMBL3410951	2.453	Inactive	CHEMBL75110	1.720	Active
CHEMBL4100935	1.950	Active	CHEMBL1085527	1.338	Active	CHEMBL1651249	-0.310	Active
CHEMBL3582202	2.130	Inactive	CHEMBL4091317	3.929	Inactive	CHEMBL4214900	2.288	Inactive
CHEMBL3582210	3.430	Inactive	CHEMBL1834806	3.230	Inactive	CHEMBL1773487	2.155	Inactive
CHEMBL2234833	4.510	Inactive	CHEMBL1834808	3.255	Inactive	CHEMBL3916769	2.799	Inactive
CHEMBL3585780	-1.571	Active	CHEMBL2234836	4.430	Inactive	CHEMBL1834077	4.496	Inactive
CHEMBL2088784	2.342	Inactive	CHEMBL2234547	3.480	Inactive	CHEMBL1783530	2.872	Inactive
CHEMBL3764376	2.623	Inactive	CHEMBL2234538	4.110	Inactive	CHEMBL1783526	1.301	Active
CHEMBL2237993	3.818	Inactive	CHEMBL253602	4.320	Inactive	CHEMBL1783499	1.146	Active
CHEMBL3600555	0.170	Active	CHEMBL226335	1.079	Active	CHEMBL2409140	4.320	Inactive
CHEMBL3764762	3.260	Inactive	CHEMBL4205910	4.000	Inactive	CHEMBL3585777	-1.571	Active
CHEMBL164	1.079	Active	CHEMBL4292235	2.322	Inactive	CHEMBL3736339	3.013	Inactive
CHEMBL3597062	3.732	Inactive	CHEMBL4160790	2.880	Inactive	CHEMBL1940616	3.267	Inactive
CHEMBL2323352	4.947	Inactive	CHEMBL3582227	2.942	Inactive	CHEMBL3949439	3.543	Inactive
CHEMBL3623564	4.246	Inactive	CHEMBL3289926	2.964	Inactive	CHEMBL3754005	2.299	Inactive
CHEMBL446060	3.362	Inactive	CHEMBL3585781	-1.571	Active	CHEMBL3753508	3.227	Inactive
CHEMBL3623550	0.923	Active	CHEMBL3582204	3.522	Inactive	CHEMBL3752969	1.185	Active
CHEMBL3623556	2.041	Inactive	CHEMBL3582207	2.577	Inactive	CHEMBL2088782	2.079	Inactive
CHEMBL3623561	2.854	Inactive	CHEMBL3263732	3.748	Inactive	CHEMBL3818512	2.875	Inactive
CHEMBL598632	3.057	Inactive	CHEMBL3234592	1.886	Active	CHEMBL611941	3.100	Inactive
CHEMBL3410957	2.656	Inactive	CHEMBL3752543	1.033	Active	CHEMBL1834807	3.431	Inactive
CHEMBL3410956	2.355	Inactive	CHEMBL3735042	3.949	Inactive	CHEMBL4294389	3.871	Inactive
CHEMBL3632847	3.188	Inactive	CHEMBL599252	3.597	Inactive	CHEMBL4278883	3.954	Inactive
CHEMBL3632626	3.858	Inactive	CHEMBL603256	2.301	Inactive	CHEMBL3582226	2.819	Inactive
CHEMBL2332547	4.141	Inactive	CHEMBL1783525	0.845	Active	CHEMBL2323355	4.866	Inactive
CHEMBL3752974	4.255	Inactive	CHEMBL3822790	3.967	Inactive	CHEMBL3403877	1.238	Active
CHEMBL1288824	3.917	Inactive	CHEMBL1289507	3.934	Inactive	CHEMBL1082433	2.480	Inactive
CHEMBL572532	2.176	Inactive	CHEMBL1290043	3.863	Inactive	CHEMBL1651246	1.150	Active
CHEMBL3632853	3.544	Inactive	CHEMBL1288827	4.187	Inactive	CHEMBL459258	4.686	Inactive
CHEMBL1411769	3.869	Inactive	CHEMBL3093787	1.290	Active	CHEMBL4069444	3.863	Inactive
CHEMBL3416999	2.346	Inactive	CHEMBL3780448	4.907	Inactive	CHEMBL4090413	3.681	Inactive
CHEMBL2160220	4.831	Inactive	CHEMBL4207101	0.881	Active	CHEMBL2022933	2.813	Inactive
CHEMBL3122149	2.904	Inactive	CHEMBL4280612	-0.367	Active	CHEMBL3353042	2.634	Inactive
CHEMBL3122145	3.025	Inactive	CHEMBL3623552	0.716	Active	CHEMBL3415561	2.695	Inactive
CHEMBL3122171	4.114	Inactive	CHEMBL3751866	1.886	Active	CHEMBL128390	1.623	Active
CHEMBL2047531	2.143	Inactive	CHEMBL449467	1.201	Active	CHEMBL3263729	3.580	Inactive
CHEMBL3116287	3.623	Inactive	CHEMBL3338997	4.533	Inactive	CHEMBL1783518	0.477	Active
CHEMBL3310704	4.157	Inactive	CHEMBL3403878	1.512	Active	CHEMBL2332534	4.135	Inactive
CHEMBL1819174	2.452	Inactive	CHEMBL128260	0.447	Active	CHEMBL2332976	3.777	Inactive
CHEMBL3355594	4.083	Inactive	CHEMBL2234532	1.790	Active	CHEMBL2323353	4.911	Inactive
CHEMBL3823197	3.920	Inactive	CHEMBL2234526	1.720	Active	CHEMBL2323351	4.909	Inactive
CHEMBL3823578	3.870	Inactive	CHEMBL2234517	4.840	Inactive	CHEMBL2237994	2.716	Inactive

Table S1. (*Cont.*)

Molecule ChEMBL ID	logIC50 (nM)	Label	Molecule ChEMBL ID	logIC50 (nM)	Label	Molecule ChEMBL ID	logIC50 (nM)	Label
CHEMBL3781564	2.477	Inactive	CHEMBL3786873	1.489	Active	CHEMBL4083875	3.813	Inactive
CHEMBL1834060	4.199	Inactive	CHEMBL3884858	1.260	Active	CHEMBL3322157	2.017	Inactive
CHEMBL1834810	3.771	Inactive	CHEMBL2396910	4.776	Inactive	CHEMBL3115038	3.693	Inactive
CHEMBL4085450	1.900	Active	CHEMBL1254269	3.117	Inactive	CHEMBL2332550	4.200	Inactive
CHEMBL4128191	4.030	Inactive	CHEMBL3822559	3.580	Inactive	CHEMBL3597006	3.332	Inactive
CHEMBL129577	2.230	Inactive	CHEMBL3823317	3.556	Inactive	CHEMBL3605423	3.827	Inactive
CHEMBL1172893	3.785	Inactive	CHEMBL2047375	4.100	Inactive	CHEMBL3605418	3.979	Inactive
CHEMBL3623565	3.723	Inactive	CHEMBL1773483	1.380	Active	CHEMBL3353039	2.283	Inactive
CHEMBL3318017	1.531	Active	CHEMBL3343927	1.801	Active	CHEMBL3338993	4.116	Inactive
CHEMBL4081805	4.146	Inactive	CHEMBL3335052	3.964	Inactive	CHEMBL3339003	4.644	Inactive
CHEMBL4070380	3.898	Inactive	CHEMBL3343884	2.512	Inactive	CHEMBL468000	3.204	Inactive
CHEMBL2380675	3.881	Inactive	CHEMBL3343882	1.992	Active	CHEMBL3770479	3.778	Inactive
CHEMBL2071425	3.303	Inactive	CHEMBL3753850	1.387	Active	CHEMBL3764211	1.826	Active
CHEMBL3233992	4.788	Inactive	CHEMBL337675	1.415	Active	CHEMBL1079905	3.806	Inactive
CHEMBL2419685	3.728	Inactive	CHEMBL131210	1.892	Active	CHEMBL1424	3.954	Inactive
CHEMBL2419683	3.587	Inactive	CHEMBL3824239	3.188	Inactive	CHEMBL3785391	0.855	Active
CHEMBL1196204	1.286	Active	CHEMBL3824362	4.087	Inactive	CHEMBL3600553	0.810	Active
CHEMBL3310703	3.398	Inactive	CHEMBL3965046	4.491	Inactive	CHEMBL3093175	2.085	Inactive
CHEMBL3632850	3.827	Inactive	CHEMBL599254	3.121	Inactive	CHEMBL3093174	2.195	Inactive
CHEMBL3632990	2.176	Inactive	CHEMBL1766020	4.152	Inactive	CHEMBL3765293	3.310	Inactive
CHEMBL2413735	2.602	Inactive	CHEMBL4100298	1.591	Active	CHEMBL3335032	3.431	Inactive
CHEMBL3343920	2.185	Inactive	CHEMBL3735606	3.771	Inactive	CHEMBL3335064	3.613	Inactive
CHEMBL3343712	2.629	Inactive	CHEMBL3764433	3.850	Inactive	CHEMBL2413742	3.146	Inactive
CHEMBL3417000	2.507	Inactive	CHEMBL4061746	3.875	Inactive	CHEMBL350032	4.180	Inactive
CHEMBL3417003	2.083	Inactive	CHEMBL4079345	3.875	Inactive	CHEMBL2425417	4.959	Inactive
CHEMBL3087679	4.444	Inactive	CHEMBL4067481	3.820	Inactive	CHEMBL3416997	1.756	Active
CHEMBL2332979	3.526	Inactive	CHEMBL4100046	3.869	Inactive	CHEMBL3415560	2.481	Inactive
CHEMBL2332533	4.391	Inactive	CHEMBL4102655	3.845	Inactive	CHEMBL2237992	3.847	Inactive
CHEMBL2332549	4.096	Inactive	CHEMBL490338	1.851	Active	CHEMBL2237997	4.015	Inactive
CHEMBL3234591	2.415	Inactive	CHEMBL474837	3.196	Inactive	CHEMBL4075571	2.845	Inactive
CHEMBL3356538	2.814	Inactive	CHEMBL4130217	3.600	Inactive	CHEMBL4097374	3.857	Inactive
CHEMBL3323053	3.041	Inactive	CHEMBL2380673	3.631	Inactive	CHEMBL4082785	4.301	Inactive
CHEMBL3087674	2.778	Inactive	CHEMBL219405	2.490	Inactive	CHEMBL4072258	3.045	Inactive
CHEMBL1289392	4.049	Inactive	CHEMBL4164186	2.812	Inactive	CHEMBL309490	1.079	Active
CHEMBL3765697	3.061	Inactive	CHEMBL3822890	3.551	Inactive	CHEMBL3752356	1.800	Active
CHEMBL3763778	3.097	Inactive	CHEMBL3343705	2.455	Inactive	CHEMBL1773478	2.322	Inactive
CHEMBL1085526	1.068	Active	CHEMBL3416995	2.761	Inactive	CHEMBL1773480	2.857	Inactive
CHEMBL2238005	2.708	Inactive	CHEMBL1201217	2.258	Inactive	CHEMBL2297278	4.039	Inactive
CHEMBL2237998	4.281	Inactive	CHEMBL1801816	3.771	Inactive	CHEMBL128392	2.398	Inactive
CHEMBL3133440	3.476	Inactive	CHEMBL3335027	3.908	Inactive	CHEMBL4069776	2.447	Inactive
CHEMBL2238001	4.006	Inactive	CHEMBL2064469	2.288	Inactive	CHEMBL2375482	3.250	Inactive
CHEMBL1773479	2.740	Inactive	CHEMBL2064468	1.610	Active	CHEMBL151763	4.547	Inactive
CHEMBL1773485	1.833	Active	CHEMBL2064404	1.713	Active	CHEMBL3335072	4.328	Inactive
CHEMBL2147461	0.606	Active	CHEMBL887	1.279	Active	CHEMBL3335071	3.914	Inactive
CHEMBL243274	1.857	Active	CHEMBL164660	3.812	Inactive	CHEMBL3335069	3.919	Inactive
CHEMBL1459580	1.477	Active	CHEMBL3116292	3.826	Inactive	CHEMBL3335068	3.041	Inactive
CHEMBL3978533	3.760	Inactive	CHEMBL2047223	3.886	Inactive	CHEMBL3335053	4.137	Inactive
CHEMBL209863	3.506	Inactive	CHEMBL4068888	1.650	Active	CHEMBL3335030	3.863	Inactive
CHEMBL427232	1.322	Active	CHEMBL4129007	4.099	Inactive	CHEMBL2238007	4.364	Inactive
CHEMBL380050	2.454	Inactive	CHEMBL128551	1.146	Active	CHEMBL2419690	3.863	Inactive

Table S1. (*Cont.*)

Molecule ChEMBL ID	logIC$_{50}$ (nM)	Label	Molecule ChEMBL ID	logIC$_{50}$ (nM)	Label	Molecule ChEMBL ID	logIC$_{50}$ (nM)	Label
CHEMBL3586197	4.028	Inactive	CHEMBL1819177	1.083	Active	CHEMBL3289925	4.615	Inactive
CHEMBL259523	3.763	Inactive	CHEMBL491526	3.375	Inactive	CHEMBL3338991	4.020	Inactive
CHEMBL4291845	3.322	Inactive	CHEMBL4128454	3.942	Inactive	CHEMBL1773491	2.041	Inactive
CHEMBL2419688	1.114	Active	CHEMBL4129420	3.946	Inactive	CHEMBL3334506	4.152	Inactive
CHEMBL490507	2.663	Inactive	CHEMBL340807	4.375	Inactive	CHEMBL3335047	3.690	Inactive
CHEMBL4203672	2.076	Inactive	CHEMBL2237996	3.916	Inactive	CHEMBL3335029	3.114	Inactive
CHEMBL4202784	2.467	Inactive	CHEMBL3360757	4.546	Inactive	CHEMBL3353041	1.243	Active
CHEMBL1766147	4.100	Inactive	CHEMBL2391486	1.964	Active	CHEMBL4290992	4.146	Inactive
CHEMBL3086278	2.013	Inactive	CHEMBL2413740	3.942	Inactive	CHEMBL365904	1.602	Active
CHEMBL2206899	3.603	Inactive	CHEMBL3353568	4.833	Inactive	CHEMBL2147462	0.547	Active
CHEMBL3133433	2.519	Inactive	CHEMBL3322162	4.196	Inactive	CHEMBL204895	2.000	Inactive
CHEMBL2147460	0.714	Active	CHEMBL3770866	3.898	Inactive	CHEMBL3763973	3.549	Inactive
CHEMBL469638	4.303	Inactive	CHEMBL3763535	3.137	Inactive	CHEMBL3765093	3.143	Inactive
CHEMBL4174269	2.851	Inactive	CHEMBL3343922	1.963	Active	CHEMBL4125708	4.422	Inactive
CHEMBL3410952	2.728	Inactive	CHEMBL3356952	0.951	Active	CHEMBL4100407	2.160	Inactive
CHEMBL3233991	4.732	Inactive	CHEMBL3356529	3.789	Inactive	CHEMBL2297277	4.210	Inactive
CHEMBL1834071	4.307	Inactive	CHEMBL3343928	2.721	Inactive	CHEMBL2047226	3.996	Inactive
CHEMBL2238006	4.245	Inactive	CHEMBL470907	2.756	Inactive	CHEMBL3343923	2.201	Inactive
CHEMBL2206897	3.545	Inactive	CHEMBL3343924	2.486	Inactive	CHEMBL3115027	4.035	Inactive
CHEMBL2206891	2.477	Inactive	CHEMBL3343926	2.272	Inactive	CHEMBL3115040	3.599	Inactive
CHEMBL3885186	1.111	Active	CHEMBL3770203	3.763	Inactive	CHEMBL4103604	4.519	Inactive
CHEMBL1912060	0.425	Active	CHEMBL3771233	3.826	Inactive	CHEMBL611732	4.490	Inactive
CHEMBL4064773	3.940	Inactive	CHEMBL3769537	3.771	Inactive	CHEMBL2413559	3.328	Inactive
CHEMBL3289927	1.623	Active	CHEMBL239618	3.799	Inactive	CHEMBL3122168	2.745	Inactive
CHEMBL394753	3.869	Inactive	CHEMBL4242359	1.511	Active	CHEMBL2419687	1.732	Active
CHEMBL3763832	3.777	Inactive	CHEMBL3632988	2.322	Inactive	CHEMBL2419682	3.643	Inactive
CHEMBL3765569	3.511	Inactive	CHEMBL4285714	2.869	Inactive	CHEMBL3343713	2.591	Inactive
CHEMBL3818992	3.243	Inactive	CHEMBL4213147	2.491	Inactive	CHEMBL2425413	4.610	Inactive
CHEMBL4294109	3.100	Inactive	CHEMBL4217666	1.124	Active	CHEMBL2160227	2.380	Inactive
CHEMBL4293015	3.152	Inactive	CHEMBL4091683	2.833	Inactive	CHEMBL2160221	4.724	Inactive
CHEMBL4214951	3.666	Inactive	CHEMBL3901794	3.255	Inactive	CHEMBL3415558	2.305	Inactive
CHEMBL4203538	3.267	Inactive	CHEMBL2419684	1.491	Active	CHEMBL3415563	2.225	Inactive
CHEMBL4209755	2.691	Inactive	CHEMBL1783520	1.778	Active	CHEMBL3415569	3.501	Inactive
CHEMBL4204913	3.904	Inactive	CHEMBL3597054	3.714	Inactive	CHEMBL3415578	2.204	Inactive
CHEMBL4281440	1.724	Active	CHEMBL295124	2.000	Inactive	CHEMBL3415580	2.814	Inactive
CHEMBL4289981	3.540	Inactive	CHEMBL2413737	4.111	Inactive	CHEMBL3600554	1.004	Active
CHEMBL577016	3.708	Inactive	CHEMBL2011405	4.360	Inactive	CHEMBL3263728	3.398	Inactive
CHEMBL4102718	4.619	Inactive	CHEMBL4068340	4.161	Inactive	CHEMBL4064846	4.613	Inactive
CHEMBL4066908	3.653	Inactive	CHEMBL3086279	2.212	Inactive	CHEMBL4078463	4.375	Inactive
CHEMBL4075667	3.079	Inactive	CHEMBL4072825	2.892	Inactive	CHEMBL3237520	3.563	Inactive
CHEMBL3586592	2.978	Inactive	CHEMBL1773490	2.908	Inactive	CHEMBL1080386	0.778	Active
CHEMBL2413739	3.904	Inactive	CHEMBL224553	3.498	Inactive	CHEMBL3335070	4.438	Inactive
CHEMBL3417004	1.380	Active	CHEMBL4070462	3.857	Inactive	CHEMBL3335059	4.467	Inactive
CHEMBL89354	1.164	Active	CHEMBL1773481	2.336	Inactive	CHEMBL3335058	4.301	Inactive
CHEMBL3137700	2.455	Inactive	CHEMBL1773489	2.322	Inactive	CHEMBL3417006	3.013	Inactive
CHEMBL3133436	2.944	Inactive	CHEMBL3323068	4.083	Inactive	CHEMBL3335063	4.320	Inactive
CHEMBL3133443	3.386	Inactive	CHEMBL240047	3.851	Inactive	CHEMBL3415565	2.517	Inactive
CHEMBL2007996	-0.131	Active	CHEMBL4129147	4.385	Inactive	CHEMBL3415573	2.412	Inactive
CHEMBL1916768	4.658	Inactive	CHEMBL4070182	2.903	Inactive	CHEMBL4089033	3.615	Inactive
CHEMBL4161860	3.501	Inactive	CHEMBL4172587	3.688	Inactive	CHEMBL3884988	1.639	Active

Table S1. (*Cont.*)

Molecule ChEMBL ID	logIC50 (nM)	Label	Molecule ChEMBL ID	logIC50 (nM)	Label	Molecule ChEMBL ID	logIC50 (nM)	Label
CHEMBL4070769	3.623	Inactive	CHEMBL3819320	3.004	Inactive	CHEMBL3585776	-1.571	Active
CHEMBL3322156	2.600	Inactive	CHEMBL3586207	2.342	Inactive	CHEMBL3754291	1.687	Active
CHEMBL3322161	3.912	Inactive	CHEMBL2332545	3.830	Inactive	CHEMBL3753706	2.489	Inactive
CHEMBL3415579	3.303	Inactive	CHEMBL4104459	3.820	Inactive	CHEMBL3752451	2.627	Inactive
CHEMBL3115035	3.885	Inactive	CHEMBL4071803	4.700	Inactive	CHEMBL4210729	1.622	Active
CHEMBL3115032	3.791	Inactive	CHEMBL3237527	3.365	Inactive	CHEMBL4213591	1.713	Active
CHEMBL3116296	3.799	Inactive	CHEMBL3400187	1.334	Active	CHEMBL1344483	4.653	Inactive
CHEMBL3116293	3.914	Inactive	CHEMBL3338992	3.952	Inactive	CHEMBL4285481	2.491	Inactive
CHEMBL3930722	3.130	Inactive	CHEMBL4068410	2.881	Inactive	CHEMBL3586198	3.999	Inactive
CHEMBL3824057	3.895	Inactive	CHEMBL3824248	2.436	Inactive	CHEMBL4165299	3.947	Inactive
CHEMBL3414597	2.972	Inactive	CHEMBL4074655	2.300	Inactive	CHEMBL3585374	2.279	Inactive
CHEMBL3356535	2.013	Inactive	CHEMBL4205954	-1.878	Active	CHEMBL3586576	2.898	Inactive
CHEMBL3763203	3.009	Inactive	CHEMBL4214430	-1.777	Active	CHEMBL3335061	4.057	Inactive
CHEMBL3765162	3.686	Inactive	CHEMBL1289391	3.623	Inactive	CHEMBL3335055	3.940	Inactive
CHEMBL3754480	2.509	Inactive	CHEMBL1289719	3.881	Inactive	CHEMBL3335050	3.000	Inactive
CHEMBL3752467	-0.066	Active	CHEMBL3290193	4.822	Inactive	CHEMBL3335049	3.792	Inactive
CHEMBL3753405	0.408	Active	CHEMBL4087978	3.193	Inactive	CHEMBL3335046	3.633	Inactive
CHEMBL3338996	4.366	Inactive	CHEMBL3310706	4.670	Inactive	CHEMBL3335026	3.778	Inactive
CHEMBL3339006	4.341	Inactive	CHEMBL4216066	1.814	Active	CHEMBL2409141	4.083	Inactive
CHEMBL3356533	0.732	Active	CHEMBL3415643	2.431	Inactive	CHEMBL2396912	4.772	Inactive
CHEMBL3585779	-1.571	Active	CHEMBL4096963	3.568	Inactive	CHEMBL3586199	2.959	Inactive
CHEMBL3585782	-1.571	Active	CHEMBL4094981	3.262	Inactive	CHEMBL3971112	4.100	Inactive
CHEMBL3356539	2.542	Inactive	CHEMBL4059684	3.502	Inactive	CHEMBL3237632	4.838	Inactive
CHEMBL3754631	4.342	Inactive	CHEMBL4072405	3.288	Inactive	CHEMBL3289924	4.094	Inactive
CHEMBL3393674	4.342	Inactive	CHEMBL3818689	2.415	Inactive	CHEMBL3289942	4.899	Inactive
CHEMBL3323035	1.530	Active	CHEMBL4098654	1.799	Active	CHEMBL1327885	3.792	Inactive
CHEMBL3115039	3.942	Inactive	CHEMBL4205374	2.272	Inactive	CHEMBL2332977	3.614	Inactive
CHEMBL4278287	1.580	Active	CHEMBL4205426	0.725	Active	CHEMBL2047530	1.952	Active
CHEMBL3950130	2.991	Inactive	CHEMBL4203988	1.025	Active	CHEMBL4204901	2.885	Inactive
CHEMBL3752119	1.509	Active	CHEMBL4061837	3.881	Inactive	CHEMBL4206484	2.288	Inactive
CHEMBL3356531	1.730	Active	CHEMBL4077088	3.799	Inactive	CHEMBL2332980	4.298	Inactive
CHEMBL4167524	1.431	Active	CHEMBL1626455	3.903	Inactive	CHEMBL2332978	4.303	Inactive
CHEMBL4160310	2.965	Inactive	CHEMBL4160696	2.966	Inactive	CHEMBL4159705	0.431	Active
CHEMBL3597008	3.697	Inactive	CHEMBL3764065	3.804	Inactive	CHEMBL3818374	4.102	Inactive
CHEMBL4062800	2.230	Inactive	CHEMBL3763482	3.569	Inactive	CHEMBL4105158	3.134	Inactive
CHEMBL511921	2.310	Inactive	CHEMBL3765496	3.598	Inactive	CHEMBL4095695	4.288	Inactive
CHEMBL3948489	2.863	Inactive	CHEMBL4167958	4.763	Inactive	CHEMBL4095445	3.881	Inactive
CHEMBL3115026	3.533	Inactive	CHEMBL3093791	1.180	Active	CHEMBL3338995	4.352	Inactive
CHEMBL3115023	4.144	Inactive	CHEMBL3087675	3.980	Inactive	CHEMBL3769555	3.792	Inactive
CHEMBL4080216	4.904	Inactive	CHEMBL4289086	4.114	Inactive	CHEMBL4090751	2.600	Inactive
CHEMBL4077169	4.090	Inactive	CHEMBL3632987	2.415	Inactive	CHEMBL4129940	4.052	Inactive
CHEMBL4075624	4.223	Inactive	CHEMBL3632991	2.079	Inactive	CHEMBL2047231	4.013	Inactive
CHEMBL4080105	2.851	Inactive	CHEMBL3582203	3.613	Inactive	CHEMBL2047225	3.944	Inactive
CHEMBL4081013	3.919	Inactive	CHEMBL2332548	4.243	Inactive	CHEMBL1834062	4.279	Inactive
CHEMBL4083524	3.748	Inactive	CHEMBL3116298	3.934	Inactive	CHEMBL4072457	4.845	Inactive
CHEMBL4105088	3.892	Inactive	CHEMBL3133430	3.121	Inactive	CHEMBL23838	1.000	Active
CHEMBL4094911	3.929	Inactive	CHEMBL3415575	2.581	Inactive	CHEMBL4077952	1.301	Active
CHEMBL4096673	4.097	Inactive	CHEMBL1834067	3.991	Inactive	CHEMBL4060687	3.934	Inactive
CHEMBL4099790	3.833	Inactive	CHEMBL1834073	4.164	Inactive	CHEMBL4073193	3.857	Inactive
CHEMBL3919086	3.669	Inactive	CHEMBL3585778	-1.571	Active	CHEMBL4060016	3.869	Inactive

Table S1. (*Cont.*)

Molecule ChEMBL ID	logIC50 (nM)	Label	Molecule ChEMBL ID	logIC50 (nM)	Label	Molecule ChEMBL ID	logIC50 (nM)	Label
CHEMBL4173258	2.724	Inactive	CHEMBL3115045	3.788	Inactive	CHEMBL4282154	0.322	Active
CHEMBL4210949	3.491	Inactive	CHEMBL2297279	1.079	Active	CHEMBL4217660	2.255	Inactive
CHEMBL4218997	3.763	Inactive	CHEMBL2234843	3.260	Inactive	CHEMBL4215154	1.952	Active
CHEMBL4207876	3.813	Inactive	CHEMBL4240861	1.167	Active	CHEMBL4217617	3.185	Inactive
CHEMBL3955393	3.286	Inactive	CHEMBL3338386	2.996	Inactive	CHEMBL4212151	3.422	Inactive
CHEMBL4218829	1.487	Active	CHEMBL4082959	2.420	Inactive	CHEMBL4168710	3.790	Inactive
CHEMBL4208866	-1.547	Active	CHEMBL4165471	4.893	Inactive	CHEMBL4093408	4.365	Inactive
CHEMBL4128985	4.399	Inactive	CHEMBL4059887	3.778	Inactive	CHEMBL4086323	3.997	Inactive
CHEMBL4128061	3.767	Inactive	CHEMBL4203653	3.508	Inactive	CHEMBL4085283	4.570	Inactive
CHEMBL4277644	3.400	Inactive	CHEMBL4094805	3.121	Inactive	CHEMBL4093019	3.793	Inactive
CHEMBL4089082	2.041	Inactive	CHEMBL4069184	2.708	Inactive	CHEMBL4100736	3.464	Inactive
CHEMBL3582215	2.204	Inactive	CHEMBL4099046	3.940	Inactive	CHEMBL3338389	1.778	Active
CHEMBL3585784	-1.571	Active	CHEMBL4078382	3.875	Inactive	CHEMBL4209420	2.746	Inactive
CHEMBL2158994	3.670	Inactive	CHEMBL4177242	3.288	Inactive	CHEMBL4287826	3.534	Inactive
CHEMBL3338994	4.154	Inactive	CHEMBL4089264	2.771	Inactive	CHEMBL4284047	0.097	Active
CHEMBL3339001	3.772	Inactive	CHEMBL4172271	4.552	Inactive	CHEMBL4210274	4.871	Inactive
CHEMBL3600551	0.283	Active	CHEMBL3917990	3.600	Inactive	CHEMBL4217139	1.565	Active
CHEMBL3416996	1.839	Active	CHEMBL4287340	3.690	Inactive	CHEMBL4100338	3.602	Inactive
CHEMBL4216806	3.734	Inactive	CHEMBL4291697	3.250	Inactive	CHEMBL4092602	3.591	Inactive
CHEMBL4063663	4.386	Inactive	CHEMBL4164981	2.385	Inactive	CHEMBL4091651	3.756	Inactive
CHEMBL3585783	-1.571	Active	CHEMBL4293364	3.057	Inactive	CHEMBL4062146	3.820	Inactive
CHEMBL3115196	3.880	Inactive	CHEMBL4128040	4.292	Inactive	CHEMBL4097877	4.865	Inactive
CHEMBL4091851	2.778	Inactive	CHEMBL4286736	3.387	Inactive	CHEMBL1917825	3.342	Inactive
CHEMBL2419686	2.491	Inactive	CHEMBL4217663	-1.849	Active	CHEMBL4090159	3.866	Inactive
CHEMBL3582208	2.850	Inactive	CHEMBL4214707	-1.679	Active	CHEMBL4286806	3.735	Inactive
CHEMBL3582214	1.255	Active	CHEMBL4067562	4.278	Inactive	CHEMBL4282755	3.994	Inactive
CHEMBL3115034	3.645	Inactive	CHEMBL4209795	2.035	Inactive	CHEMBL3785861	2.930	Inactive
CHEMBL3115197	3.420	Inactive	CHEMBL4292766	2.875	Inactive	CHEMBL4204315	0.422	Active
CHEMBL4205651	2.723	Inactive	CHEMBL4284580	3.966	Inactive	CHEMBL4292349	3.539	Inactive
CHEMBL4287962	3.940	Inactive	CHEMBL4293626	3.507	Inactive	CHEMBL4175914	3.919	Inactive
CHEMBL4279297	2.322	Inactive	CHEMBL3234587	2.415	Inactive	CHEMBL4280241	3.517	Inactive
CHEMBL4289908	3.772	Inactive	CHEMBL3234593	2.176	Inactive	CHEMBL4074258	4.046	Inactive
CHEMBL4069385	3.854	Inactive	CHEMBL3765098	3.795	Inactive	CHEMBL4277879	3.862	Inactive
CHEMBL4098132	3.615	Inactive	CHEMBL3752384	2.346	Inactive	CHEMBL4285818	3.149	Inactive
CHEMBL3786719	1.568	Active	CHEMBL3754672	2.301	Inactive	CHEMBL4217346	1.974	Active
CHEMBL4293418	0.556	Active	CHEMBL1773484	2.029	Inactive	CHEMBL4214755	3.441	Inactive
CHEMBL4284475	2.991	Inactive	CHEMBL4213253	-1.723	Active	CHEMBL3891025	3.920	Inactive
CHEMBL3582231	3.016	Inactive	CHEMBL4209803	-1.869	Active	CHEMBL4281727	3.581	Inactive
CHEMBL4060800	3.643	Inactive	CHEMBL4205144	-1.586	Active	CHEMBL4063877	1.680	Active
CHEMBL2413558	4.370	Inactive	CHEMBL4099598	3.806	Inactive	CHEMBL4293155	3.387	Inactive
CHEMBL3945423	3.037	Inactive	CHEMBL4286556	3.567	Inactive	CHEMBL4214417	3.415	Inactive
CHEMBL4060832	4.614	Inactive	CHEMBL4278046	3.663	Inactive	CHEMBL4213042	0.352	Active
CHEMBL4098808	4.445	Inactive	CHEMBL4280753	4.940	Inactive	CHEMBL4208392	3.511	Inactive
CHEMBL3786448	1.199	Active	CHEMBL4284506	4.968	Inactive	CHEMBL4212229	3.861	Inactive
CHEMBL4076034	3.342	Inactive	CHEMBL4283463	3.931	Inactive	CHEMBL239400	3.903	Inactive
CHEMBL4060617	2.403	Inactive	CHEMBL4294859	3.549	Inactive	CHEMBL3765476	3.528	Inactive
CHEMBL4241840	1.644	Active	CHEMBL4085114	3.845	Inactive	CHEMBL4125722	3.856	Inactive
CHEMBL4102562	4.420	Inactive	CHEMBL2413733	4.091	Inactive	CHEMBL3819548	2.653	Inactive
CHEMBL3115036	3.788	Inactive	CHEMBL4098421	3.863	Inactive	CHEMBL4293305	2.763	Inactive
CHEMBL3115031	3.715	Inactive	CHEMBL456881	4.796	Inactive	CHEMBL4278260	3.444	Inactive

Table S1. (*Cont.*)

Molecule ChEMBL ID	$logIC_{50}$ (nM)	Label	Molecule ChEMBL ID	$logIC_{50}$ (nM)	Label	Molecule ChEMBL ID	$logIC_{50}$ (nM)	Label
CHEMBL4290039	1.342	Active	CHEMBL3752232	3.708	Inactive	CHEMBL4164524	2.185	Inactive
CHEMBL3983017	2.919	Inactive	CHEMBL4208869	1.382	Active	CHEMBL4285759	4.006	Inactive
CHEMBL3972865	3.356	Inactive	CHEMBL4276675	1.519	Active	CHEMBL4172510	3.606	Inactive
CHEMBL3958208	3.170	Inactive	CHEMBL4171637	4.986	Inactive	CHEMBL4216666	3.362	Inactive
CHEMBL4127747	4.037	Inactive	CHEMBL4202749	1.225	Active	CHEMBL4064904	3.633	Inactive
CHEMBL4092562	3.137	Inactive	CHEMBL4285581	1.362	Active	CHEMBL4290877	3.346	Inactive
CHEMBL4068935	3.097	Inactive	CHEMBL3417009	1.204	Active	CHEMBL4166300	1.146	Active
CHEMBL4283814	3.708	Inactive	CHEMBL4278164	2.114	Inactive	CHEMBL4164078	2.785	Inactive
CHEMBL4164540	2.949	Inactive	CHEMBL4208322	1.326	Active	CHEMBL4174290	4.029	Inactive
CHEMBL4077157	2.360	Inactive	CHEMBL4207386	1.413	Active	CHEMBL4159640	3.064	Inactive
CHEMBL4174729	2.322	Inactive	CHEMBL4284619	4.255	Inactive	CHEMBL4101303	3.727	Inactive
CHEMBL4283189	3.648	Inactive	CHEMBL4276694	4.279	Inactive	CHEMBL4159074	3.924	Inactive
CHEMBL4291046	2.934	Inactive	CHEMBL4217746	1.403	Active	CHEMBL4278686	0.806	Active
CHEMBL4279249	3.777	Inactive	CHEMBL4283390	3.348	Inactive	CHEMBL4283651	3.550	Inactive
CHEMBL4287595	2.959	Inactive	CHEMBL4225157	0.000	Active	CHEMBL4279488	3.140	Inactive
CHEMBL4295126	3.124	Inactive	CHEMBL4205425	-1.961	Active	CHEMBL4290236	3.520	Inactive
CHEMBL4282414	3.786	Inactive	CHEMBL4243474	1.474	Active	CHEMBL4216601	3.978	Inactive
CHEMBL4214668	2.111	Inactive	CHEMBL4213792	-0.292	Active	CHEMBL709	1.255	Active
CHEMBL3921061	2.940	Inactive	CHEMBL4210041	-1.666	Active	CHEMBL4284895	3.303	Inactive
CHEMBL4289362	2.771	Inactive	CHEMBL3787502	1.350	Active	CHEMBL4217126	3.350	Inactive
CHEMBL4277357	4.756	Inactive	CHEMBL4292192	4.807	Inactive	CHEMBL4159609	2.462	Inactive
CHEMBL4168299	4.452	Inactive	CHEMBL3965783	2.996	Inactive	CHEMBL4291400	4.301	Inactive
CHEMBL4215217	1.872	Active	CHEMBL4210517	2.076	Inactive	CHEMBL4282037	3.086	Inactive
CHEMBL4294640	-0.022	Active	CHEMBL85251	2.605	Inactive	CHEMBL4285972	4.000	Inactive
CHEMBL4288282	3.326	Inactive	CHEMBL4281066	3.210	Inactive			
CHEMBL4163781	4.999	Inactive	CHEMBL4244803	1.326	Active			

Table S2. Molecule ChEMBL ID (https://www.ebi.ac.uk/chembl/) and labels of 1549 compounds in BACE1 database

Molecule ChEMBL ID	$logIC_{50}$ (nM)	Label	Molecule ChEMBL ID	$logIC_{50}$ (nM)	Label	Molecule ChEMBL ID	$logIC_{50}$ (nM)	Label
CHEMBL2181911	0.740	Active	CHEMBL2047892	2.114	Inactive	CHEMBL3753957	2.279	Inactive
CHEMBL2181890	0.982	Active	CHEMBL509486	4.566	Inactive	CHEMBL208060	0.806	Active
CHEMBL2177328	3.441	Inactive	CHEMBL2380459	3.505	Inactive	CHEMBL1762553	3.915	Inactive
CHEMBL2177330	3.193	Inactive	CHEMBL2380446	2.799	Inactive	CHEMBL1957479	3.530	Inactive
CHEMBL2177332	2.954	Inactive	CHEMBL583888	2.322	Inactive	CHEMBL1957480	1.890	Active
CHEMBL2177333	2.869	Inactive	CHEMBL567653	3.914	Inactive	CHEMBL454662	3.217	Inactive
CHEMBL1821816	2.987	Inactive	CHEMBL584521	2.000	Inactive	CHEMBL1923294	2.910	Inactive
CHEMBL1821817	1.204	Active	CHEMBL582044	4.580	Inactive	CHEMBL3640259	1.580	Active
CHEMBL1821728	-0.155	Active	CHEMBL598876	2.114	Inactive	CHEMBL3640274	2.057	Inactive
CHEMBL1821826	1.204	Active	CHEMBL2403772	1.491	Active	CHEMBL3261082	1.362	Active
CHEMBL2177916	2.130	Inactive	CHEMBL2181914	0.991	Active	CHEMBL3645153	2.230	Inactive
CHEMBL2177905	2.137	Inactive	CHEMBL2181831	2.158	Inactive	CHEMBL2177479	3.342	Inactive
CHEMBL2177321	3.846	Inactive	CHEMBL2314791	3.690	Inactive	CHEMBL28	4.585	Inactive
CHEMBL3261047	1.301	Active	CHEMBL378225	1.299	Active	CHEMBL1412710	4.310	Inactive
CHEMBL3261048	1.919	Active	CHEMBL1934195	3.996	Inactive	CHEMBL2181979	4.933	Inactive
CHEMBL190644	1.987	Active	CHEMBL2380821	0.903	Active	CHEMBL2172798	4.533	Inactive
CHEMBL2047888	2.544	Inactive	CHEMBL332948	1.322	Active	CHEMBL2172796	4.146	Inactive

Table S2. (*Cont.*)

Molecule ChEMBL ID	logIC$_{50}$ (nM)	Label	Molecule ChEMBL ID	logIC$_{50}$ (nM)	Label	Molecule ChEMBL ID	logIC$_{50}$ (nM)	Label
CHEMBL2169944	4.771	Inactive	CHEMBL2347187	1.279	Active	CHEMBL3747766	2.079	Inactive
CHEMBL2181910	0.756	Active	CHEMBL2347207	2.890	Inactive	CHEMBL217068	3.568	Inactive
CHEMBL2177908	2.297	Inactive	CHEMBL1957481	1.750	Active	CHEMBL585506	1.602	Active
CHEMBL51386	2.477	Inactive	CHEMBL1921816	2.740	Inactive	CHEMBL1099022	3.422	Inactive
CHEMBL1762678	4.367	Inactive	CHEMBL3086247	3.716	Inactive	CHEMBL3746131	3.498	Inactive
CHEMBL2177331	2.447	Inactive	CHEMBL3086244	4.173	Inactive	CHEMBL597233	3.428	Inactive
CHEMBL2177340	2.029	Inactive	CHEMBL2403759	3.305	Inactive	CHEMBL3261044	1.505	Active
CHEMBL2180031	1.415	Active	CHEMBL2403765	1.176	Active	CHEMBL3260840	1.886	Active
CHEMBL2177912	1.631	Active	CHEMBL3086243	4.155	Inactive	CHEMBL2177917	1.895	Active
CHEMBL2048058	0.301	Active	CHEMBL2177325	2.114	Inactive	CHEMBL1080086	3.681	Inactive
CHEMBL2048059	0.301	Active	CHEMBL2403768	1.591	Active	CHEMBL3640220	2.230	Inactive
CHEMBL332260	1.699	Active	CHEMBL2403767	1.505	Active	CHEMBL3640240	2.940	Inactive
CHEMBL2180034	3.146	Inactive	CHEMBL2380811	2.914	Inactive	CHEMBL191344	1.398	Active
CHEMBL2180018	3.544	Inactive	CHEMBL3414708	2.929	Inactive	CHEMBL208808	1.716	Active
CHEMBL66	4.477	Inactive	CHEMBL2177327	3.704	Inactive	CHEMBL3597552	3.507	Inactive
CHEMBL370955	1.813	Active	CHEMBL2177310	3.290	Inactive	CHEMBL3359760	0.672	Active
CHEMBL370185	3.692	Inactive	CHEMBL2181881	1.310	Active	CHEMBL403268	-0.398	Active
CHEMBL205309	1.968	Active	CHEMBL3359755	-0.046	Active	CHEMBL3359761	0.663	Active
CHEMBL3813928	2.839	Inactive	CHEMBL3753360	2.792	Inactive	CHEMBL3359758	-0.097	Active
CHEMBL567430	1.477	Active	CHEMBL1760733	3.362	Inactive	CHEMBL2177312	3.873	Inactive
CHEMBL3765711	2.863	Inactive	CHEMBL1760859	2.477	Inactive	CHEMBL3746656	2.863	Inactive
CHEMBL3765050	2.591	Inactive	CHEMBL1821812	1.531	Active	CHEMBL3354693	0.602	Active
CHEMBL3763815	3.358	Inactive	CHEMBL1821825	1.000	Active	CHEMBL3354696	0.845	Active
CHEMBL3640279	2.246	Inactive	CHEMBL3640253	2.158	Inactive	CHEMBL3354698	0.602	Active
CHEMBL252962	3.900	Inactive	CHEMBL3640280	1.934	Active	CHEMBL3354702	1.362	Active
CHEMBL401141	2.775	Inactive	CHEMBL3645154	2.857	Inactive	CHEMBL2396989	1.301	Active
CHEMBL373533	0.699	Active	CHEMBL3640231	3.394	Inactive	CHEMBL3959813	2.204	Inactive
CHEMBL379334	3.097	Inactive	CHEMBL3640269	2.465	Inactive	CHEMBL3640243	1.000	Active
CHEMBL213904	1.898	Active	CHEMBL3747191	2.643	Inactive	CHEMBL3640250	3.775	Inactive
CHEMBL1957468	1.950	Active	CHEMBL2181885	1.260	Active	CHEMBL3640227	3.330	Inactive
CHEMBL1957478	1.570	Active	CHEMBL3354692	0.845	Active	CHEMBL3640234	1.903	Active
CHEMBL1957483	1.500	Active	CHEMBL566969	2.869	Inactive	CHEMBL2347214	1.820	Active
CHEMBL291784	3.537	Inactive	CHEMBL566112	1.903	Active	CHEMBL3359753	0.079	Active
CHEMBL566406	1.699	Active	CHEMBL1762557	3.953	Inactive	CHEMBL3752467	3.130	Inactive
CHEMBL567061	1.699	Active	CHEMBL450745	3.491	Inactive	CHEMBL3354688	0.342	Active
CHEMBL1087609	3.841	Inactive	CHEMBL3422246	0.845	Active	CHEMBL2380440	4.505	Inactive
CHEMBL589614	2.322	Inactive	CHEMBL3414701	1.785	Active	CHEMBL2380457	4.301	Inactive
CHEMBL508791	3.344	Inactive	CHEMBL3394044	1.267	Active	CHEMBL2380447	3.505	Inactive
CHEMBL3086251	3.690	Inactive	CHEMBL566011	1.301	Active	CHEMBL2380442	4.000	Inactive
CHEMBL2177304	0.784	Active	CHEMBL1092786	1.602	Active	CHEMBL2380451	2.505	Inactive
CHEMBL2177335	2.903	Inactive	CHEMBL597277	1.477	Active	CHEMBL3265337	3.886	Inactive
CHEMBL2177317	1.959	Active	CHEMBL605079	3.681	Inactive	CHEMBL183494	0.914	Active
CHEMBL2347203	1.708	Active	CHEMBL565995	2.114	Inactive	CHEMBL202602	1.991	Active
CHEMBL3653370	2.230	Inactive	CHEMBL567258	1.778	Active	CHEMBL565996	1.556	Active
CHEMBL584926	1.000	Active	CHEMBL2347368	2.033	Inactive	CHEMBL567687	1.778	Active
CHEMBL2177914	1.559	Active	CHEMBL2181829	1.358	Active	CHEMBL566404	3.134	Inactive
CHEMBL2181886	1.547	Active	CHEMBL592534	3.017	Inactive	CHEMBL2047908	1.447	Active
CHEMBL2180035	3.477	Inactive	CHEMBL566415	2.398	Inactive	CHEMBL3394046	0.114	Active
CHEMBL2181907	0.462	Active	CHEMBL116438	3.724	Inactive	CHEMBL2403774	1.785	Active
CHEMBL2136098	4.435	Inactive	CHEMBL3747381	2.857	Inactive	CHEMBL1923160	2.511	Inactive

Table S2. (*Cont.*)

Molecule ChEMBL ID	logIC$_{50}$ (nM)	Label	Molecule ChEMBL ID	logIC$_{50}$ (nM)	Label	Molecule ChEMBL ID	logIC$_{50}$ (nM)	Label
CHEMBL2180033	2.491	Inactive	CHEMBL1270434	1.954	Active	CHEMBL126079	3.255	Inactive
CHEMBL209795	2.079	Inactive	CHEMBL1270527	1.301	Active	CHEMBL189901	1.079	Active
CHEMBL210414	1.301	Active	CHEMBL1270729	1.699	Active	CHEMBL363783	1.602	Active
CHEMBL211563	0.602	Active	CHEMBL1271142	1.602	Active	CHEMBL191129	1.000	Active
CHEMBL257735	3.130	Inactive	CHEMBL1271246	1.398	Active	CHEMBL384678	2.068	Inactive
CHEMBL208681	3.294	Inactive	CHEMBL1271452	1.301	Active	CHEMBL211194	4.079	Inactive
CHEMBL213595	4.307	Inactive	CHEMBL1270028	2.079	Inactive	CHEMBL379781	1.114	Active
CHEMBL404078	0.778	Active	CHEMBL1270240	1.301	Active	CHEMBL1270934	1.301	Active
CHEMBL256676	3.301	Inactive	CHEMBL1270338	1.477	Active	CHEMBL270248	2.179	Inactive
CHEMBL256434	1.813	Active	CHEMBL1270334	1.114	Active	CHEMBL411917	2.763	Inactive
CHEMBL248228	1.079	Active	CHEMBL1270335	1.380	Active	CHEMBL1270780	3.230	Inactive
CHEMBL2177486	4.997	Inactive	CHEMBL1270630	1.176	Active	CHEMBL1269608	3.447	Inactive
CHEMBL401587	2.041	Inactive	CHEMBL1270631	1.301	Active	CHEMBL584695	2.748	Inactive
CHEMBL566978	1.903	Active	CHEMBL1270835	1.146	Active	CHEMBL3604595	4.415	Inactive
CHEMBL584288	1.778	Active	CHEMBL190010	1.778	Active	CHEMBL3747445	2.806	Inactive
CHEMBL566548	2.279	Inactive	CHEMBL2403773	1.982	Active	CHEMBL1099023	3.581	Inactive
CHEMBL568960	2.114	Inactive	CHEMBL2440151	3.668	Inactive	CHEMBL585952	3.185	Inactive
CHEMBL598259	1.477	Active	CHEMBL2443380	3.630	Inactive	CHEMBL584694	2.447	Inactive
CHEMBL597073	2.477	Inactive	CHEMBL3394211	-0.523	Active	CHEMBL566200	1.602	Active
CHEMBL598290	1.000	Active	CHEMBL3394215	0.079	Active	CHEMBL567688	1.301	Active
CHEMBL1270336	1.903	Active	CHEMBL3394218	-0.699	Active	CHEMBL1651460	1.301	Active
CHEMBL585128	1.845	Active	CHEMBL2403770	1.653	Active	CHEMBL604590	1.778	Active
CHEMBL1270239	1.903	Active	CHEMBL2322830	4.801	Inactive	CHEMBL599965	2.996	Inactive
CHEMBL584917	1.954	Active	CHEMBL191122	1.591	Active	CHEMBL399840	1.982	Active
CHEMBL2048061	1.643	Active	CHEMBL3354701	0.845	Active	CHEMBL566012	1.477	Active
CHEMBL260722	3.494	Inactive	CHEMBL2403761	3.009	Inactive	CHEMBL571320	2.230	Inactive
CHEMBL577905	3.491	Inactive	CHEMBL2380819	2.881	Inactive	CHEMBL324109	2.663	Inactive
CHEMBL1271192	3.452	Inactive	CHEMBL2058867	3.876	Inactive	CHEMBL2181901	0.785	Active
CHEMBL1269610	4.255	Inactive	CHEMBL2058875	3.829	Inactive	CHEMBL2181893	0.716	Active
CHEMBL254626	3.613	Inactive	CHEMBL255372	2.272	Inactive	CHEMBL595302	3.455	Inactive
CHEMBL1923296	3.850	Inactive	CHEMBL1271451	3.152	Inactive	CHEMBL211499	3.954	Inactive
CHEMBL559870	3.728	Inactive	CHEMBL1821821	1.301	Active	CHEMBL401008	1.380	Active
CHEMBL288919	1.740	Active	CHEMBL214963	3.041	Inactive	CHEMBL399113	1.826	Active
CHEMBL3394041	-0.301	Active	CHEMBL2380438	4.398	Inactive	CHEMBL578410	3.398	Inactive
CHEMBL3394045	0.342	Active	CHEMBL2380456	2.699	Inactive	CHEMBL210451	4.000	Inactive
CHEMBL3394050	0.431	Active	CHEMBL381618	3.230	Inactive	CHEMBL270359	0.699	Active
CHEMBL3422237	0.477	Active	CHEMBL206665	3.279	Inactive	CHEMBL404680	1.949	Active
CHEMBL584284	2.431	Inactive	CHEMBL2380822	2.609	Inactive	CHEMBL271710	2.057	Inactive
CHEMBL598257	2.000	Inactive	CHEMBL1098682	4.291	Inactive	CHEMBL402330	2.778	Inactive
CHEMBL590730	1.477	Active	CHEMBL1099021	3.579	Inactive	CHEMBL1957474	1.370	Active
CHEMBL129451	3.778	Inactive	CHEMBL1821811	1.869	Active	CHEMBL1957470	1.610	Active
CHEMBL1270432	1.301	Active	CHEMBL210240	2.279	Inactive	CHEMBL2443369	0.477	Active
CHEMBL1270526	1.079	Active	CHEMBL1923295	3.140	Inactive	CHEMBL43962	3.146	Inactive
CHEMBL1760852	3.447	Inactive	CHEMBL370369	2.380	Inactive	CHEMBL2048040	1.041	Active
CHEMBL1760864	3.633	Inactive	CHEMBL3414710	2.447	Inactive	CHEMBL2048043	1.633	Active
CHEMBL1821819	1.041	Active	CHEMBL3414704	4.944	Inactive	CHEMBL2048045	0.778	Active
CHEMBL1821827	1.041	Active	CHEMBL116826	4.531	Inactive	CHEMBL437961	3.717	Inactive
CHEMBL202015	3.740	Inactive	CHEMBL2380820	2.708	Inactive	CHEMBL378897	3.114	Inactive
CHEMBL381617	3.740	Inactive	CHEMBL295109	3.820	Inactive	CHEMBL1760853	3.792	Inactive
CHEMBL1270133	2.041	Inactive	CHEMBL585286	2.940	Inactive	CHEMBL403819	2.380	Inactive

Table S2. (*Cont.*)

Molecule ChEMBL ID	logIC$_{50}$ (nM)	Label	Molecule ChEMBL ID	logIC$_{50}$ (nM)	Label	Molecule ChEMBL ID	logIC$_{50}$ (nM)	Label
CHEMBL254625	2.491	Inactive	CHEMBL2152903	0.387	Active	CHEMBL428074	2.643	Inactive
CHEMBL1762552	3.970	Inactive	CHEMBL1270027	1.954	Active	CHEMBL200510	3.380	Inactive
CHEMBL1762556	4.050	Inactive	CHEMBL1270433	1.477	Active	CHEMBL2059593	3.918	Inactive
CHEMBL1762679	4.058	Inactive	CHEMBL1270628	1.301	Active	CHEMBL2169946	4.705	Inactive
CHEMBL1762685	4.379	Inactive	CHEMBL1271037	1.000	Active	CHEMBL384496	1.505	Active
CHEMBL181826	0.672	Active	CHEMBL379820	1.833	Active	CHEMBL583213	4.480	Inactive
CHEMBL2059330	3.876	Inactive	CHEMBL377581	2.041	Inactive	CHEMBL565251	4.307	Inactive
CHEMBL453211	0.954	Active	CHEMBL1087736	4.197	Inactive	CHEMBL2407341	2.079	Inactive
CHEMBL504917	1.380	Active	CHEMBL1270529	1.477	Active	CHEMBL2180025	2.431	Inactive
CHEMBL1821818	1.431	Active	CHEMBL1270834	1.447	Active	CHEMBL2380814	3.037	Inactive
CHEMBL566977	3.724	Inactive	CHEMBL1957477	1.460	Active	CHEMBL3747247	2.857	Inactive
CHEMBL567654	3.415	Inactive	CHEMBL1760863	4.820	Inactive	CHEMBL585215	3.093	Inactive
CHEMBL582045	1.531	Active	CHEMBL3359748	-0.222	Active	CHEMBL378052	1.839	Active
CHEMBL221454	1.176	Active	CHEMBL212578	2.179	Inactive	CHEMBL3086250	3.875	Inactive
CHEMBL222209	1.799	Active	CHEMBL585120	4.653	Inactive	CHEMBL2177904	2.093	Inactive
CHEMBL3354708	0.602	Active	CHEMBL3327246	4.305	Inactive	CHEMBL2177336	2.954	Inactive
CHEMBL3354714	1.851	Active	CHEMBL253165	2.685	Inactive	CHEMBL1270884	2.079	Inactive
CHEMBL3354690	0.491	Active	CHEMBL257087	2.799	Inactive	CHEMBL1270082	4.699	Inactive
CHEMBL3354705	0.778	Active	CHEMBL42453	1.699	Active	CHEMBL2407342	1.000	Active
CHEMBL211432	4.228	Inactive	CHEMBL605133	1.000	Active	CHEMBL566632	3.143	Inactive
CHEMBL3414707	2.845	Inactive	CHEMBL605518	2.580	Inactive	CHEMBL1099361	3.364	Inactive
CHEMBL212203	2.143	Inactive	CHEMBL1269919	2.954	Inactive	CHEMBL201802	3.771	Inactive
CHEMBL209372	0.845	Active	CHEMBL1270238	1.699	Active	CHEMBL202057	2.653	Inactive
CHEMBL3394226	-0.699	Active	CHEMBL1270833	2.415	Inactive	CHEMBL3354710	-0.301	Active
CHEMBL324122	4.623	Inactive	CHEMBL1270935	1.602	Active	CHEMBL456228	3.934	Inactive
CHEMBL364664	3.980	Inactive	CHEMBL1270436	1.477	Active	CHEMBL1821829	2.591	Inactive
CHEMBL248056	1.591	Active	CHEMBL1088138	3.907	Inactive	CHEMBL381826	0.681	Active
CHEMBL1083394	4.067	Inactive	CHEMBL261655	4.153	Inactive	CHEMBL272320	2.886	Inactive
CHEMBL211097	3.580	Inactive	CHEMBL590829	2.000	Inactive	CHEMBL3422238	0.602	Active
CHEMBL419949	3.748	Inactive	CHEMBL589891	2.591	Inactive	CHEMBL3414705	4.236	Inactive
CHEMBL183059	1.924	Active	CHEMBL455667	4.593	Inactive	CHEMBL1270936	1.613	Active
CHEMBL2047890	2.146	Inactive	CHEMBL455679	3.415	Inactive	CHEMBL3394056	0.380	Active
CHEMBL2047898	1.740	Active	CHEMBL260634	3.759	Inactive	CHEMBL3604698	4.857	Inactive
CHEMBL2047912	1.690	Active	CHEMBL380257	2.799	Inactive	CHEMBL607527	2.978	Inactive
CHEMBL2048041	1.398	Active	CHEMBL338988	3.204	Inactive	CHEMBL3236216	3.848	Inactive
CHEMBL257278	0.903	Active	CHEMBL1760866	3.146	Inactive	CHEMBL1270883	3.248	Inactive
CHEMBL257907	2.519	Inactive	CHEMBL200617	2.415	Inactive	CHEMBL1269866	4.699	Inactive
CHEMBL114147	3.954	Inactive	CHEMBL2059601	3.693	Inactive	CHEMBL253800	2.049	Inactive
CHEMBL2048054	0.602	Active	CHEMBL2058871	4.196	Inactive	CHEMBL410865	3.747	Inactive
CHEMBL1927636	2.216	Inactive	CHEMBL2048046	1.875	Active	CHEMBL2346798	3.433	Inactive
CHEMBL1927635	1.658	Active	CHEMBL2048049	1.633	Active	CHEMBL3394213	-0.398	Active
CHEMBL260834	2.633	Inactive	CHEMBL206442	2.881	Inactive	CHEMBL3394214	-0.097	Active
CHEMBL2314782	4.544	Inactive	CHEMBL1271345	2.519	Inactive	CHEMBL3394216	-0.523	Active
CHEMBL211214	2.799	Inactive	CHEMBL248223	2.770	Inactive	CHEMBL205333	0.820	Active
CHEMBL399979	0.845	Active	CHEMBL2425604	2.326	Inactive	CHEMBL2177303	0.413	Active
CHEMBL2407492	0.431	Active	CHEMBL2425600	1.000	Active	CHEMBL2177313	4.057	Inactive
CHEMBL255838	0.748	Active	CHEMBL259547	4.091	Inactive	CHEMBL585289	1.602	Active
CHEMBL1092476	1.699	Active	CHEMBL253237	2.204	Inactive	CHEMBL164	3.447	Inactive
CHEMBL603664	2.176	Inactive	CHEMBL2181913	1.204	Active	CHEMBL1090026	1.845	Active
CHEMBL592533	3.033	Inactive	CHEMBL263260	2.602	Inactive	CHEMBL1080295	3.380	Inactive

Table S2. (*Cont.*)

Molecule ChEMBL ID	$logIC_{50}$ (nM)	Label	Molecule ChEMBL ID	$logIC_{50}$ (nM)	Label	Molecule ChEMBL ID	$logIC_{50}$ (nM)	Label
CHEMBL576258	1.301	Active	CHEMBL566414	2.079	Inactive	CHEMBL566212	2.954	Inactive
CHEMBL3354718	-0.523	Active	CHEMBL206097	3.415	Inactive	CHEMBL1092787	1.301	Active
CHEMBL2177315	3.455	Inactive	CHEMBL205637	3.053	Inactive	CHEMBL212102	3.049	Inactive
CHEMBL2177342	1.204	Active	CHEMBL398925	3.565	Inactive	CHEMBL377548	2.708	Inactive
CHEMBL3640252	2.336	Inactive	CHEMBL206854	3.380	Inactive	CHEMBL255194	1.146	Active
CHEMBL3640224	2.000	Inactive	CHEMBL256372	1.415	Active	CHEMBL1821823	1.491	Active
CHEMBL3640246	2.301	Inactive	CHEMBL258083	1.845	Active	CHEMBL2172800	4.041	Inactive
CHEMBL3645150	2.000	Inactive	CHEMBL81671	1.914	Active	CHEMBL2169942	4.149	Inactive
CHEMBL3645156	2.204	Inactive	CHEMBL3765195	3.000	Inactive	CHEMBL2181897	1.176	Active
CHEMBL1092124	1.477	Active	CHEMBL3765319	3.781	Inactive	CHEMBL2172799	4.365	Inactive
CHEMBL3414700	2.826	Inactive	CHEMBL3763728	1.602	Active	CHEMBL2047887	1.740	Active
CHEMBL78946	0.845	Active	CHEMBL209052	3.000	Inactive	CHEMBL2047891	3.137	Inactive
CHEMBL3764532	2.602	Inactive	CHEMBL211256	1.954	Active	CHEMBL2059613	3.823	Inactive
CHEMBL3764465	3.017	Inactive	CHEMBL426717	3.146	Inactive	CHEMBL2059624	3.982	Inactive
CHEMBL4073296	4.611	Inactive	CHEMBL1821828	1.987	Active	CHEMBL2059626	4.029	Inactive
CHEMBL1270026	1.954	Active	CHEMBL403727	0.477	Active	CHEMBL2059100	4.004	Inactive
CHEMBL1271245	1.301	Active	CHEMBL2347209	2.137	Inactive	CHEMBL2346804	2.771	Inactive
CHEMBL1270339	1.845	Active	CHEMBL309438	2.813	Inactive	CHEMBL2346802	2.672	Inactive
CHEMBL2180037	1.898	Active	CHEMBL2047914	2.826	Inactive	CHEMBL2322541	4.770	Inactive
CHEMBL2180029	3.176	Inactive	CHEMBL2048050	0.477	Active	CHEMBL2322535	4.887	Inactive
CHEMBL3640242	1.000	Active	CHEMBL2048051	0.301	Active	CHEMBL2172804	4.314	Inactive
CHEMBL3640221	1.903	Active	CHEMBL2048052	1.505	Active	CHEMBL2172802	3.959	Inactive
CHEMBL3640276	1.342	Active	CHEMBL2048053	0.301	Active	CHEMBL2172794	4.155	Inactive
CHEMBL3400755	3.417	Inactive	CHEMBL2058869	3.902	Inactive	CHEMBL2169937	4.408	Inactive
CHEMBL3645147	1.903	Active	CHEMBL1271091	2.839	Inactive	CHEMBL2181985	4.405	Inactive
CHEMBL3640228	2.041	Inactive	CHEMBL1270985	3.477	Inactive	CHEMBL1957471	1.310	Active
CHEMBL3236224	3.791	Inactive	CHEMBL1269613	4.167	Inactive	CHEMBL1957475	1.140	Active
CHEMBL602233	4.210	Inactive	CHEMBL566223	1.699	Active	CHEMBL2181898	1.462	Active
CHEMBL2425602	1.114	Active	CHEMBL3236215	3.267	Inactive	CHEMBL2177490	4.049	Inactive
CHEMBL2425609	1.944	Active	CHEMBL1762558	4.013	Inactive	CHEMBL2380444	3.799	Inactive
CHEMBL126142	3.653	Inactive	CHEMBL1762687	4.301	Inactive	CHEMBL213301	4.301	Inactive
CHEMBL3642610	3.748	Inactive	CHEMBL567633	2.898	Inactive	CHEMBL4085945	3.845	Inactive
CHEMBL3642607	2.173	Inactive	CHEMBL28626	4.336	Inactive	CHEMBL1762689	4.053	Inactive
CHEMBL200195	2.455	Inactive	CHEMBL2181884	1.521	Active	CHEMBL1762686	4.084	Inactive
CHEMBL183546	0.591	Active	CHEMBL2169924	4.777	Inactive	CHEMBL1762683	4.203	Inactive
CHEMBL3659646	4.079	Inactive	CHEMBL2169922	4.389	Inactive	CHEMBL1821824	1.699	Active
CHEMBL3394223	-0.155	Active	CHEMBL2169939	4.233	Inactive	CHEMBL3659642	4.230	Inactive
CHEMBL1821815	0.398	Active	CHEMBL2177483	3.041	Inactive	CHEMBL4078371	2.060	Inactive
CHEMBL1821822	1.944	Active	CHEMBL2177481	3.806	Inactive	CHEMBL3653377	1.748	Active
CHEMBL1762681	4.127	Inactive	CHEMBL2177473	3.380	Inactive	CHEMBL4129775	1.447	Active
CHEMBL1099025	3.627	Inactive	CHEMBL507541	1.000	Active	CHEMBL4177357	2.778	Inactive
CHEMBL32823	3.875	Inactive	CHEMBL3642605	3.708	Inactive	CHEMBL393501	4.462	Inactive
CHEMBL3645173	2.638	Inactive	CHEMBL1269920	1.301	Active	CHEMBL235754	3.771	Inactive
CHEMBL1092475	1.602	Active	CHEMBL3642623	2.146	Inactive	CHEMBL567485	2.431	Inactive
CHEMBL3645148	2.230	Inactive	CHEMBL2347188	1.114	Active	CHEMBL453805	4.484	Inactive
CHEMBL3640281	1.954	Active	CHEMBL566403	1.954	Active	CHEMBL3640225	2.255	Inactive
CHEMBL3642634	3.929	Inactive	CHEMBL373255	3.987	Inactive	CHEMBL3640248	3.686	Inactive
CHEMBL367139	1.204	Active	CHEMBL26125	2.176	Inactive	CHEMBL3640232	3.831	Inactive
CHEMBL3261066	2.547	Inactive	CHEMBL272700	2.173	Inactive	CHEMBL4127486	4.130	Inactive
CHEMBL4126351	1.204	Active	CHEMBL2407494	1.881	Active	CHEMBL3653399	1.301	Active

Table S2. (*Cont.*)

Molecule ChEMBL ID	logIC$_{50}$ (nM)	Label	Molecule ChEMBL ID	logIC$_{50}$ (nM)	Label	Molecule ChEMBL ID	logIC$_{50}$ (nM)	Label
CHEMBL3086248	3.964	Inactive	CHEMBL201940	3.666	Inactive	CHEMBL2347195	1.431	Active
CHEMBL4127807	1.580	Active	CHEMBL3394055	0.398	Active	CHEMBL2347191	1.623	Active
CHEMBL205217	0.806	Active	CHEMBL383772	2.255	Inactive	CHEMBL257645	0.602	Active
CHEMBL439521	0.079	Active	CHEMBL201961	2.991	Inactive	CHEMBL256904	2.281	Inactive
CHEMBL380521	3.716	Inactive	CHEMBL248245	2.604	Inactive	CHEMBL270153	2.114	Inactive
CHEMBL4169090	3.342	Inactive	CHEMBL252547	3.616	Inactive	CHEMBL3265331	2.176	Inactive
CHEMBL2347199	2.649	Inactive	CHEMBL1269921	1.000	Active	CHEMBL3265334	1.778	Active
CHEMBL3394210	-0.155	Active	CHEMBL377624	4.236	Inactive	CHEMBL3265336	4.204	Inactive
CHEMBL3394228	-0.523	Active	CHEMBL211899	1.114	Active	CHEMBL201155	2.623	Inactive
CHEMBL2181980	4.086	Inactive	CHEMBL379513	0.903	Active	CHEMBL192836	2.267	Inactive
CHEMBL3236228	4.062	Inactive	CHEMBL3597550	3.915	Inactive	CHEMBL190058	3.260	Inactive
CHEMBL211384	2.000	Inactive	CHEMBL401527	2.079	Inactive	CHEMBL2380808	3.978	Inactive
CHEMBL2048057	1.833	Active	CHEMBL252961	3.037	Inactive	CHEMBL3763264	3.033	Inactive
CHEMBL2048063	0.845	Active	CHEMBL201730	1.544	Active	CHEMBL2181018	1.892	Active
CHEMBL2059101	2.851	Inactive	CHEMBL566407	1.699	Active	CHEMBL221597	1.301	Active
CHEMBL3640235	1.778	Active	CHEMBL439671	3.537	Inactive	CHEMBL3354694	1.230	Active
CHEMBL3640239	1.301	Active	CHEMBL187765	2.857	Inactive	CHEMBL3354700	0.954	Active
CHEMBL3640241	2.708	Inactive	CHEMBL568114	2.176	Inactive	CHEMBL598258	1.903	Active
CHEMBL3640261	2.328	Inactive	CHEMBL565378	2.799	Inactive	CHEMBL402276	1.079	Active
CHEMBL2314779	3.613	Inactive	CHEMBL297453	2.879	Inactive	CHEMBL584109	2.447	Inactive
CHEMBL2314793	3.415	Inactive	CHEMBL1271453	1.000	Active	CHEMBL2380817	2.756	Inactive
CHEMBL1269609	4.236	Inactive	CHEMBL1271346	1.301	Active	CHEMBL566633	2.505	Inactive
CHEMBL1762555	4.207	Inactive	CHEMBL1270029	1.000	Active	CHEMBL3260839	-0.699	Active
CHEMBL2177913	1.545	Active	CHEMBL1270431	1.903	Active	CHEMBL3261049	1.716	Active
CHEMBL2177907	2.403	Inactive	CHEMBL1090027	1.477	Active	CHEMBL3261078	0.000	Active
CHEMBL1091463	1.778	Active	CHEMBL1923158	2.650	Inactive	CHEMBL3261051	1.431	Active
CHEMBL3642619	2.857	Inactive	CHEMBL583608	2.204	Inactive	CHEMBL6246	3.591	Inactive
CHEMBL3645159	2.279	Inactive	CHEMBL583887	3.155	Inactive	CHEMBL506814	2.613	Inactive
CHEMBL3640283	2.371	Inactive	CHEMBL565250	4.340	Inactive	CHEMBL1760732	2.881	Inactive
CHEMBL3640229	2.079	Inactive	CHEMBL582828	2.000	Inactive	CHEMBL1760734	3.041	Inactive
CHEMBL50	3.748	Inactive	CHEMBL565914	1.903	Active	CHEMBL435747	5.491	Inactive
CHEMBL3422235	1.431	Active	CHEMBL590492	1.845	Active	CHEMBL2177307	2.699	Inactive
CHEMBL2380458	3.898	Inactive	CHEMBL2181882	1.461	Active	CHEMBL2180030	1.079	Active
CHEMBL2380454	3.898	Inactive	CHEMBL2181880	1.225	Active	CHEMBL2180027	1.987	Active
CHEMBL2380448	3.301	Inactive	CHEMBL208809	1.380	Active	CHEMBL2322538	4.430	Inactive
CHEMBL2380809	2.851	Inactive	CHEMBL211937	3.771	Inactive	CHEMBL217374	1.954	Active
CHEMBL2380806	3.462	Inactive	CHEMBL129482	4.447	Inactive	CHEMBL566198	2.519	Inactive
CHEMBL566638	1.845	Active	CHEMBL429477	1.820	Active	CHEMBL2407489	0.771	Active
CHEMBL248922	2.064	Inactive	CHEMBL207083	3.000	Inactive	CHEMBL206651	3.230	Inactive
CHEMBL401338	3.700	Inactive	CHEMBL114169	3.114	Inactive	CHEMBL2314780	3.623	Inactive
CHEMBL252960	3.578	Inactive	CHEMBL271709	2.083	Inactive	CHEMBL2407490	0.398	Active
CHEMBL594896	3.134	Inactive	CHEMBL407904	2.124	Inactive	CHEMBL2407339	0.672	Active
CHEMBL584509	2.568	Inactive	CHEMBL443239	2.699	Inactive	CHEMBL202549	3.146	Inactive
CHEMBL239046	1.996	Active	CHEMBL252756	3.810	Inactive	CHEMBL2407488	0.398	Active
CHEMBL473159	4.562	Inactive	CHEMBL252755	3.749	Inactive	CHEMBL2407340	1.908	Active
CHEMBL1957469	1.350	Active	CHEMBL3422236	0.602	Active	CHEMBL3597555	3.775	Inactive
CHEMBL589163	3.033	Inactive	CHEMBL364446	2.279	Inactive	CHEMBL2347210	1.415	Active
CHEMBL471187	4.086	Inactive	CHEMBL2407345	1.556	Active	CHEMBL3937141	4.914	Inactive
CHEMBL1952317	3.584	Inactive	CHEMBL2347190	1.690	Active	CHEMBL2058872	3.868	Inactive
CHEMBL1952316	3.449	Inactive	CHEMBL2347196	1.398	Active	CHEMBL565790	2.079	Inactive

Table S2. (*Cont.*)

Molecule ChEMBL ID	$logIC_{50}$ (nM)	Label	Molecule ChEMBL ID	$logIC_{50}$ (nM)	Label	Molecule ChEMBL ID	$logIC_{50}$ (nM)	Label
CHEMBL566968	2.279	Inactive	CHEMBL3394051	-0.301	Active	CHEMBL434943	1.204	Active
CHEMBL1821810	3.716	Inactive	CHEMBL1760861	3.505	Inactive	CHEMBL568115	1.301	Active
CHEMBL1821820	-0.187	Active	CHEMBL1760865	1.740	Active	CHEMBL1271244	1.000	Active
CHEMBL597062	2.146	Inactive	CHEMBL3354713	0.000	Active	CHEMBL1269918	3.093	Inactive
CHEMBL1615184	2.176	Inactive	CHEMBL370043	1.762	Active	CHEMBL130415	3.380	Inactive
CHEMBL405889	3.111	Inactive	CHEMBL3236220	3.856	Inactive	CHEMBL3354697	0.845	Active
CHEMBL265953	3.998	Inactive	CHEMBL2177485	4.320	Inactive	CHEMBL3354706	0.477	Active
CHEMBL405204	4.562	Inactive	CHEMBL2181915	0.799	Active	CHEMBL3354711	0.845	Active
CHEMBL212627	1.898	Active	CHEMBL2181905	0.568	Active	CHEMBL3260842	1.301	Active
CHEMBL594656	3.895	Inactive	CHEMBL567259	2.716	Inactive	CHEMBL1086716	3.669	Inactive
CHEMBL2181982	3.797	Inactive	CHEMBL2181978	4.923	Inactive	CHEMBL2346801	2.748	Inactive
CHEMBL2177472	3.230	Inactive	CHEMBL2177470	2.000	Inactive	CHEMBL2177322	3.223	Inactive
CHEMBL2177320	3.072	Inactive	CHEMBL2425599	1.000	Active	CHEMBL3645161	1.903	Active
CHEMBL2177329	3.068	Inactive	CHEMBL1957472	2.030	Inactive	CHEMBL3640219	1.903	Active
CHEMBL2177339	2.613	Inactive	CHEMBL1957482	1.770	Active	CHEMBL2151151	0.778	Active
CHEMBL2177343	0.903	Active	CHEMBL3673194	1.886	Active	CHEMBL2177915	0.812	Active
CHEMBL2177305	0.396	Active	CHEMBL4085715	4.314	Inactive	CHEMBL2180036	2.903	Inactive
CHEMBL1271140	1.778	Active	CHEMBL4066885	3.770	Inactive	CHEMBL2180026	2.519	Inactive
CHEMBL1270134	1.778	Active	CHEMBL4091480	4.866	Inactive	CHEMBL3645167	2.346	Inactive
CHEMBL1270337	1.602	Active	CHEMBL253865	2.556	Inactive	CHEMBL3645174	1.732	Active
CHEMBL1270832	1.602	Active	CHEMBL566007	3.352	Inactive	CHEMBL3640260	1.322	Active
CHEMBL1271036	1.477	Active	CHEMBL4086961	0.021	Active	CHEMBL3640285	1.602	Active
CHEMBL2177341	4.017	Inactive	CHEMBL453642	3.924	Inactive	CHEMBL125743	3.398	Inactive
CHEMBL2177301	1.111	Active	CHEMBL3414711	2.176	Inactive	CHEMBL3640247	2.204	Inactive
CHEMBL2177314	3.914	Inactive	CHEMBL3414703	4.322	Inactive	CHEMBL1821814	0.568	Active
CHEMBL2425618	1.255	Active	CHEMBL3086238	4.107	Inactive	CHEMBL386666	3.462	Inactive
CHEMBL2181998	5.057	Inactive	CHEMBL1762684	3.959	Inactive	CHEMBL191100	3.525	Inactive
CHEMBL2177482	3.785	Inactive	CHEMBL2177323	3.250	Inactive	CHEMBL295996	3.447	Inactive
CHEMBL2407495	2.041	Inactive	CHEMBL3287407	4.794	Inactive	CHEMBL2048062	1.556	Active
CHEMBL3604596	4.748	Inactive	CHEMBL3287409	3.627	Inactive	CHEMBL3642632	2.960	Inactive
CHEMBL2347208	1.079	Active	CHEMBL567053	3.903	Inactive	CHEMBL2314781	4.633	Inactive
CHEMBL2181908	0.568	Active	CHEMBL56002	4.301	Inactive	CHEMBL3693258	1.908	Active
CHEMBL2181906	0.279	Active	CHEMBL593979	3.143	Inactive	CHEMBL1760730	4.740	Inactive
CHEMBL3642601	3.462	Inactive	CHEMBL2177920	1.190	Active	CHEMBL2380439	5.000	Inactive
CHEMBL2346797	3.528	Inactive	CHEMBL2177919	1.784	Active	CHEMBL2380445	3.000	Inactive
CHEMBL3642633	3.176	Inactive	CHEMBL2177910	3.140	Inactive	CHEMBL2380455	2.898	Inactive
CHEMBL3951035	3.170	Inactive	CHEMBL2177909	2.097	Inactive	CHEMBL2346789	3.733	Inactive
CHEMBL3645165	2.386	Inactive	CHEMBL568506	3.851	Inactive	CHEMBL2425611	0.903	Active
CHEMBL2425608	0.301	Active	CHEMBL584267	1.845	Active	CHEMBL3640258	1.785	Active
CHEMBL3640251	2.509	Inactive	CHEMBL567477	1.000	Active	CHEMBL3640257	0.875	Active
CHEMBL3640256	1.820	Active	CHEMBL243148	3.716	Inactive	CHEMBL3640277	1.491	Active
CHEMBL3640282	1.799	Active	CHEMBL492828	4.442	Inactive	CHEMBL3659644	1.799	Active
CHEMBL3640263	1.204	Active	CHEMBL243796	3.519	Inactive	CHEMBL4173236	2.111	Inactive
CHEMBL3645158	2.255	Inactive	CHEMBL377149	2.193	Inactive	CHEMBL260621	2.176	Inactive
CHEMBL3640226	2.204	Inactive	CHEMBL1601822	2.422	Inactive	CHEMBL4203860	2.431	Inactive
CHEMBL4090604	3.176	Inactive	CHEMBL1312649	3.110	Inactive	CHEMBL4215837	0.968	Active
CHEMBL3265332	2.556	Inactive	CHEMBL3642635	3.661	Inactive	CHEMBL2180014	1.785	Active
CHEMBL211995	2.477	Inactive	CHEMBL3645170	2.204	Inactive	CHEMBL2180024	2.690	Inactive
CHEMBL3639440	2.176	Inactive	CHEMBL3645149	2.204	Inactive	CHEMBL3236217	3.342	Inactive
CHEMBL3394048	-0.155	Active	CHEMBL1821830	1.176	Active	CHEMBL3604697	3.973	Inactive

Table S2. (*Cont.*)

Molecule ChEMBL ID	$logIC_{50}$ (nM)	Label	Molecule ChEMBL ID	$logIC_{50}$ (nM)	Label	Molecule ChEMBL ID	$logIC_{50}$ (nM)	Label
CHEMBL3659643	3.505	Inactive	CHEMBL2346799	3.859	Inactive	CHEMBL4206426	4.501	Inactive
CHEMBL1760855	2.996	Inactive	CHEMBL2425607	2.354	Inactive	CHEMBL1762560	3.814	Inactive
CHEMBL1589705	2.217	Inactive	CHEMBL2425610	1.519	Active	CHEMBL1762554	4.099	Inactive
CHEMBL3645162	4.000	Inactive	CHEMBL445281	1.176	Active	CHEMBL3086245	4.258	Inactive
CHEMBL3645169	2.703	Inactive	CHEMBL1760867	3.623	Inactive	CHEMBL4208507	3.591	Inactive
CHEMBL3645175	2.921	Inactive	CHEMBL2047900	2.301	Inactive	CHEMBL4206849	3.477	Inactive
CHEMBL2181909	1.041	Active	CHEMBL2048060	0.778	Active	CHEMBL4289749	1.491	Active
CHEMBL2181904	1.041	Active	CHEMBL501242	1.114	Active	CHEMBL385374	0.602	Active
CHEMBL2181903	0.978	Active	CHEMBL2172795	4.009	Inactive	CHEMBL2047901	2.146	Inactive
CHEMBL2181902	0.279	Active	CHEMBL1934197	4.045	Inactive	CHEMBL2047904	2.940	Inactive
CHEMBL2181899	1.672	Active	CHEMBL2314788	4.037	Inactive	CHEMBL2048044	0.778	Active
CHEMBL2181889	0.968	Active	CHEMBL2403775	2.079	Inactive	CHEMBL3359756	0.519	Active
CHEMBL2177334	3.507	Inactive	CHEMBL2403766	1.079	Active	CHEMBL3359749	-0.398	Active
CHEMBL2322537	4.525	Inactive	CHEMBL3746566	2.322	Inactive	CHEMBL1270779	4.301	Inactive
CHEMBL2425601	1.079	Active	CHEMBL3746637	1.699	Active	CHEMBL1271399	4.301	Inactive
CHEMBL2425614	2.724	Inactive	CHEMBL939	4.301	Inactive	CHEMBL1271141	1.000	Active
CHEMBL2380816	2.785	Inactive	CHEMBL2181022	1.734	Active	CHEMBL1270241	2.301	Inactive
CHEMBL2380450	2.799	Inactive	CHEMBL3400758	3.649	Inactive	CHEMBL3394039	-0.155	Active
CHEMBL2177474	3.230	Inactive	CHEMBL3359747	-0.155	Active	CHEMBL3394047	0.602	Active
CHEMBL2177471	4.041	Inactive	CHEMBL2331708	1.431	Active	CHEMBL3354715	-0.398	Active
CHEMBL2180020	4.255	Inactive	CHEMBL2177319	3.723	Inactive	CHEMBL2177911	2.603	Inactive
CHEMBL2181916	0.964	Active	CHEMBL3763944	2.987	Inactive	CHEMBL2177113	1.937	Active
CHEMBL231160	2.033	Inactive	CHEMBL2047915	1.653	Active	CHEMBL2177326	3.305	Inactive
CHEMBL261063	3.643	Inactive	CHEMBL2048037	1.556	Active	CHEMBL2177316	3.083	Inactive
CHEMBL2407491	0.987	Active	CHEMBL2048039	1.380	Active	CHEMBL2181896	0.531	Active
CHEMBL3640266	0.978	Active	CHEMBL2047897	3.439	Inactive	CHEMBL2181891	1.806	Active
CHEMBL2403769	1.342	Active	CHEMBL2059104	3.945	Inactive	CHEMBL3936271	1.785	Active
CHEMBL2347198	0.778	Active	CHEMBL1760731	3.041	Inactive	CHEMBL2177489	4.364	Inactive
CHEMBL2347212	1.146	Active	CHEMBL1760857	2.279	Inactive	CHEMBL2177488	3.114	Inactive
CHEMBL3640286	2.350	Inactive	CHEMBL1760858	2.568	Inactive	CHEMBL2177487	3.978	Inactive
CHEMBL3645152	2.176	Inactive	CHEMBL3394049	-0.222	Active	CHEMBL2180032	3.415	Inactive
CHEMBL4128807	4.281	Inactive	CHEMBL2347202	1.279	Active	CHEMBL2180023	2.230	Inactive
CHEMBL2347215	1.362	Active	CHEMBL2347194	1.826	Active	CHEMBL2177311	3.314	Inactive
CHEMBL3659647	3.699	Inactive	CHEMBL2181828	1.685	Active	CHEMBL2177318	2.981	Inactive
CHEMBL2380818	3.386	Inactive	CHEMBL2181892	0.613	Active	CHEMBL1760856	2.176	Inactive
CHEMBL576628	3.540	Inactive	CHEMBL2177484	2.431	Inactive	CHEMBL3634342	0.857	Active
CHEMBL3634122	1.643	Active	CHEMBL2177478	3.176	Inactive	CHEMBL4174699	1.480	Active
CHEMBL3765061	3.430	Inactive	CHEMBL2177475	3.362	Inactive	CHEMBL3586207	3.839	Inactive
CHEMBL3359757	-0.046	Active	CHEMBL2181887	0.903	Active	CHEMBL2347193	1.415	Active
CHEMBL3236218	3.207	Inactive	CHEMBL2180021	3.322	Inactive	CHEMBL2347366	1.756	Active
CHEMBL3414706	3.708	Inactive	CHEMBL2403760	3.953	Inactive	CHEMBL2347192	1.230	Active
CHEMBL2048038	1.748	Active	CHEMBL2403764	3.602	Inactive	CHEMBL4288556	2.505	Inactive
CHEMBL2048042	2.079	Inactive	CHEMBL2403778	1.663	Active	CHEMBL2347213	2.468	Inactive
CHEMBL2048047	0.301	Active	CHEMBL2403777	1.362	Active	CHEMBL2403771	2.881	Inactive
CHEMBL2048048	2.000	Inactive	CHEMBL2403776	1.146	Active	CHEMBL2333941	2.378	Inactive
CHEMBL2047889	1.740	Active	CHEMBL3265333	2.477	Inactive	CHEMBL3919597	1.519	Active
CHEMBL2047899	2.079	Inactive	CHEMBL3265338	4.230	Inactive	CHEMBL2059605	4.134	Inactive
CHEMBL2047905	0.903	Active	CHEMBL2180015	2.114	Inactive	CHEMBL3414702	4.556	Inactive
CHEMBL2180028	1.505	Active	CHEMBL2180022	3.114	Inactive	CHEMBL2407344	1.230	Active
CHEMBL2180019	4.114	Inactive	CHEMBL4211913	1.338	Active	CHEMBL2425606	1.279	Active

Table S2. (*Cont.*)

Molecule ChEMBL ID	logIC$_{50}$ (nM)	Label	Molecule ChEMBL ID	logIC$_{50}$ (nM)	Label	Molecule ChEMBL ID	logIC$_{50}$ (nM)	Label
CHEMBL2425613	0.845	Active	CHEMBL2177477	3.491	Inactive	CHEMBL4204042	3.580	Inactive
CHEMBL3642641	3.358	Inactive	CHEMBL396338	1.869	Active	CHEMBL2380810	3.267	Inactive
CHEMBL3640249	1.845	Active	CHEMBL2047893	2.114	Inactive	CHEMBL3261072	1.310	Active
CHEMBL3640291	1.623	Active	CHEMBL2403763	4.033	Inactive	CHEMBL4127426	1.079	Active
CHEMBL3640292	2.037	Inactive	CHEMBL2403779	2.568	Inactive	CHEMBL3642636	3.561	Inactive
CHEMBL3265335	3.000	Inactive	CHEMBL2172801	4.173	Inactive	CHEMBL3400753	2.940	Inactive
CHEMBL2347206	1.732	Active	CHEMBL2172793	4.100	Inactive	CHEMBL3354707	0.602	Active
CHEMBL2347364	1.505	Active	CHEMBL2047907	1.973	Active	CHEMBL4126751	4.417	Inactive
CHEMBL413999	3.294	Inactive	CHEMBL2047909	1.898	Active	CHEMBL2059453	4.330	Inactive
CHEMBL2059592	4.041	Inactive	CHEMBL3414709	2.672	Inactive	CHEMBL3394042	0.851	Active
CHEMBL2059597	4.558	Inactive	CHEMBL510508	3.328	Inactive	CHEMBL3394222	0.000	Active
CHEMBL2058868	3.732	Inactive	CHEMBL3359754	0.748	Active	CHEMBL3394225	-0.523	Active
CHEMBL3597548	4.127	Inactive	CHEMBL3359750	2.394	Inactive	CHEMBL2380813	2.653	Inactive
CHEMBL448015	1.342	Active	CHEMBL3394040	-0.046	Active	CHEMBL3752684	4.255	Inactive
CHEMBL1955882	2.420	Inactive	CHEMBL3752926	2.633	Inactive	CHEMBL2181900	1.851	Active
CHEMBL1957476	1.480	Active	CHEMBL567341	4.800	Inactive	CHEMBL2181888	1.519	Active
CHEMBL1760854	3.079	Inactive	CHEMBL1271343	2.230	Inactive	CHEMBL2177338	3.591	Inactive
CHEMBL2172797	4.732	Inactive	CHEMBL1271450	1.477	Active	CHEMBL3642614	2.653	Inactive
CHEMBL2177302	0.917	Active	CHEMBL1270528	2.732	Inactive	CHEMBL3359762	0.079	Active
CHEMBL2425603	1.903	Active	CHEMBL1270629	1.301	Active	CHEMBL3359751	0.255	Active
CHEMBL2425612	0.602	Active	CHEMBL1270135	2.000	Inactive	CHEMBL2177469	3.079	Inactive
CHEMBL2425605	1.322	Active	CHEMBL1270136	2.301	Inactive	CHEMBL2177480	3.230	Inactive
CHEMBL1614769	2.978	Inactive	CHEMBL1270435	1.000	Active	CHEMBL2172803	4.386	Inactive
CHEMBL2177324	3.688	Inactive	CHEMBL1270530	1.845	Active	CHEMBL2169947	4.727	Inactive
CHEMBL3394054	0.398	Active	CHEMBL1760729	3.255	Inactive	CHEMBL4205912	1.623	Active
CHEMBL2347189	1.602	Active	CHEMBL1762688	4.047	Inactive	CHEMBL3586198	2.991	Inactive
CHEMBL2347186	1.886	Active	CHEMBL3747444	2.826	Inactive	CHEMBL3086249	3.806	Inactive
CHEMBL2347200	2.179	Inactive	CHEMBL583899	2.672	Inactive	CHEMBL1952315	3.356	Inactive
CHEMBL2346796	2.875	Inactive	CHEMBL2365642	4.615	Inactive	CHEMBL2347363	1.940	Active
CHEMBL2177308	2.127	Inactive	CHEMBL3394058	0.415	Active	CHEMBL2181917	0.505	Active
CHEMBL4128947	4.573	Inactive	CHEMBL2019055	1.519	Active	CHEMBL4169060	1.455	Active
CHEMBL2177107	0.699	Active	CHEMBL3394212	-0.398	Active	CHEMBL496451	3.826	Inactive
CHEMBL2181894	0.839	Active	CHEMBL3394227	-0.699	Active	CHEMBL590830	1.602	Active
CHEMBL2177337	3.324	Inactive	CHEMBL3653409	1.964	Active	CHEMBL4161795	1.029	Active
CHEMBL2030997	0.699	Active	CHEMBL4129637	2.316	Inactive	CHEMBL2058874	3.634	Inactive
CHEMBL2407487	0.785	Active	CHEMBL4127062	3.663	Inactive	CHEMBL259083	4.324	Inactive
CHEMBL3326709	4.354	Inactive	CHEMBL3354689	0.301	Active	CHEMBL260635	4.066	Inactive
CHEMBL2380449	3.301	Inactive	CHEMBL3354695	1.041	Active	CHEMBL591070	2.934	Inactive
CHEMBL2181895	0.756	Active	CHEMBL3354699	0.903	Active	CHEMBL3640222	2.114	Inactive
CHEMBL2177468	3.204	Inactive	CHEMBL3354709	-0.097	Active	CHEMBL3640237	2.041	Inactive
CHEMBL2177476	3.602	Inactive	CHEMBL1222240	3.845	Inactive	CHEMBL412768	2.301	Inactive
CHEMBL2181827	1.417	Active	CHEMBL2169923	4.845	Inactive	CHEMBL3747556	2.591	Inactive
CHEMBL2181883	1.884	Active	CHEMBL2169943	4.667	Inactive	CHEMBL1957473	2.000	Inactive
CHEMBL208903	1.663	Active	CHEMBL2181981	4.470	Inactive	CHEMBL4084653	3.621	Inactive
CHEMBL379067	2.000	Inactive	CHEMBL2181912	0.857	Active	CHEMBL4061944	4.143	Inactive
CHEMBL378032	3.000	Inactive	CHEMBL2181830	0.732	Active	CHEMBL4100140	4.707	Inactive
CHEMBL2322536	4.933	Inactive	CHEMBL3586196	3.029	Inactive	CHEMBL3326698	4.603	Inactive
CHEMBL2059606	4.405	Inactive	CHEMBL2059611	4.013	Inactive	CHEMBL399839	2.146	Inactive
CHEMBL2407338	1.964	Active	CHEMBL3359752	0.176	Active	CHEMBL400043	1.699	Active
CHEMBL2047894	3.210	Inactive	CHEMBL3897492	2.934	Inactive	CHEMBL3586197	2.362	Inactive

Table S2. (*Cont.*)

Molecule ChEMBL ID	logIC50 (nM)	Label	Molecule ChEMBL ID	logIC50 (nM)	Label	Molecule ChEMBL ID	logIC50 (nM)	Label
CHEMBL2177906	2.382	Inactive	CHEMBL2181015	1.903	Active	CHEMBL3359759	0.362	Active
CHEMBL2047910	2.176	Inactive	CHEMBL2443370	3.831	Inactive	CHEMBL3883958	4.223	Inactive
CHEMBL2047913	1.892	Active	CHEMBL2443373	3.624	Inactive	CHEMBL3400767	2.681	Inactive
CHEMBL1271344	1.477	Active	CHEMBL2322539	4.806	Inactive	CHEMBL3640245	1.602	Active
CHEMBL1270727	1.477	Active	CHEMBL3645157	2.041	Inactive	CHEMBL4212046	2.041	Inactive
CHEMBL1270730	1.699	Active	CHEMBL2380812	3.100	Inactive	CHEMBL4217023	2.996	Inactive
CHEMBL3394057	-0.046	Active	CHEMBL2380807	2.491	Inactive	CHEMBL4204215	3.283	Inactive
CHEMBL3394221	-0.523	Active	CHEMBL131770	3.405	Inactive	CHEMBL4096411	4.481	Inactive
CHEMBL3394052	0.146	Active	CHEMBL3747119	2.778	Inactive	CHEMBL4175845	1.265	Active
CHEMBL252189	2.778	Inactive	CHEMBL2407343	2.079	Inactive	CHEMBL3394224	-0.523	Active
CHEMBL2347197	1.114	Active	CHEMBL498456	3.679	Inactive	CHEMBL3394053	0.580	Active
CHEMBL2346800	2.230	Inactive	CHEMBL3354703	2.509	Inactive	CHEMBL4098403	1.839	Active
CHEMBL3086246	3.672	Inactive	CHEMBL3354704	0.602	Active	CHEMBL4086633	3.117	Inactive
CHEMBL3086242	4.170	Inactive	CHEMBL129795	4.712	Inactive	CHEMBL4159379	3.230	Inactive
CHEMBL3301601	0.342	Active	CHEMBL3640238	2.230	Inactive	CHEMBL4204163	1.860	Active
CHEMBL3645155	1.954	Active	CHEMBL3640244	1.699	Active	CHEMBL3604598	4.531	Inactive
CHEMBL3642613	2.912	Inactive	CHEMBL3400765	3.004	Inactive	CHEMBL4207711	3.124	Inactive
CHEMBL3659639	2.954	Inactive	CHEMBL3642617	3.447	Inactive	CHEMBL4212215	3.813	Inactive
CHEMBL2314792	3.380	Inactive	CHEMBL3642611	2.193	Inactive	CHEMBL3909581	1.519	Active
CHEMBL2314789	4.097	Inactive	CHEMBL3640264	1.505	Active	CHEMBL2403762	2.740	Inactive
CHEMBL1760860	1.778	Active	CHEMBL3640275	1.968	Active	CHEMBL2380443	4.000	Inactive
CHEMBL2047906	1.716	Active	CHEMBL3642630	3.296	Inactive	CHEMBL3422242	0.778	Active
CHEMBL2047895	3.161	Inactive	CHEMBL3645163	2.217	Inactive	CHEMBL3640271	2.543	Inactive
CHEMBL2047903	1.785	Active	CHEMBL1526260	3.176	Inactive	CHEMBL1914472	0.301	Active
CHEMBL2047911	2.114	Inactive	CHEMBL3640223	2.079	Inactive	CHEMBL4208381	3.279	Inactive
CHEMBL3642642	2.114	Inactive	CHEMBL2380461	4.204	Inactive	CHEMBL2047896	2.342	Inactive
CHEMBL3659640	4.041	Inactive	CHEMBL3422243	0.477	Active	CHEMBL2047902	2.748	Inactive
CHEMBL3640287	1.826	Active	CHEMBL3422244	1.000	Active	CHEMBL3642616	3.903	Inactive
CHEMBL4127289	4.386	Inactive	CHEMBL3642604	2.444	Inactive	CHEMBL3642615	3.653	Inactive
CHEMBL412768	2.301	Inactive	CHEMBL3642609	3.898	Inactive	CHEMBL3950821	3.771	Inactive
CHEMBL3890737	2.708	Inactive	CHEMBL3642612	3.826	Inactive	CHEMBL3907072	3.398	Inactive
CHEMBL257091	2.785	Inactive	CHEMBL4064412	4.083	Inactive	CHEMBL3642600	1.595	Active
CHEMBL4129873	4.176	Inactive	CHEMBL3634123	1.643	Active	CHEMBL3745864	2.756	Inactive
CHEMBL4128586	3.886	Inactive	CHEMBL2380441	4.505	Inactive	CHEMBL4128406	1.398	Active
CHEMBL222274	3.748	Inactive	CHEMBL3746636	2.556	Inactive	CHEMBL4126197	1.724	Active
CHEMBL3326704	4.236	Inactive	CHEMBL3747274	3.130	Inactive	CHEMBL2169938	4.595	Inactive
CHEMBL4294221	-0.208	Active	CHEMBL1483796	3.820	Inactive	CHEMBL3980733	1.568	Active
CHEMBL3394219	-0.398	Active	CHEMBL3746896	2.681	Inactive	CHEMBL3236225	3.276	Inactive
CHEMBL3327247	4.631	Inactive	CHEMBL4208775	3.462	Inactive	CHEMBL2346803	4.867	Inactive
CHEMBL2380815	2.799	Inactive	CHEMBL4203347	3.713	Inactive	CHEMBL2177918	1.620	Active
CHEMBL2347367	2.265	Inactive	CHEMBL1610040	2.968	Inactive	CHEMBL2177309	2.199	Inactive
CHEMBL4066788	3.835	Inactive	CHEMBL3642631	3.510	Inactive	CHEMBL2059596	3.729	Inactive
CHEMBL4127412	4.127	Inactive	CHEMBL3642640	2.342	Inactive	CHEMBL3354691	0.301	Active
CHEMBL4127986	4.587	Inactive	CHEMBL3645168	1.568	Active	CHEMBL3354712	0.602	Active
CHEMBL201049	3.114	Inactive	CHEMBL3645176	3.361	Inactive	CHEMBL3642602	3.845	Inactive
CHEMBL382233	3.176	Inactive	CHEMBL3645177	2.241	Inactive	CHEMBL3642622	3.602	Inactive
CHEMBL3394043	1.053	Active	CHEMBL4088234	1.892	Active	CHEMBL3642599	3.886	Inactive
CHEMBL370801	2.863	Inactive	CHEMBL4080380	2.886	Inactive	CHEMBL2346790	3.453	Inactive
CHEMBL3394217	-0.398	Active	CHEMBL4104527	4.305	Inactive	CHEMBL2347204	1.447	Active
CHEMBL3394220	-0.523	Active	CHEMBL4092406	3.914	Inactive	CHEMBL2347205	2.638	Inactive

Table S2. (*Cont.*)

Molecule ChEMBL ID	logIC$_{50}$ (nM)	Label	Molecule ChEMBL ID	logIC$_{50}$ (nM)	Label	Molecule ChEMBL ID	logIC$_{50}$ (nM)	Label
CHEMBL2347201	1.556	Active	CHEMBL3653445	1.146	Active	CHEMBL4165225	1.346	Active
CHEMBL2347365	1.708	Active	CHEMBL3653413	3.049	Inactive	CHEMBL3672916	-0.046	Active
CHEMBL3659645	3.519	Inactive	CHEMBL3586204	2.491	Inactive	CHEMBL4211220	3.398	Inactive
CHEMBL3640273	1.789	Active	CHEMBL3763318	2.146	Inactive	CHEMBL4204582	3.431	Inactive
CHEMBL3640278	1.415	Active	CHEMBL3640289	1.322	Active	CHEMBL4211801	3.230	Inactive
CHEMBL3640265	2.068	Inactive	CHEMBL3640268	1.255	Active	CHEMBL4202435	3.556	Inactive
CHEMBL3640262	1.217	Active	CHEMBL3645160	1.954	Active	CHEMBL3818084	2.522	Inactive
CHEMBL4127550	4.471	Inactive	CHEMBL3645171	1.431	Active	CHEMBL4206331	3.580	Inactive
CHEMBL4130015	4.441	Inactive	CHEMBL3645172	1.505	Active	CHEMBL4204309	3.447	Inactive
CHEMBL4209354	3.782	Inactive	CHEMBL3265330	2.041	Inactive	CHEMBL1492021	3.477	Inactive
CHEMBL2059329	4.021	Inactive	CHEMBL2396987	4.342	Inactive	CHEMBL3884069	4.317	Inactive
CHEMBL4287410	0.875	Active	CHEMBL3261046	1.301	Active	CHEMBL4083698	4.190	Inactive
CHEMBL2059594	4.182	Inactive	CHEMBL3261050	2.711	Inactive	CHEMBL3653410	1.580	Active
CHEMBL3910646	2.204	Inactive	CHEMBL4277255	1.857	Active	CHEMBL4218591	3.613	Inactive
CHEMBL2407496	1.653	Active	CHEMBL4279064	0.556	Active	CHEMBL4218068	3.431	Inactive
CHEMBL4279496	0.740	Active	CHEMBL3261067	0.699	Active	CHEMBL4207174	3.415	Inactive
CHEMBL3422245	1.176	Active	CHEMBL3422241	0.778	Active	CHEMBL168938	2.843	Inactive
CHEMBL3642620	3.756	Inactive	CHEMBL3261079	0.924	Active	CHEMBL3640230	3.966	Inactive
CHEMBL3642618	3.409	Inactive	CHEMBL2347211	0.826	Active	CHEMBL4217620	-0.222	Active
CHEMBL3645151	2.146	Inactive	CHEMBL3917530	1.780	Active	CHEMBL4128770	1.301	Active
CHEMBL4159574	1.610	Active	CHEMBL4061602	3.294	Inactive	CHEMBL4202984	3.380	Inactive
CHEMBL3640284	1.491	Active	CHEMBL4102593	3.322	Inactive	CHEMBL3604597	4.806	Inactive
CHEMBL4094377	2.690	Inactive	CHEMBL4217236	3.505	Inactive	CHEMBL4175758	3.370	Inactive
CHEMBL4126598	4.243	Inactive	CHEMBL3653341	1.845	Active	CHEMBL4291981	1.322	Active
CHEMBL3640233	3.832	Inactive	CHEMBL4284110	-0.509	Active	CHEMBL4170106	2.180	Active
CHEMBL3640236	1.000	Active	CHEMBL4282964	0.255	Active	CHEMBL4162197	1.057	Active
CHEMBL4174503	3.756	Inactive	CHEMBL3659641	4.431	Inactive	CHEMBL4167487	1.389	Active
CHEMBL3891534	1.301	Active	CHEMBL3967984	1.464	Active	CHEMBL4171250	3.480	Inactive
CHEMBL3986498	2.362	Inactive	CHEMBL4062641	3.255	Inactive	CHEMBL4160644	2.660	Inactive
CHEMBL3928503	3.301	Inactive	CHEMBL3640255	1.544	Active	CHEMBL4167898	3.270	Inactive
CHEMBL3645164	2.212	Inactive	CHEMBL3640254	2.822	Inactive	CHEMBL4211219	3.521	Inactive
CHEMBL3645166	1.690	Active	CHEMBL3941301	2.729	Inactive	CHEMBL4216181	3.767	Inactive
CHEMBL3972449	1.358	Active	CHEMBL3952064	1.567	Active	CHEMBL4204085	3.851	Inactive
CHEMBL3640290	1.146	Active	CHEMBL4276834	-0.337	Active	CHEMBL4280656	1.114	Active
CHEMBL3640267	1.699	Active	CHEMBL3693257	2.568	Inactive	CHEMBL4285211	-0.036	Active
CHEMBL3634125	1.079	Active	CHEMBL4205504	3.204	Inactive	CHEMBL4290822	-0.237	Active
CHEMBL4165629	1.470	Active	CHEMBL3658614	2.380	Inactive	CHEMBL4286331	-0.244	Active
CHEMBL3642603	3.699	Inactive	CHEMBL4176954	3.330	Inactive	CHEMBL4128727	4.176	Inactive
CHEMBL3642621	3.908	Inactive	CHEMBL3947194	2.029	Inactive	CHEMBL3653512	1.431	Active
CHEMBL3900541	2.778	Inactive	CHEMBL4128145	3.032	Inactive	CHEMBL3745974	2.653	Inactive
CHEMBL3597554	3.396	Inactive	CHEMBL3597549	4.067	Inactive	CHEMBL4126839	1.940	Active
CHEMBL4130130	2.846	Inactive	CHEMBL4127572	1.380	Active	CHEMBL3983988	2.250	Inactive
CHEMBL4128824	1.881	Active	CHEMBL502	2.228	Inactive	CHEMBL3979124	2.360	Inactive
CHEMBL3746261	1.845	Active	CHEMBL4214603	3.962	Inactive	CHEMBL3922902	2.415	Inactive
CHEMBL3745922	2.820	Inactive	CHEMBL3642638	2.360	Inactive	CHEMBL4214785	4.378	Inactive
CHEMBL3746752	2.279	Inactive	CHEMBL4094980	3.415	Inactive	CHEMBL4062728	2.525	Inactive
CHEMBL3754357	3.808	Inactive	CHEMBL3642639	2.243	Inactive	CHEMBL4064148	4.188	Inactive
CHEMBL4204985	3.756	Inactive	CHEMBL4127672	3.623	Inactive	CHEMBL4084170	4.738	Inactive
CHEMBL4077211	2.979	Inactive	CHEMBL4128447	3.505	Inactive			
CHEMBL4064708	4.545	Inactive	CHEMBL4287483	-0.367	Active			

Table S3. Molecular descriptors of CART and CHAID models for predicting AChE inhibitory activity

Model	Code	Description	Block
**AChE-CART**	J_Dz(p)	Balaban-like index from Barysz matrix weighted by polarizability	2D matrix-based descriptors
	AVS_B(m)	average vertex sum from Burden matrix weighted by mass	2D matrix-based descriptors
	ATSC2e	Centred Broto-Moreau autocorrelation of lag 2 weighted by Sanderson electronegativity	2D autocorrelations
	C-011	CR3X	Atom-centred fragments
	H-051	H attached to alpha C	Atom-centred fragments
**AChE-CHAID**	nHM	Number of heavy atoms	Constitutional indices
	N%	Percentage of N atoms	Constitutional indices
	NNRS	Normalized number of ring systems	Ring descriptors
	Yindex	Balaban Y index	Information indices
	Eta_betaP_A	Eta pi and lone pair average VEM count	ETA indices
	SpMaxA_EA(ed)	Normalized leading eigenvalue from edge adjacency mat. weighted by edge degree	Edge adjacency indices
	SM15_EA(dm)	Spectral moment of order 15 from edge adjacency mat. weighted by dipole moment	Edge adjacency indices
	Eig02_AEA(ed)	Eigenvalue n. 2 from augmented edge adjacency mat. weighted by edge degree	Edge adjacency indices
	C-006	CH2RX	Atom-centred fragments
	H-051	H attached to alpha C	Atom-centred fragments
	O-058	Phenol / enol / carboxyl OH	Atom-centred fragments
	T(N..N)	Sum of topological distances between N...N	2D Atom Pairs
	F04[O-O]	Frequency of O - O at topological distance 4	2D Atom Pairs
	F09[C-N]	Frequency of C - N at topological distance 9	2D Atom Pairs

Table S4. Molecular descriptors of CART and CHAID models for predicting BACE1 inhibitory activity

Model	Code	Description	Block
**BACE1-CART**	GGI7	Topological charge index of order 7	2D autocorrelations
	GGI9	Topological charge index of order 9	2D autocorrelations
	P_VSA_e_3	P_VSA-like on Sanderson electronegativity, bin 3	P_VSA-like descriptors
	SM06_EA(ri)	Spectral moment of order 6 from edge adjacency mat. weighted by resonance integral	Edge adjacency indices
	C-029	R--CX--X	Atom-centred fragments
	F07[O-F]	Frequency of O - F at topological distance 7	2D autocorrelations
**BACE1-CHAID**	nCIC	number of rings (cyclomatic number)	Ring descriptors
	nR10	number of 10-membered rings	Ring descriptors
	IC1	Information Content index (neighborhood symmetry of 1-order)	Information indices
	SM06_EA(ri)	spectral moment of order 6 from edge adjacency mat. weighted by resonance integral	Edge adjacency indices
	Eig04_EA(dm)	eigenvalue n. 4 from edge adjacency mat. weighted by dipole moment	Edge adjacency indices
	nHDon	number of donor atoms for H-bonds (N and O)	Functional group counts
	C-029	R--CX--X	Atom-centred fragments

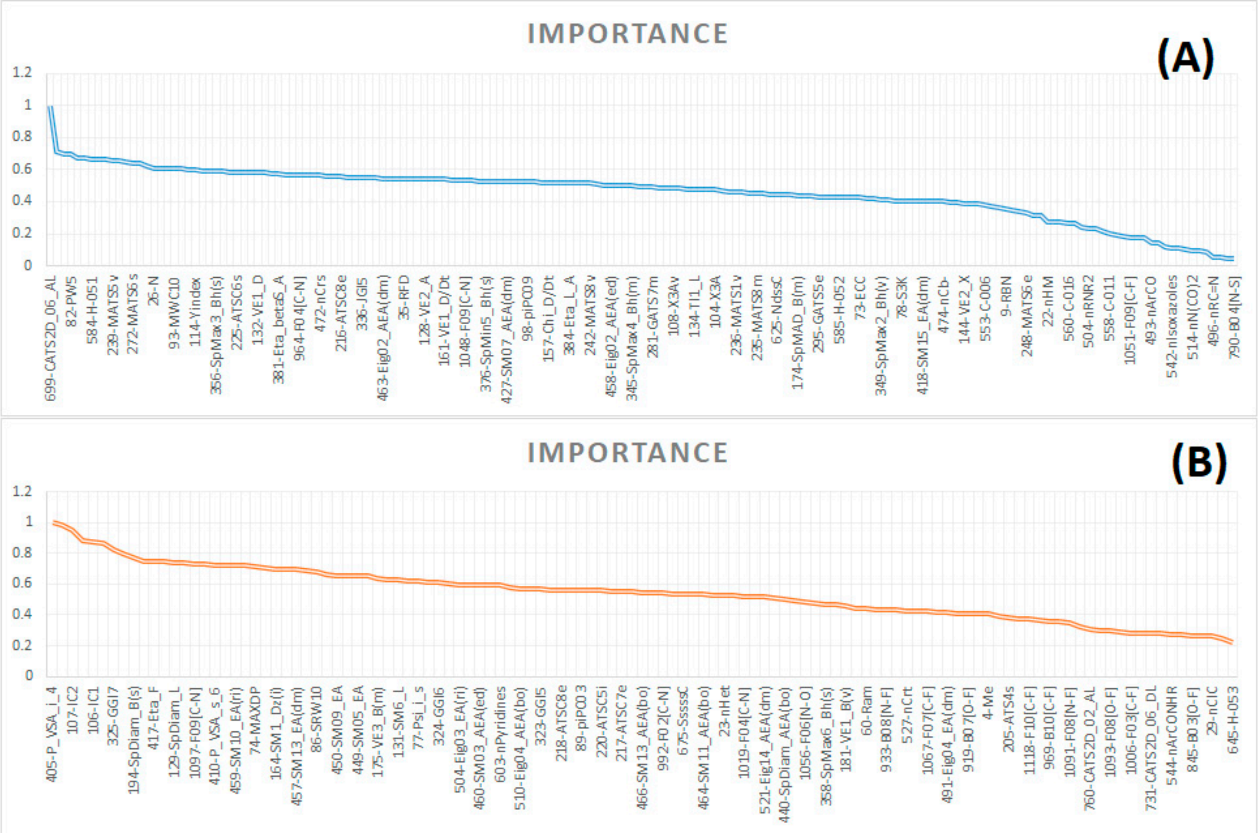

Figure S1. Variable importance according to RF models for predicting (**A**) AChE and (**B**) BACE1 inhibitory activity

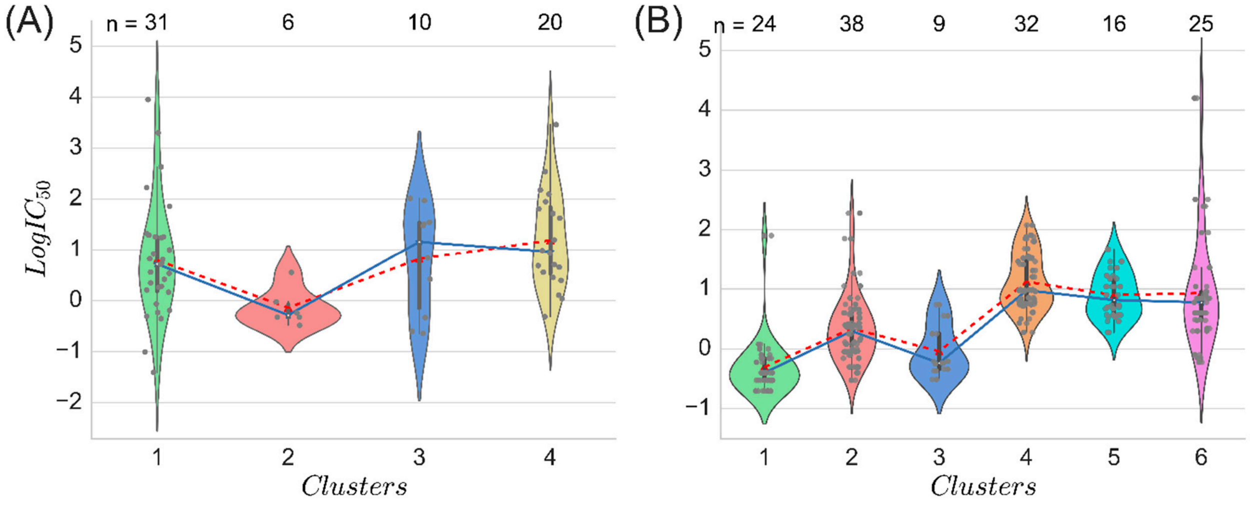

Figure S2. Distribution of experimental IC_{50} values of (**A**) 67 AChE inhibitors, (**B**) 144 BACE1 inhibitors among clusters.

Table S5. Structures and bioactivities of 67 AChE inhibitors

Molecule ChEMBL ID	SMILES	IC50 (nM)
CHEMBL372202	COc1ccc2[nH]cc(CCNC(=O)CCCCCCNc3c4c(nc5cc(Cl)cc(Cl)c35)CCCC4)c2c1	0.04
CHEMBL194823	O=C(CCCCC1CCSS1)NCCCNc1c2c(nc3cc(Cl)ccc13)CCCC2	0.253
CHEMBL219264	COc1cc(CCC(=O)NCCCCCC(=O)NNc2c3c(nc4ccccc24)CCCC3)cc(OC)c1OC	8.39
CHEMBL202661	O=C(CCCCCNc1c2c(nc3cccc(Cl)c13)CCCC2)NCCc1c[nH]c2ccccc12	0.87
CHEMBL3322142	CCN(CCC(=O)NCCCCNc1c2c(nc3ccccc13)CCCC2)C1CCCCC1	94.2
CHEMBL3355580	COc1cc(CCC(=O)/C=C/c2ccc(CNc3c4c(nc5cc(Cl)ccc35)CC3CC(C)=CC4C3)cc2)ccc1O	18.3
CHEMBL206093	Cc1ccccc1NC(=O)Oc1ccc2c(c1)[C@@]1(C)CCO[C@H]1O2	36
CHEMBL3355579	COc1cc(CCC(=O)/C=C/CCCNc2c3c(nc4cc(Cl)ccc24)CC2CC(C)=CC3C2)ccc1O	6.7
CHEMBL1651136	COc1ccccc1CN1CCC(CCC(=O)c2cc3c4c(ccn4C(=O)CC3)c2)CC1	64.57
CHEMBL1083661	O=C(CCC1CCN(Cc2ccccc2)CC1)c1ccc2c(c1)CCCN2	64.57
CHEMBL95020	O=C1Cc2c(ccc3c(CCC4CCN(Cc5ccccc5)CC4)noc23)N1	3.6
CHEMBL1912059	O=C(CCCC[C@@H]1CCSS1)NCCCNc1c2c(nc3cc(Cl)ccc13)CCCC2	0.23
CHEMBL3600552	CC1=CC2Cc3nc4cc(Cl)ccc4c(NCCCCCCCCCCCCNc4c5c(nc6cc(Cl)ccc46)CCCC5)c3C(C1)C2	3.46
CHEMBL3600556	CC1=CC2Cc3nc4ccccc4c(NCCCCCCCCCCCCNc4c5c(nc6cc(Cl)ccc46)CCCC5)c3C(C1)C2	3.66
CHEMBL219569	COc1cc(C(=O)NCCCCCCC(=O)NNc2c3c(nc4ccccc24)CCCC3)cc(OC)c1OC	5.65
CHEMBL382260	COc1ccc2[nH]cc(CCNC(=O)CCCCCCNc3c4c(nc5ccccc35)CCCC4)c2c1	0.65
CHEMBL381499	O=C(CCCCCCNc1c2c(nc3cc(Cl)ccc13)CCCC2)NCCc1c[nH]c2ccccc12	0.1
CHEMBL1651140	O=C(CCC1CCN(Cc2cccc(O)c2)CC1)c1cc2c3c(ccn3C(=O)CC2)c1	8.71
CHEMBL359570	O=C1Cc2cc3c(CCC4CCN(Cc5ccccc5)CC4)noc3cc2N1	0.331
CHEMBL32823	c1ccc2c(NCCCCCCCNc3c4c(nc5ccccc35)CCCC4)c3c(nc2c1)CCCC3	0.59
CHEMBL1651132	O=C(CCC1CCN(Cc2cccc(F)c2)CC1)c1cc2c3c(ccn3C(=O)CC2)c1	1.288
CHEMBL434378	COc1cc(C(=O)NCCCCC(=O)NNc2c3c(nc4ccccc24)CCCC3)cc(OC)c1OC	18.2
CHEMBL426441	CN1C(=O)Cc2cc3onc(CCC4CCN(Cc5ccccc5)CC4)c3cc21	0.48
CHEMBL329231	O=C1CCc2cc3c(CCC4CCN(Cc5ccccc5)CC4)noc3cc2N1	0.575
CHEMBL340625	CNC(=O)Oc1cccc(CN(C)CCCOc2ccc3ccc(=O)oc3c2)c1	5.7
CHEMBL370807	O=C(CCCCCNc1c2c(nc3ccccc13)CCCC2)NCCc1c[nH]c2ccc(O)cc12	0.45
CHEMBL1651244	O=C(CCC1CCN(Cc2ccccc2)CC1)c1cc2c3c(c1)CCC(=O)N3CCC2	15.85
CHEMBL216159	COc1cc(C(=O)NCCCCCCCC(=O)NNc2c3c(nc4ccccc24)CCCC3)cc(OC)c1OC	5.24
CHEMBL1651129	O=C(CCC1CCN(Cc2ccccc2)CC1)c1cc2c3c(c1)CC(=O)N3CCC2	3.631
CHEMBL1651250	O=C(CCC1CCN(Cc2ccc([N+](=O)[O-])cc2)CC1)c1cc2c3c(ccn3C(=O)CC2)c1	42.66
CHEMBL1651127	O=C(CCC1CCN(Cc2ccccc2)CC1)c1ccc2c(c1)CCNCC2	25.12
CHEMBL1651131	O=C(CCC1CCN(Cc2ccccc2F)CC1)c1cc2c3c(ccn3C(=O)CC2)c1	2.512
CHEMBL278963	O=C(CCCCC1CCSS1)NCCCCCCNc1c2c(nc3ccccc13)CCCC2	30.1
CHEMBL238230	O=C(CCCNc1c2c(nc3ccccc13)CCCC2)CCCNc1c2c(nc3ccccc13)CCCC2	1.83
CHEMBL195067	O=C(CCCCC1CCSS1)NCCCCCCCNc1c2c(nc3ccccc13)CCCC2	32.7
CHEMBL235014	c1ccc2c(NCCOCCOCCNc3c4c(nc5ccccc35)CCCC4)c3c(nc2c1)CCCC3	19.7
CHEMBL1651248	O=C(CCC1CCN(Cc2ccc(F)cc2)CC1)c1cc2c3c(ccn3C(=O)CC2)c1	4.571
CHEMBL328468	O=C1Cc2cc3onc(CCC4CCN(Cc5ccccc5)CC4)c3cc2N1	0.955
CHEMBL1912058	O=C(CCCC[C@H]1CCSS1)NCCCNc1c2c(nc3cc(Cl)ccc13)CCCC2	0.471
CHEMBL3355581	COc1cc(CCC(=O)/C=C/c2ccc(CNc3c4c(nc5cc(Cl)ccc35)CC3CC(C)=CC4C3)cc2)cc(CN(C)C)c1O	21.1
CHEMBL195179	O=C(CCCCC1CCSS1)NCCCNc1c2c(nc3ccccc13)CCCC2	6.96
CHEMBL1651130	O=C(CCC1CCN(Cc2ccccc2)CC1)c1cc2c3c(c1)CCN3C(=O)CC2	9.772
CHEMBL340391	CCc1ccccc1NC(=O)Oc1ccc2c(c1)[C@]1(C)CCN(C)[C@@H]1N2C	10

Table S5. (*Cont.*)

Molecule ChEMBL ID	SMILES	IC_{50} (nM)
CHEMBL92463	Oc1ccc2c(CCC3CCN(Cc4ccccc4)CC3)noc2c1	25.7
CHEMBL384886	COc1cc(CCC(=O)NCCCC(=O)NNc2c3c(nc4ccccc24)CCCC3)cc(OC)c1OC	72.1
CHEMBL199670	O=C(CCCCCCNc1c2c(nc3ccccc13)CCCC2)NCCc1c[nH]c2ccccc12	0.5
CHEMBL1651133	O=C(CCC1CCN(Cc2ccccc2Cl)CC1)c1cc2c3c(ccn3C(=O)CC2)c1	5.129
CHEMBL1651243	O=C(CCC1CCN(Cc2cccc([N+](=O)[O-])c2)CC1)c1cc2c3c(ccn3C(=O)CC2)c1	2.884
CHEMBL1651245	O=C(CCC1CCN(Cc2ccccc2)CC1)c1cc2c3c(c1)CCN3C(=O)CCC2	52.48
CHEMBL424833	O=C(CCCCC1CCSS1)NCCCCNc1c2c(nc3ccccc13)CCCC2	35.2
CHEMBL3353040	CC1=CC2Cc(n3)c(C(C2)C1)c(NCCCCCCCCNc(c4c(CC5C=C(CC4C5)C)n6)c7c6cc(Cl)cc7)c8c3cc(Cl)cc8	72.5
CHEMBL1651139	O=C(CCC1CCN(Cc2ccccc2O)CC1)c1cc2c3c(ccn3C(=O)CC2)c1	1.096
CHEMBL1651141	O=C(CCC1CCN(Cc2ccccc2[N+](=O)[O-])CC1)c1cc2c3c(ccn3C(=O)CC2)c1	89.13
CHEMBL1179697	CN1C(=O)Cc2cc3c(CCC4CCN(Cc5ccccc5)CC4)noc3cc21	0.479
CHEMBL1651134	O=C(CCC1CCN(Cc2cccc(Cl)c2)CC1)c1cc2c3c(ccn3C(=O)CC2)c1	4.898
CHEMBL4208641	c1ccc2c(NCCCCCSSCCCCCNc3c4c(nc5ccccc35)CCCC4)c3c(nc2c1)CCCC3	1.62
CHEMBL3600555	CC1=CC2Cc3nc4ccccc4c(NCCCCCCCCCCNc4c5c(nc6cc(Cl)ccc46)CCCC5)c3C(C1)C2	1.48
CHEMBL1651249	O=C(CCC1CCN(Cc2ccc(O)cc2)CC1)c1cc2c3c(ccn3C(=O)CC2)c1	0.489
CHEMBL128390	CCCCCCCNC(=O)Oc1cccc(CN(C)CCCOc2ccc3c(=O)c4cccnc4oc3c2)c1	42.0
CHEMBL128551	CCCCNC(=O)Oc1cccc(CN(C)CCCOc2ccc3ccc(=O)oc3c2)c1	14.0
CHEMBL3600553	CC1=CC2Cc3nc4ccccc4c(NCCCCCCCCCCNc4c5c(nc6ccccc46)CCCC5)c3C(C1)C2	6.46
CHEMBL1912060	O=C(CCCCc1cccs1)NCCCNc1c2c(nc3cc(Cl)ccc13)CCCC2	2.66
CHEMBL3353041	CC1=C[C@H]2Cc3nc4cc(Cl)ccc4c(NCCCCCCCCCCNc4c5c(nc6cc(Cl)ccc46)C[C@@H]4C=C(C)C[C@H]5C4)c3[C@@H](C1)C2	17.5
CHEMBL3600554	CC1=CC2Cc3nc4ccccc4c(NCCCCCCCCCCCCNc4c5c(nc6ccccc46)CCCC5)c3C(C1)C2	10.1
CHEMBL3600551	CC1=CC2Cc3nc4cc(Cl)ccc4c(NCCCCCCCCCCCCNc4c5c(nc6ccccc46)CCCC5)c3C(C1)C2	1.92
CHEMBL4204315	c1ccc2c(NCCCCC[Se][Se]CCCCCNc3c4c(nc5ccccc35)CCCC4)c3c(nc2c1)CCCC3	2.64
CHEMBL4213042	c1ccc2c(NCCCCC[Se]CCCCCNc3c4c(nc5ccccc35)CCCC4)c3c(nc2c1)CCCC3	2.25

Table S6. Structures and bioactivities of 144 BACE1 inhibitors

Molecule ChEMBL ID	SMILES	IC50 (nM)
CHEMBL2181911	CO[C@H](C)C(=O)N[C@@H](Cc1cccc(-c2nccs2)c1)[C@H](O)CN[C@H]1CC2(CCC2)Oc2ncc(CC(C)(C)C)cc21	5.5
CHEMBL2181890	COCC(=O)N[C@@H](Cc1cccc(-c2ncco2)c1)[C@H](O)CN[C@H]1CC2(CCC2)Oc2ncc(CC(C)(C)C)cc21	9.6
CHEMBL2181914	COC(OC)C(=O)N[C@@H](Cc1cccc(-c2nccs2)c1)[C@H](O)CN[C@H]1CC2(CCC2)Oc2ncc(CC(C)(C)C)cc21	9.8
CHEMBL2181910	CCOCC(=O)N[C@@H](Cc1cccc(-c2nccs2)c1)[C@H](O)CN[C@H]1CC2(CCC2)Oc2ncc(CC(C)(C)C)cc21	5.7
CHEMBL2181886	CC(=O)N[C@@H](Cc1ccc(OC(F)F)c(OC(F)F)c1)[C@H](O)CN[C@H]1CC2(CCC2)Oc2ncc(CC(C)(C)C)cc21	35.2
CHEMBL2181907	C#Cc1ccc(F)c(C[C@H](NC(=O)COC)[C@H](O)CN[C@H]2CC3(CCC3)Oc3ncc(CC(C)(C)C)cc32)c1	2.9
CHEMBL2181881	CC(C)(C)Cc1cnc2c(c1)[C@@H](NC[C@@H](O)[C@H](Cc1ccc3c(c1)OCO3)NC(=O)[C@@H]1CCCO1)CC1(CCC1)O2	20.4
CHEMBL3359755	CC1(C)C=C(c2cnc3c(c2)[C@]2(COC(N)=N2)c2cc(-c4cccnc4F)ccc2O3)CCO1	0.9
CHEMBL3354692	COC(C)(C)C#Cc1ccc2c(c1)[C@]1(COC(N)=N1)c1cc(-c3cncnc3)ccc1O2	7.0
CHEMBL3394044	NC1=N[C@@]2(CO1)c1cc(-c3cccnc3F)ccc1Oc1cnc([C@@H]3CCCCO3)cc12	18.5
CHEMBL2181829	CC(=O)N[C@@H](Cc1ccc2c(c1)OCCO2)[C@H](O)CN[C@H]1CC2(CCC2)Oc2ncc(CC(C)(C)C)cc21	22.8
CHEMBL3359760	CC(C)(C)C#Cc1cnc2c(c1)[C@]1(COC(N)=N1)c1cc(-c3cccnc3F)ccc1O2	4.7
CHEMBL3359761	COC(C)(C)C#Cc1cnc2c(c1)[C@]1(COC(N)=N1)c1cc(-c3cccnc3F)ccc1O2	4.6
CHEMBL3359758	N#Cc1ccc(-c2cnc3c(c2)[C@]2(COC(N)=N2)c2cc(-c4cccnc4F)ccc2O3)cc1	0.8
CHEMBL3354693	NC1=N[C@]2(CO1)c1cc(C3=CCOCC3)ccc1Oc1ccc(-c3cncnc3)cc12	4.0
CHEMBL3354702	CC(C)(C)COc1ccc2c(c1)[C@]1(COC(N)=N1)c1cc(-c3ccccn3)ccc1O2	23
CHEMBL3359753	NC1=N[C@@]2(CO1)c1cc(-c3cccnc3F)ccc1Oc1ncc(C3=CCCOC3)cc12	1.2
CHEMBL3354688	CC(C)(C)COc1ccc2c(c1)[C@]1(COC(N)=N1)c1cc(-c3cncnc3)ccc1O2	2.2
CHEMBL3394046	NC1=N[C@@]2(CO1)c1cc(-c3cccnc3F)ccc1Oc1cnc(N3CCOCC3)cc12	1.3
CHEMBL584917	CN1C(=O)C(c2cccc(-c3cccnc3)c2)(c2ccc3c(c2)OCCO3)N=C1N	89.95
CHEMBL3394041	NC1=N[C@@]2(CO1)c1cc(-c3cccnc3F)ccc1Oc1cnc(C3=CCCOC3)cc12	0.5
CHEMBL3394045	NC1=N[C@@]2(CO1)c1cc(-c3cccnc3F)ccc1Oc1cnc([C@H]3CCCCO3)cc12	2.2
CHEMBL3394050	NC1=N[C@@]2(CO1)c1cc(-c3cccnc3F)ccc1Oc1cnc(N3CC[C@H](F)C3)cc12	2.7
CHEMBL3394211	NC1=N[C@@]2(CO1)c1cc(-c3cccnc3F)ccc1Oc1c2cc(-c2cccnc2)nc1F	0.3
CHEMBL3394215	NC1=N[C@@]2(CO1)c1cc(-c3cccnc3F)ccc1Oc1c2cc(-c2cccnc2F)nc1F	1.2
CHEMBL3394218	NC1=N[C@@]2(CO1)c1cc(-c3cccnc3F)ccc1Oc1c2cc(C2=COCCC2)nc1F	0.2
CHEMBL3354701	CC1(COc2ccc3c(c2)[C@]2(COC(N)=N2)c2cc(-c4cncnc4)ccc2O3)COC1	7.0
CHEMBL2181901	COCC(=O)N[C@@H](Cc1ccc(F)c(-c2nccs2)c1)[C@H](O)CN[C@H]1CC2(CCC2)Oc2ncc(CC(C)(C)C)cc21	6.1
CHEMBL2181893	COCC(=O)N[C@@H](Cc1cccc(-c2cncs2)c1)[C@H](O)CN[C@H]1CC2(CCC2)Oc2ncc(CC(C)(C)C)cc21	5.2
CHEMBL3354708	COc1cncc(-c2ccc3c(c2)[C@@]2(COC(N)=N2)c2cc(OCC(C)(C)C)ccc2O3)c1	4.0
CHEMBL3354714	CC1(COc2ccc3c(c2)[C@]2(COC(N)=N2)c2cc(-c4cccnc4)cc(F)c2O3)COC1	71
CHEMBL3354690	CC(C)(C)CCc1ccc2c(c1)[C@]1(COC(N)=N1)c1cc(-c3cncnc3)ccc1O2	3.1
CHEMBL3354705	CC(C)(C)COc1ccc2c(c1)[C@]1(COC(N)=N1)c1cc(-c3ccc(F)nc3)ccc1O2	6.0
CHEMBL3394226	NC1=N[C@@]2(CO1)c1cc(-c3cccnc3F)ccc1Oc1c2cc(N2CCC(F)(F)C2)nc1F	0.2

Table S6. (*Cont.*)

Molecule ChEMBL ID	SMILES	IC_{50} (nM)
CHEMBL2407492	CC(C)(C)Cc1cnc2c(c1)[C@@H](NC[C@@H](O)[C@@H]1Cc3cccc(c3)CCCn3cc(ccc3=O)C(=O)N1)CC1(CCC1)O2	2.7
CHEMBL3359748	CC(C)(C)COc1cnc2c(c1)[C@]1(COC(N)=N1)c1cc(-c3cccnc3)ccc1O2	0.6
CHEMBL2181913	CCO[C@H](C)C(=O)N[C@@H](Cc1cccc(-c2nccs2)c1)[C@H](O)CN[C@H]1CC2(CCC2)Oc2ncc(CC(C)(C)C)cc21	16
CHEMBL384496	CC[C@H](NCC1Cc2cccc(c2)OCCNC(=O)c2cc(cc(N(C)S(C)(=O)=O)c2)C(=O)N1)C(=O)NCC(C)C	32
CHEMBL3354710	CC(C)(C)COc1ccc2c(c1)[C@]1(COC(N)=N1)c1cc(-c3c(F)ccnc3F)ccc1O2	0.5
CHEMBL3394056	Cc1cc(-c2cc3c(cn2)Oc2ccc(-c4cccnc4F)cc2[C@@]32COC(N)=N2)ccn1	2.4
CHEMBL3394213	Cc1ccc(-c2cc3c(c(F)n2)Oc2ccc(-c4cccnc4F)cc2[C@@]32COC(N)=N2)cn1	0.4
CHEMBL3394214	Cc1cc(-c2cc3c(c(F)n2)Oc2ccc(-c4cccnc4F)cc2[C@@]32COC(N)=N2)ccn1	0.8
CHEMBL3354718	NC1=N[C@@]2(CO1)c1cc(-c3cccnc3F)ccc1Oc1c(F)cc(C3=CCOCC3)cc12	0.3
CHEMBL3394223	NC1=N[C@@]2(CO1)c1cc(-c3cccnc3F)ccc1Oc1c2cc(C2CCOCC2)nc1F	0.7
CHEMBL2181884	CC(C)(C)Cc1cnc2c(c1)[C@@H](NC[C@@H](O)[C@H](Cc1ccc3c(c1)OCO3)NC(=O)c1cccnc1F)CC1(CCC1)O2	33.2
CHEMBL2181897	CC#Cc1cccc(C[C@H](NC(=O)COC)[C@H](O)CN[C@H]2CC3(CCC3)Oc3ncc(CC(C)(C)C)cc32)c1	15
CHEMBL2181898	COCC(=O)N[C@@H](Cc1ccc(-c2nccs2)cc1)[C@H](O)CN[C@H]1CC2(CCC2)Oc2ncc(CC(C)(C)C)cc21	29
CHEMBL3394210	NC1=N[C@@]2(CO1)c1cc(-c3cccnc3F)ccc1Oc1c2cc(-c2ccccc2)nc1F	0.7
CHEMBL3394228	Cc1cc(-c2cc3c(c(F)n2)Oc2ccc(-c4cccnc4F)cc2[C@@]32COC(N)=N2)on1	0.3
CHEMBL3394055	NC1=N[C@@]2(CO1)c1cc(-c3cccnc3F)ccc1Oc1cnc(-c3ccncc3)cc12	2.5
CHEMBL565914	NC1=NC(c2ccc(OC(F)(F)F)cc2)(c2cccc(-c3cccnc3F)c2)C2=NCCCN12	80
CHEMBL2181882	C[C@@H](F)C(=O)N[C@@H](Cc1ccc2c(c1)OCO2)[C@H](O)CN[C@H]1CC2(CCC2)Oc2ncc(CC(C)(C)C)cc21	28.9
CHEMBL2181880	CO[C@H](C)C(=O)N[C@@H](Cc1ccc2c(c1)OCO2)[C@H](O)CN[C@H]1CC2(CCC2)Oc2ncc(CC(C)(C)C)cc21	16.8
CHEMBL3265334	NC1=N[C@@]2(CO1)c1cc(-c3cc(F)cc(Cl)c3)ccc1O[C@@H]1COCC[C@H]12	60
CHEMBL3354700	CC(C)(C#N)COc1ccc2c(c1)[C@]1(COC(N)=N1)c1cc(-c3cncnc3)ccc1O2	9
CHEMBL3260839	CN1C(=O)C2(N=C1N)c1cc(-c3cccnc3F)ccc1Oc1c(F)cc(-c3ccnc(F)c3)cc12	0.2
CHEMBL2407489	CCc1ccc2c(c1)[C@@H](NC[C@@H](O)[C@@H]1Cc3cccc(c3)CCCCn3cc(cc(-c4ccccn4)c3=O)C(=O)N1)CC1(CCC1)O2	5.9
CHEMBL2407339	C=CCCCC(=O)N[C@@H](Cc1cccc(CC=C)c1)[C@H](O)CN[C@H]1CC2(CCC2)Oc2ncc(CC(C)(C)C)cc21	4.7
CHEMBL2407340	C=CCc1cccc(C[C@H](NC(=O)c2cc(Br)c(=O)n(CC=C)c2)[C@H](O)CN[C@H]2CC3(CCC3)Oc3ccc(CC)cc32)c1	81
CHEMBL1821820	Cc1ccccc1-c1ccc2nc(N)c(C[C@@H](C)C(=O)NCCC(C)(C)C)cc2c1	0.65
CHEMBL2181908	CC(=O)N[C@@H](Cc1cccc(-c2nccs2)c1)[C@H](O)CN[C@H]1CC2(CCC2)Oc2ncc(CC(C)(C)C)cc21	3.7
CHEMBL2181906	C#Cc1cc(F)cc(C[C@H](NC(=O)COC)[C@H](O)CN[C@H]2CC3(CCC3)Oc3ncc(CC(C)(C)C)cc32)c1	1.9
CHEMBL3394048	NC1=N[C@@]2(CO1)c1cc(-c3cccnc3F)ccc1Oc1cnc(N3CCC(F)(F)CC3)cc12	0.7
CHEMBL3394051	NC1=N[C@@]2(CO1)c1cc(-c3cccnc3F)ccc1Oc1cnc(N3CC[C@@H](F)C3)cc12	0.5
CHEMBL3354713	CC1(COc2cc(F)c3c(c2)[C@]2(COC(N)=N2)c2cc(-c4cccnc4)ccc2O3)COC1	1.0
CHEMBL2181915	CC(C)(C)Cc1cnc2c(c1)[C@@H](NC[C@@H](O)[C@H](Cc1cccc(-c3nccs3)c1)NC(=O)[C@H]1CCCO1)CC1(CCC1)O2	6.3
CHEMBL2181905	C#Cc1cc(C[C@H](NC(=O)COC)[C@H](O)CN[C@H]2CC3(CCC3)Oc3ncc(CC(C)(C)C)cc32)ccc1F	3.7

Table S6. (*Cont.*)

Molecule ChEMBL ID	SMILES	IC_{50} (nM)
CHEMBL3354697	CC(C)(O)COc1ccc2c(c1)[C@]1(COC(N)=N1)c1cc(-c3cncnc3)ccc1O2	7.0
CHEMBL3354706	CC(C)(C)COc1ccc2c(c1)[C@]1(COC(N)=N1)c1cc(-c3cncc(C#N)c3)ccc1O2	3.0
CHEMBL3354711	CC1(COc2ccc3c(c2)[C@]2(COC(N)=N2)c2cc(-c4cccnc4)ccc2O3)COC1	7.0
CHEMBL2181909	CCC(=O)N[C@@H](Cc1cccc(-c2nccs2)c1)[C@H](O)CN[C@H]1CC2(CCC2)Oc2ncc (CC(C)(C)C)cc21	11
CHEMBL2181904	C#Cc1cccc(C[C@H](NC(=O)COC)[C@H](O)CN[C@H]2CC3(CCC3)Oc3ncc(CC(C)(C)C)cc32)c1F	11
CHEMBL2181903	COCC(=O)N[C@@H](Cc1cc(-c2nccs2)ccc1F)[C@H](O)CN[C@H]1CC2(CCC2)Oc2ncc (CC(C)(C)C)cc21	9.5
CHEMBL2181902	COCC(=O)N[C@@H](Cc1cc(F)cc(-c2nccs2)c1)[C@H](O)CN[C@H]1CC2(CCC2)Oc2ncc (CC(C)(C)C)cc21	1.9
CHEMBL2181899	C#Cc1ccc(C[C@H](NC(=O)COC)[C@H](O)CN[C@H]2CC3(CCC3)Oc3ncc(CC(C)(C)C)cc32)cc1	47
CHEMBL2181889	COCC(=O)N[C@@H](Cc1cccc(-c2ccccn2)c1)[C@H](O)CN[C@H]1CC2(CCC2)Oc2ncc (CC(C)(C)C)cc21	9.3
CHEMBL2181916	CC(C)(C)Cc1cnc2c(c1)[C@@H](NC[C@@H](O)[C@H](Cc1cccc(-c3nccs3)c1)NC(=O) [C@@H]1CCCO1)CC1(CCC1)O2	9.2
CHEMBL2407491	CC(C)(C)Cc1cnc2c(c1)[C@@H](NC[C@@H](O)[C@@H]1Cc3cccc(c3)CCCCn3cc(ccc3=O)C(=O)N1) CC1(CCC1)O2	9.7
CHEMBL3359757	Cc1ccc(-c2cnc3c(c2)[C@]2(COC(N)=N2)c2cc(-c4cccnc4F)ccc2O3)cc1	0.9
CHEMBL3359747	CC(C)(C)COc1cnc2c(c1)[C@]1(COC(N)=N1)c1cc(-c3cncnc3)ccc1O2	0.7
CHEMBL3394049	NC1=N[C@@]2(CO1)c1cc(-c3cccnc3F)ccc1Oc1cnc(N3CCC(F)(F)C3)cc12	0.6
CHEMBL2181828	CC(C)(C)Cc1cnc2c(c1)[C@@H](NC[C@@H](O)[C@H](Cc1ccc3c(c1)OCO3)NC(=O)c1ccc(F)nc1) CC1(CCC1)O2	48.4
CHEMBL2181892	COCC(=O)N[C@@H](Cc1cccc(-c2nccs2)c1)[C@H](O)CN[C@H]1CC2(CCC2)Oc2ncc (CC(C)(C)C)cc21	4.1
CHEMBL2181887	CC(=O)N[C@@H](Cc1ccc2c(c1)OCO2)[C@H](O)CN[C@H]1CC2(CCC2)Oc2ncc(CC(C)(C)C)cc21	8.0
CHEMBL3359756	NC1=N[C@@]2(CO1)c1cc(-c3cccnc3F)ccc1Oc1ncc(N3CCOCC3)cc12	3.3
CHEMBL3359749	CC(C)(C)COc1cnc2c(c1)[C@]1(COC(N)=N1)c1cc(-c3cccnc3F)ccc1O2	0.4
CHEMBL3394039	NC1=N[C@@]2(CO1)c1cc(-c3cccnc3F)ccc1Oc1cnc(C3=CCOCC3)cc12	0.7
CHEMBL3394047	CC1(C)CN(c2cc3c(cn2)Oc2ccc(-c4cccnc4F)cc2[C@@]32COC(N)=N2)CCO1	4.0
CHEMBL3354715	CC1(COc2cc(F)c3c(c2)[C@]2(COC(N)=N2)c2cc(-c4cccnc4F)ccc2O3)COC1	0.4
CHEMBL2181896	C#Cc1cccc(C[C@H](NC(=O)COC)[C@H](O)CN[C@H]2CC3(CCC3)Oc3ncc(CC(C)(C)C)cc32)c1	3.4
CHEMBL2181891	COCC(=O)N[C@@H](Cc1cccc(- c2nccn2C)c1)[C@H](O)CN[C@H]1CC2(CCC2)Oc2ncc(CC(C)(C)C)cc21	64
CHEMBL3394054	NC1=N[C@@]2(CO1)c1cc(-c3cccnc3F)ccc1Oc1cnc(-c3cccnc3)cc12	2.5
CHEMBL2181894	COCC(=O)N[C@@H](Cc1cccc(- c2cscn2)c1)[C@H](O)CN[C@H]1CC2(CCC2)Oc2ncc(CC(C)(C)C)cc21	6.9
CHEMBL2030997	CC(=O)N[C@@H](Cc1ccc(F)cc1)[C@H](O)CN[C@H]1CC2(CCC2)Oc2ncc(CC(C)(C)C)cc21	5.0
CHEMBL2181895	COCC(=O)N[C@@H](Cc1cccc(- c2nc(C)cs2)c1)[C@H](O)CN[C@H]1CC2(CCC2)Oc2ncc(CC(C)(C)C)cc21	5.7
CHEMBL2181827	CC(C)(C)Cc1cnc2c(c1)[C@@H](NC[C@@H](O)[C@H](Cc1ccc3c(c1)OCO3)NC(=O)c1cccnc1)CC1 (CCC1)O2	26.1
CHEMBL2181883	CC(C)(C)Cc1cnc2c(c1)[C@@H](NC[C@@H](O)[C@H](Cc1ccc3c(c1)OCO3)NC(=O)c1ccccc1F) CC1(CCC1)O2	76.6

Table S6. Structures and bioactivities of 144 BACE1 inhibitors

Molecule ChEMBL ID	SMILES	IC_{50} (nM)
CHEMBL3359754	NC1=N[C@@]2(CO1)c1cc(-c3cccnc3F)ccc1Oc1ncc(C3CCOCC3)cc12	5.6
CHEMBL3394040	CC1(C)C=C(c2cc3c(cn2)Oc2ccc(-c4cccnc4F)cc2[C@@]32COC(N)=N2)CCO1	0.9
CHEMBL1271450	CN1C(=O)[C@@](c2ccc(OCCF)cc2)(c2cccc(-c3cccnc3F)c2)N=C1N	30
CHEMBL3394058	NC1=N[C@]2(CO1)c1cc(C3=CCOCC3)ccc1Oc1ccc(-c3cccnc3F)cc12	2.6
CHEMBL3394212	NC1=N[C@@]2(CO1)c1cc(-c3cccnc3F)ccc1Oc1c2cc(-c2ccncc2)nc1F	0.4
CHEMBL3394227	NC1=N[C@@]2(CO1)c1cc(-c3cccnc3F)ccc1Oc1c2cc(N2CC[C@@H](F)C2)nc1F	0.2
CHEMBL3354689	CC(C)(C)C#Cc1ccc2c(c1)[C@]1(COC(N)=N1)c1cc(-c3cncnc3)ccc1O2	2
CHEMBL3354695	CC1(C)CN(c2ccc3c(c2)[C@]2(COC(N)=N2)c2cc(-c4cncnc4)ccc2O3)CCO1	11
CHEMBL3354699	CC(C)(F)COc1ccc2c(c1)[C@]1(COC(N)=N1)c1cc(-c3cncnc3)ccc1O2	8
CHEMBL3354709	CC(C)(C)COc1ccc2c(c1)[C@]1(COC(N)=N1)c1cc(-c3cccnc3F)ccc1O2	0.8
CHEMBL2181912	CO[C@@H](C)C(=O)N[C@@H](Cc1cccc(-c2nccs2)c1)[C@H](O)CN[C@H]1CC2(CCC2) Oc2ncc(CC(C)(C)C)cc21	7.2
CHEMBL2181830	COCC(=O)N[C@@H](Cc1ccc2c(c1)OCO2)[C@H](O)CN[C@H]1CC2(CCC2)Oc2ncc(CC(C)(C)C)cc21	5.4
CHEMBL3359752	NC1=N[C@@]2(CO1)c1cc(-c3cccnc3F)ccc1Oc1ncc(C3=CCOCC3)cc12	1.5
CHEMBL3354707	CC(C)(C)COc1ccc2c(c1)[C@]1(COC(N)=N1)c1cc(-c3cncc(F)c3)ccc1O2	4.0
CHEMBL3394042	NC1=N[C@@]2(CO1)c1cc(-c3cccnc3F)ccc1Oc1cnc(C3CCOCC3)cc12	7.1
CHEMBL3394222	NC1=N[C@@]2(CO1)c1cc(-c3cccnc3F)ccc1Oc1c2cc(C2CCCOC2)nc1F	1
CHEMBL3394225	NC1=N[C@@]2(CO1)c1cc(-c3cccnc3F)ccc1Oc1c2cc(N2CCC(F)(F)CC2)nc1F	0.3
CHEMBL2181900	COCC(=O)N[C@@H](Cc1cccc(- c2nccs2)c1F)[C@H](O)CN[C@H]1CC2(CCC2)Oc2ncc(CC(C)(C)C)cc21	71
CHEMBL2181888	COCC(=O)N[C@@H](Cc1cccc(- c2ccncc2)c1)[C@H](O)CN[C@H]1CC2(CCC2)Oc2ncc(CC(C)(C)C)cc21	33
CHEMBL3359762	CC(C)(O)C#Cc1cnc2c(c1)[C@]1(COC(N)=N1)c1cc(-c3cccnc3F)ccc1O2	1.2
CHEMBL3359751	COC(C)(C)COc1cnc2c(c1)[C@]1(COC(N)=N1)c1cc(-c3cccnc3F)ccc1O2	1.8
CHEMBL2181917	C[C@@H](O)C(=O)N[C@@H](Cc1cccc(- c2nccs2)c1)[C@H](O)CN[C@H]1CC2(CCC2)Oc2ncc(CC(C)(C)C)cc21	3.2
CHEMBL3394057	NC1=N[C@@]2(CO1)c1cc(-c3cccnc3F)ccc1Oc1cnc(-c3ccnc(F)c3)cc12	0.9
CHEMBL3394221	NC1=N[C@@]2(CO1)c1cc(-c3cccnc3F)ccc1Oc1c2cc(C2=CCOCC2)nc1F	0.3
CHEMBL3394052	NC1=N[C@@]2(CO1)c1cc(-c3cccnc3F)ccc1Oc1cnc(-c3ccccc3)cc12	1.4
CHEMBL4294221	NC1=N[C@@]2(CO1)c1cc(NC(=O)c3ccc(Cl)cn3)ccc1Oc1cnc(C3=CCCOC3)cc12	0.62
CHEMBL3394219	NC1=N[C@@]2(CO1)c1cc(-c3cccnc3F)ccc1Oc1c2cc(C2=CCCCO2)nc1F	0.4
CHEMBL3394043	NC1=N[C@@]2(CO1)c1cc(-c3cccnc3F)ccc1Oc1cnc(C3CCCOC3)cc12	11.3
CHEMBL3394217	NC1=N[C@@]2(CO1)c1cc(-c3cccnc3F)ccc1Oc1c2cc(-c2ccnc(F)c2)nc1F	0.4
CHEMBL3394220	NC1=N[C@@]2(CO1)c1cc(-c3ncccc3F)ccc1Oc1c2cc(C2=CCCOC2)nc1F	0.3
CHEMBL3354704	CC(C)(C)COc1ccc2c(c1)[C@]1(COC(N)=N1)c1cc(-c3cccnc3)ccc1O2	4.0
CHEMBL3640264	COc1nc(N2C[C@H]3C(=O)N(C)C(N)=N[C@@]3(c3ccccc3F)C2)nc(C)c1Cl	32
CHEMBL3359759	NC1=N[C@@]2(CO1)c1cc(-c3cccnc3F)ccc1Oc1ncc(-c3ccnc(F)c3)cc12	2.3
CHEMBL3394224	NC1=N[C@@]2(CO1)c1cc(-c3cccnc3F)ccc1Oc1c2cc(N2CCOCC2)nc1F	0.3
CHEMBL3394053	NC1=N[C@@]2(CO1)c1cc(-c3cccnc3F)ccc1Oc1cnc(-c3ccccn3)cc12	3.8
CHEMBL3354691	CC(C)(O)C#Cc1ccc2c(c1)[C@]1(COC(N)=N1)c1cc(-c3cncnc3)ccc1O2	2.0
CHEMBL3354712	CC1(COc2cc3c(cc2F)Oc2ccc(-c4cccnc4)cc2[C@@]32COC(N)=N2)COC1	4.0
CHEMBL4279496	CC1(C#Cc2cncc(-c3ccc4c(c3)[C@@]3(COC(N)=N3)c3cc(C5=CCOCC5)ncc3O4)c2)COC1	5.5
CHEMBL4279064	NC1=N[C@@]2(CO1)c1cc(NC(=O)c3ccc(Cl)cn3)ccc1Oc1cnc(C3CC3)cc12	3.6

Table S6. (*Cont.*)

Molecule ChEMBL ID	SMILES	IC_{50} (nM)
CHEMBL4284110	CC#Cc1cncc(-c2ccc3c(c2)[C@@]2(COC(N)=N2)c2cc(C4=CCOCC4)ncc2O3)c1	0.31
CHEMBL4282964	NC1=N[C@@]2(CO1)c1cc(-c3cncc(C#CC4CC4)c3)ccc1Oc1cnc(C3=CCOCC3)cc12	1.8
CHEMBL4276834	CC#Cc1cncc(-c2ccc3c(c2)[C@@]2(COC(N)=N2)c2cc(C4=CCCOC4)ncc2O3)c1	0.46
CHEMBL4287483	CC#Cc1cncc(-c2ccc3c(c2)[C@@]2(COC(N)=N2)c2cc(-c4ccnc(F)c4)ncc2O3)c1	0.43
CHEMBL4285211	NC1=N[C@@]2(CO1)c1cc(-c3cccc(F)n3)ccc1Oc1cnc(-c3ccnc(F)c3)cc12	0.92
CHEMBL4290822	NC1=N[C@@]2(CO1)c1cc(-c3cncc(C#CCO)c3)ccc1Oc1cnc(C3=CCOCC3)cc12	0.58
CHEMBL4286331	NC1=N[C@@]2(CO1)c1cc(NC(=O)c3ccc(Cl)cn3)ccc1Oc1cnc(C3CCOCC3)cc12	0.57

Table S7. Comparison of this study with previous published works on AChE

Year	Methods	Molecular descriptors	Database	QSAR Model performance	References
2007	PLS Regression using 3D-QSAR (COMFA)	The steric and electrostatic fields; sp3 carbon atom with a charge of +1; Lennard-Jones potential…	38 phenyl pentenones	R^2cv=0.629, non-cross-validated R^2=0.972, SE=0.331, and F=72.41	Liu *et al.* [1]
2008	PLS Regression using 3D-QSAR (COMFA, COMSIA)	The steric and electrostatic fields; sp3 carbon atom with a charge of +1; Lennard-Jones potential…	78 chemically diverse molecules (aminoindanes, tetralenes and phenethyl amines)	CoMFA q^2 = 0.733, r^2 = 0.967, predictive r^2 = 0.732, CoMSIA q^2 = 0.641, r^2 = 0.936, predictive r^2 = 0.812	Roy *et al.* [2]
2009	PLS Regression using 3D-QSAR (COMFA, COMSIA)	The steric and electrostatic fields; sp3 carbon atom with a charge of +1; Lennard-Jones potential…	52 carbamates	COMFA: Q^2LOO = 0.573 and R^2 = 0.972 COMSIA: Q^2LOO = 0.723 and R^2 = 0.950	Chaudhaery *et al.* [3]
2012	Classification (SVM)	211 ADRIANA.Code and 334 MOE descriptors	714 hAChE inhibitors	Matthews Correlation Coefficient (MCC) of 0.99 and a prediction accuracy (Q) of 99.66%	Wang *et al.* [4]
2012	Support Vector Machine (SVM)	1024 radial distribution functions (RDF) descriptors were calculated using ADRIANA.Code 2.2.2	404 AChE inhibitors	Model 1A: R^2 = 0.9; Q^2 = 0.9 Model 1B: R^2 = 0.9; Q^2 = 0.9 Model 2A: R^2 = 0.9; Q^2 = 0.91 Model 2B: R^2 = 0.9; Q^2 = 0.91	Yan and Wang [5]
2012	Stepwise multiple linear regression	24 docking descriptors (Glide score, Gold score, Chem score, ASP score, PMF score, and DOCK score)	91 molecules belonging to 9 different structural classes of heterocyclic compounds	The best model: R^2 = 0.938, Q^2 = 0.925, R^2 pred = 0.919, R^2 m(overall) = 0.936	Deb *et al.* [6]
2012	PLS Regression using 3D-QSAR	Steric (Lennard-Jones 6–12 potential) field and electrostatic	72 Tacrine-like inhibitors	CoMFA model: Q^2 = 0.686, R^2 = 0.948; CoMSIA model:	Chen *et al.* [7]

	(COMFA, COMSIA)	(Coulombic potential) field energies, sp3-carbon atom having a charge of +1 and a van der Waals radius of 1.52 Å		$Q^2 = 0.756$, $R^2 = 0.907$	
2013	Least squares support vector regression (LS-SVR)	Energy and interaction descriptors by AutoDock 4.2 and BINding ANAlyzer (BINANA)	68 4-[(diethylamino)methyl]-phenol and 2,4-disubstituted pyrimidine derivatives	The best model: $Q^2 = 0.790$, $R^2 = 0.860$	Gharaghani *et al.* [8]
2013	PLS Regression using 3D-QSAR (COMFA)	The steric and electrostatic fields; sp3 carbon atom with a charge of +1; Lennard-Jones potential…	41 oxoaporphine and oxoisoaporphine derivatives	CoMFA model ($Q^2 = 0.856$ and $R^2 = 0.986$) test-set validation ($Q^2 = 0.873$, $Q^2 = 0.937$, and slope k = 0.902)	Li *et al.* [9]
2014	Stepwise multiple linear regression	Dragon 2D-descriptors	10 datasets (92 Tacrine-like inhibitors)	10 QSAR models $R^2 = 0.90$-0.99; R^2LOO = 0.84-7	Wong *et al.* [10]
2014	Partial Least Square, G-QSAR model	2-D descriptors	27 flavonoid derivatives	Training set ($R^2 = 0.8070$, $Q^2 = 0.7088$); test set (pred_$R^2 = 0.8131$)	Vats *et al.* [11]
2015	3D-QSAR	3D-fingerprints	89 reversible and irreversible AChEIs	Correlation value ($R^2 = 0.93$); cross-validated correlation coefficient ($Q^2 = 0.89$), and external validation results (n = 26, R2 = 0.89, and MAE = 0.38 log units)	Lee and Barron [12]
2015	PLS Regression using 3D-QSAR (COMFA, COMSIA)	Steric and H-bond fields, and electrostatic fields	60 molecules	CoMFA model: $Q^2 = 0.552$; $R^2 = 0.983$ CoMSIA model: $Q^2 = 0.581$ and $R^2 = 0.989$	Zhou *et al.* [13]
2017	Multiple Linear Regression (MLR), Genetic Function Approximation (GFA) and Multilayer Perceptron Network (MLP)	2489 descriptors from the online program E-Dragon 1.0 and Molecular Operating Environment (MOE) software	99 N-benzylpiperidine derivatives	MLR: $Q^2 = 0.831$, $R^2 = 0.843$ MLP: $Q^2 = 0.737$, $R^2 = 0.869$ GFA: $Q^2 = 0.842$, $R^2 = 0.875$	Bitam *et al.* [14]
2018	Multiple linear regression (MLR)	10 descriptors by Hyperchem (version 8.0.8, Hypercube, Inc.), Gaussian 09 software	36 4-[(diethylamino)methyl]-phenol derivatives	The best model: R^2adj = 0.660 $Q^2 = 0.70$	Daoud *et al.* [15]
2021	Random forest (RF) classifier	1665 Descriptors from DRAGON v5.4 software and 128 descriptors from Volsurf + v.1.04 software	6227 compounds in ChEMBL, 314 compounds in NPASS	True positive (TP) and true negative (TN) more than 0.8, AUC > 0.9	López *et al.* [16]

2022	Support vector machine (SVM), k-nearest neighbor (k-NN) and random forest (RF)	1444 descriptors (1D and 2D descriptors) of PaDEL software 245-bit fngerprints (166-bit MACCS key and 79-bit estate fingerprints	5692 molecules from ChEMBL	RF model: Accuracy = 0.84, MCC = 0.67 k-NN: Accuracy = 0.816, MCC = 0.62 SVM: Accuracy = 0.84, MCC = 0.67	Sandhu *et al.* [17]
2022	QSAR-SVM classification	0-3D molecular descriptors of the DRAGON software	1975 compounds	Accuracy of 88.63% for training set, 81.13% for cross-validation experiment and 81.15% for prediction set.	Cañizares-Carmenatea *et al.* [18]
2023	-Rule-of-Thumb -Classification algorithms: CART, CHAID, and RF	1100 and 1151 0-2D descriptors calculated using Dragon 6.0	ChEMBL data-bases including 1975 AChE in-hibitors and 1549 BACE1 in-hibitors	AChE models: - 4 Rules with accuracy of 0.77-0.89 - 3 models: R2 (training) = 0.85-0.87; Q2 (test) = 0.83-0.86	Current study

Table S8. Comparison of this study with previous published works on BACE1

Year	Methods	Molecular descriptors	Database	QSAR Model performance	References
2013	CoMFA, CoMSIA, Partial least square (PLS)	The steric and electrostatic fields; sp3 carbon atom with a charge of +1; Lennard-Jones potential…	106 compounds	$R^2 = 0.94 – 1.00$, $Q^2 = 0.73 – 0.79$, $R^2_{pred} = 0.71 – 0.80$	Hossain *et al.* [19]
2014	Multiple linear regression (MLR)	2D descriptors and docking-based descriptors	50 structurally diverse BACE-1 inhibitors	$R^2 = 0.96$, $R^2_{pred} = 0.91$	Chakraborty *et al.* [20]
2014	Multiple linear regression (MLR)	2D descriptors by DRAGON software	21 gallic acid derivatives	$R^2 = 0.893$, $R^2_{adj} = 0.866$, $R^2_{CV} = 0.826$	Gupta K. [21]
2015	Multi-parameter regression	539 descriptors: constitutional, topological, geometrical, electrostatics, quantum chemical, and thermodynamic descriptors calculated by CODESSA	31 natural compounds	$R^2_{CV} = 0.85$, $R^2 = 0.89$	Das *et al.* [22]
2016	Genetic algorithm (GA), multiple linear regression (MLR), partial least squares (PLS)	644 2D descriptors employing Cerius 2 version 4.10, PaDEL Descriptor version 2.11, and Dragon 6 software	91 cyclic sulfone (or sulfoxide) hydroxyethylamines	$R^2_{training} = 0.8318$, $R^2_{adj} = 0.8135$, $Q^2 = 0.764$, $R^2_{test} = 0.8133$, $R^2_{pred} = 0.801$	Ambure and Roy [23]
2018	CoMFA, CoMSIA, Partial least square (PLS)	Steric and H-bond fields, and electrostatic fields	41 molecules of biaryl aminothiazine BACE1 inhibitors	$Q^2 > 0.5$	Liu *et al.* [24]

2019	Partial least squares (PLS)	284 Topological, Physicochemical, and geometric descriptors	18 compounds	$R^2 = 0.9738$, $R^2_{pred} = 0.898$, $Q^2_{LOOCV} = 0.898$, $Q^2_{test} = 0.6057$	Joseph *et al.* [25]
2019	Partial least square regression (PLS-R) and Artificial Neural Network (ANN)	327 molecular descriptors calculated using QuaSAR module of MOE package	35 isonicotinamides derivatives	$R^2_{PLS\text{-}R} = 0.84$, $R^2_{adj\ (PLS\text{-}R)} = 0.82$, $R^2_{ANN} = 0.8 - 0.9$	El Aissouq *et al.* [26]
2020	Multiple linear regression (MLR)	molecular weight, LogP and polarizability	34 known inhibitors of BACE1	$R^2 = 0.9992$, $R^2_{adj} = 0.9969$	Chetia *et al.* [27]
2021	Naïve Bayesian (NB), nearest known neighbours (kNN), support vector machine (SVM), random forest (RF) and gradient-boosted algorithms (XGB)	2 types of descriptors: molecular property and fingerprints	3536 diverse BACE1 inhibitors	$F1_{NB} = 0.74$, $F1_{kNN} = 0.85$, $F1_{SVM} = 0.86$, $F1_{RF} = 0.87$ and $F1_{XGB} = 0.87$	Singh *et al.* [28]
2022	GA-MLR (Genetic Algorithm-Multi-linear Regression)	3,281 molecular descriptors	552 molecules from ChEMBL database	$R^2 = 0.82$, $R^2_{adj} = 0.8168$, $Q^2_{LOO} = 0.81$	Mukerjee *et al.* [29]
2023	-Rule-of-Thumb<br>-Classification algorithms: CART, CHAID, and RF	1100 and 1151 0-2D descriptors calculated using Dragon 6.0	ChEMBL data-bases including 1975 AChE in-hibitors and 1549 BACE1 in-hibitors	BACE1 models:<br>- 3 Rules with accuracy of 0.75-0.98<br>- 3 models: R2 (training) = 0.82-0.85; Q2 (test) = 0.80-0.83	Current study

References

1. Liu, A.; Guang, H.; Zhu, L.; Du, G.; Lee, S.M.Y.; Wang, Y. 3D-QSAR analysis of a new type of acetylcholinesterase inhibitors. *Sci. China C. Life Sci.* **2007**, *50*, 726-730. https://doi.org/10.1007/s11427-007-0094-1.
2. Roy, K.K.; Dixit, A.; Saxena, A.K. An investigation of structurally diverse carbamates for acetylcholinesterase (AChE) inhibition using 3D-QSAR analysis. *J. Mol. Graph. Model.* **2008**, *27*, 197-208. https://doi.org/10.1016/j.jmgm.2008.04.006.
3. Chaudhaery, S.S.; Roy, K.K.; Saxena, A.K. Consensus Superiority of the Pharmacophore-Based Alignment, Over Maximum Common Substructure (MCS): 3D-QSAR Studies on Carbamates as Acetylcholinesterase Inhibitors. *J. Chem. Inf. Model.* **2009**, *49*, 1590-1601. https://doi.org/10.1021/ci900049e.
4. Wang, K.; Hu, X.; Wang, Z.; Yan, A. Classification of Acetylcholinesterase Inhibitors and Decoys by a Support Vector Machine. *Comb. Chem. High Throughput Screen.* **2012**, *15*, 492-502. http://dx.doi.org/10.2174/138620712800563891.
5. Yan, A.; Wang, K. Quantitative structure and bioactivity relationship study on human acetylcholinesterase inhibitors. *Bioorg. Med. Chem. Lett.* **2012**, *22*, 3336-3342. https://doi.org/10.1016/j.bmcl.2012.02.108.
6. Deb, P.K.; Sharma, A.; Piplani, P.; Akkinepally, R.R. Molecular docking and receptor-specific 3D-QSAR studies of acetylcholinesterase inhibitors. *Mol. Divers.* **2012**, *16*, 803-823. https://doi.org/10.1007/s11030-012-9394-x.
7. Chen, N.; Liu, C.; Zhao, L.; Zhang, H. 3D-QSAR study of multi-target-directed AchE inhibitors based on autodocking. *Med. Chem. Res.* **2012**, *21*, 245-256. https://doi.org/10.1007/s00044-010-9516-x.
8. Gharaghani, S.; Khayamian, T.; Ebrahimi, M. Molecular dynamics simulation study and molecular docking descriptors in structure-based QSAR on acetylcholinesterase (AChE) inhibitors. *SAR QSAR Environ. Res.* **2013**, *24*, 773-794. https://doi.org/10.1080/1062936X.2013.792877.
9. Li, Y.-P.; Weng, X.; Ning, F.-X.; Ou, J.-B.; Hou, J.-Q.; Luo, H.-B.; Li, D.; Huang, Z.-S.; Huang, S.-L.; Gu, L.-Q. 3D-QSAR studies of azaoxoisoaporphine, oxoaporphine, and oxoisoaporphine derivatives as anti-AChE and anti-AD agents by the CoMFA method. *J. Mol. Graph. Model.* **2013**, *41*, 61-67. https://doi.org/10.1016/j.jmgm.2013.02.003.
10. Wong, K.Y.; Mercader, A.G.; Saavedra, L.M.; Honarparvar, B.; Romanelli, G.P.; Duchowicz, P.R. QSAR analysis on tacrine-related acetylcholinesterase inhibitors. *Journal of biomedical science* **2014**, *21*, 84. 10.1186/s12929-014-0084-0.

11. Vats, C.; Dhanjal, J.K.; Goyal, S.; Bharadvaja, N.; Grover, A. Computational design of novel flavonoid analogues as potential AChE inhibitors: analysis using group-based QSAR, molecular docking and molecular dynamics simulations. *Struct. Chem.* **2015**, *26*, 467-476. https://doi.org/10.1007/s11224-014-0494-3.
12. Lee, S.; Barron, M.G. Development of 3D-QSAR Model for Acetylcholinesterase Inhibitors Using a Combination of Fingerprint, Molecular Docking, and Structure-Based Pharmacophore Approaches. *Toxicol. Sci.* **2015**, *148*, 60-70. https://doi.org/10.1093/toxsci/kfv160.
13. Zhou, A.; Hu, J.; Wang, L.; Zhong, G.; Pan, J.; Wu, Z.; Hui, A. Combined 3D-QSAR, molecular docking, and molecular dynamics study of tacrine derivatives as potential acetylcholinesterase (AChE) inhibitors of Alzheimer's disease. *J. Mol. Model.* **2015**, *21*, 277. 10.1007/s00894-015-2797-8.
14. Bitam, S.; Hamadache, M.; Hanini, S. QSAR model for prediction of the therapeutic potency of N-benzylpiperidine derivatives as AChE inhibitors. *SAR QSAR Environ. Res.* **2017**, *28*, 471-489. https://doi.org/10.1080/1062936X.2017.1331467.
15. Daoud, I.; Melkemi, N.; Salah, T.; Ghalem, S. Combined QSAR, molecular docking and molecular dynamics study on new Acetylcholinesterase and Butyrylcholinesterase inhibitors. *Comput. Biol. Chem.* **2018**, *74*, 304-326. https://doi.org/10.1016/j.compbiolchem.2018.03.021.
16. López, A.F.F.; Martínez, O.M.M.; Hernández, H.F.C. Evaluation of Amaryllidaceae alkaloids as inhibitors of human acetylcholinesterase by QSAR analysis and molecular docking. *J. Mol. Struct.* **2021**, *1225*, 129142. https://doi.org/10.1016/j.molstruc.2020.129142.
17. Sandhu, H.; Kumar, R.N.; Garg, P. Machine learning-based modeling to predict inhibitors of acetylcholinesterase. *Mol. Divers.* **2022**, *26*, 331-340. https://doi.org/10.1007/s11030-021-10223-5.
18. Cañizares-Carmenate, Y.; Nam, N.H.; Díaz-Amador, R.; Thuan, N.T.; Dung, P.T.P.; Torrens, F.; Pham-The, H.; Perez-Gimenez, F.; Castillo-Garit, J.A. Ligand-based discovery of new potential acetylcholinesterase inhibitors for Alzheimer's disease treatment. *SAR QSAR Environ. Res.* **2022**, *33*, 49-61. https://doi.org/10.1080/1062936X.2022.2025615.
19. Hossain, T.; Islam, M.A.; Pal, R.; Saha, A. Exploring structural requirement and binding interactions of β-amyloid cleavage enzyme inhibitors using molecular modeling techniques. *Med. Chem. Res.* **2013**, *22*, 4766-4774. https://doi.org/10.1007/s00044-013-0481-z.
20. Chakraborty, S.; Ramachandran, B.; Basu, S. Encompassing receptor flexibility in virtual screening using ensemble docking-based hybrid QSAR: discovery of novel phytochemicals for BACE1 inhibition. *Mol. BioSyst.* **2014**, *10*, 2684-2692. https://doi.org/10.1039/C4MB00307A.
21. Gupta, K. QSAR Studies on Gallic Acid Derivatives and Molecular Docking Studies of Bace1 Enzyme – A Potent Target of Alzheimer Disease. *J. Biosci. Bioeng.* **2014**, *1*, 11-27.
22. Das, S.; Majumder, T.; Sarkar, A.; Mukherjee, P.; Basu, S. Flavonoids as BACE1 inhibitors: QSAR modelling, screening and in vitro evaluation. *Int. J. Biol. Macromol.* **2020**, *165*, 1323-1330. https://doi.org/10.1016/j.ijbiomac.2020.09.232.
23. Ambure, P.; Roy, K. Understanding the structural requirements of cyclic sulfone hydroxyethylamines as hBACE1 inhibitors against Aβ plaques in Alzheimer's disease: a predictive QSAR approach. *RSC Adv.* **2016**, *6*, 28171-28186. https://doi.org/10.1039/C6RA04104C.
24. Liu, J.; Ni, J.; Wang, X.; Bi, Y. 3D-QSAR Analysis and Molecular Docking Study on Biaryl Aminothiazine BACE1 Inhibitor. *China Pharm.* **2018**, *12*, 1335-1339.
25. Joseph, O.A.; Babatomiwa, K.; Niyi, A.; Olaposi, O.; Olumide, I. Molecular Docking and 3D Qsar Studies of C000000956 as a Potent Inhibitor of Bace-1. *Drug Res. (Stuttg)* **2019**, *69*, 451-457. https://doi.org/10.1055/a-0849-9377.
26. Aissouq, A.E.; Toufik, H.; Lamchouri, F.; Stitou, M.; Ouammou, A. QSAR study of isonicotinamides derivatives as Alzheimr's disease inhibitors using PLS-R and ANN methods. In Proceedings of the 2019 International Conference on Intelligent Systems and Advanced Computing Sciences (ISACS), 26-27 Dec. 2019, 2019; pp. 1-7.
27. Chetia, P.; Mazumder, M.K.; Mahanta, S.; De, B.; Dutta Choudhury, M. A novel phytochemical from Dipteris wallichii inhibits human β-secretase 1: Implications for the treatment of Alzheimer's disease. *Med. Hypotheses* **2020**, *143*, 109839. https://doi.org/10.1016/j.mehy.2020.109839.
28. Singh, R.; Ganeshpurkar, A.; Ghosh, P.; Pokle, A.V.; Kumar, D.; Singh, R.b.; Singh, S.K.; Kumar, A. Classification of beta-site amyloid precursor protein cleaving enzyme 1 inhibitors by using machine learning methods. *Chem. Biol. Drug Des.* **2021**, *98*, 1079-1097. https://doi.org/10.1111/cbdd.13965.
29. Mukerjee, N.; Das, A.; Jawarkar, R.D.; Maitra, S.; Das, P.; Castrosanto, M.A.; Paul, S.; Samad, A.; Zaki, M.E.A.; Al-Hussain, S.A.; et al. Repurposing food molecules as a potential BACE1 inhibitor for Alzheimer's disease. *Front. Aging Neurosci.* **2022**, *14*. https://doi.org/10.3389/fnagi.2022.878276.
